# Synthesis of Platinum(II) N-Heterocyclic Carbenes Based on Adenosine

**DOI:** 10.3390/molecules26175384

**Published:** 2021-09-04

**Authors:** Maria Inês P. S. Leitão, Giulia Francescato, Clara S. B. Gomes, Ana Petronilho

**Affiliations:** 1 ITQB-NOVA—Instituto de Tecnologia Química e Biológica António Xavier, Universidade Nova de Lisboa, Avd da Republica, 2780-157 Oeiras, Portugal; inesleitao@itqb.unl.pt (M.I.P.S.L.); giulia@itqb.unl.pt (G.F.); 2 LAQV-REQUIMTE, Department of Chemistry, NOVA School of Science and Technology, Universidade Nova de Lisboa, 2829-516 Caparica, Portugal; clara.gomes@fct.unl.pt; 3 Associate Laboratory i4 HB—Institute for Health and Bioeconomy, School of Science and Technology, NOVA University Lisbon, 2819–516 Caparica, Portugal; 4 UCIBIO—Applied Molecular Biosciences Unit, Department of Chemistry, School of Science and Technology, NOVA University Lisbon, 2819-516 Caparica, Portugal

**Keywords:** N-heterocyclic carbenes, adenosine, platinum complexes, organometallic nucleosides, ^195^Pt NMR, cis-influence

## Abstract

Organometallic derivatization of nucleosides is a highly promising strategy for the improvement of the therapeutic profile of nucleosides. Herein, a methodology for the synthesis of metalated adenosine with a deprotected ribose moiety is described. Platinum(II) N-heterocyclic carbene complexes based on adenosine were synthesized, namely N-heterocyclic carbenes bearing a protected and unprotected ribose ring. Reaction of the 8-bromo-2′,3′,5′-tri-*O*-acetyladenosine with Pt(PPh_3_)_4_ by C8−Br oxidative addition yielded complex **1**, with a Pt^II^ centre bonded to C-8 and an unprotonated N7. Complex **1** reacted at N7 with HBF_4_ or methyl iodide, yielding protic carbene **2** or methyl carbene **3**, respectively. Deprotection of **1** to yield **4** was achieved with NH_4_OH. Deprotected compound **4** reacted at N7 with HCl solutions to yield protic NHC **5** or with methyl iodide yielding methyl carbene **6**. Protic N-heterocyclic carbene **5** is not stable in DMSO solutions leading to the formation of compound **7**, in which a bromide was replaced by chloride. The cis-influence of complexes **1**–**7** was examined by ^31^P{^1^H} and ^195^Pt NMR. Complexes **2**, **3**, **5**, **6** and **7** induce a decrease of ^1^*J*_Pt,P_ of more than 300 Hz, as result of the higher cis-influence of the N-heterocyclic carbene when compared to the azolato ligand in **1** and **4**.

## 1. Introduction

Organometallic functionalization is a very promising strategy to improve the biological activity of drugs, of which ferrocifen [1] and ferroquine are very good examples [2]. Indeed, the organometallic derivatization of nucleosides, often used in the clinic in treatment of several diseases, has the potential to not only improve their therapeutic profile but also to promote additional modes of action due to the incorporation of the metal. Nucleosides currently used as drugs generally contain a modified sugar, a modified nucleobase, or both, and often target the synthesis of nucleic acids. However, modified nucleosides can also target specific proteins involved in the regulation of a given biological process. One such case is the development of adenosine antagonists. Adenosine is a crucial extracellular modulator, involved in several biological processes. It is released under stress conditions and has an important regulatory role. This multi-signaling molecule restores the intracellular energy levels and mediates tissue protection by adjusting the oxygen levels, thus acting as an anti-inflammatory agent, and stimulating angiogenesis [3]. These functions are achieved by activating specific adenosine receptors (ARs), which represent pharmacological targets for the treatment of several human diseases, including neurological, inflammatory or ischaemic conditions. Several agonists and antagonists of ARs have been developed, mostly derived from adenosine itself through functionalization of the purine ring, or at the sugar moiety [3,4]. In line with this, we became interested in the development of organometallic nucleosides based on adenosine.

We have recently reported the synthesis of platinum and palladium NHCs based on guanosine derivatives [5,6]. Similarly, Hahn’s group described a synthetic methodology for the synthesis of guanosine and adenosine complexes with palladium [7]. These compounds can be obtained by C-X [5,7] or C-H [6] oxidative addition to Platinum(0) or palladium(0) centres. We showed that metalated nucleosides maintain the ability to form Watson–Crick base pairs, and that the presence of the metal does not influence base-pairing. Herein, we report the synthesis of Platinum(II) N-heterocyclic carbenes based on adenosine, both with a protected and unprotected ribose moiety, describing a methodology for deprotection of the sugar where both the Pt-C8 and the glycosidic bond remain stable. We also examine the cis influence of these compounds by monitoring the effect of carbene formation on the coupling constant of the Pt–P bond.

## 2. Results

### 2.1. Synthesis of Protected Adenosine Complexes

**Scheme 1:**
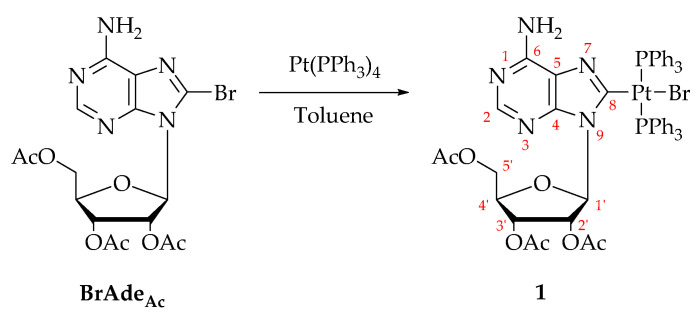
Synthesis of complex **1** by oxidative addition.

The synthesis employs oxidative addition of brominated adenosine to platinum(0). Accordingly, the reaction of proligand 2′,3′,5′-tri-*O*-acetyl-8-bromoadenosine (**BrAde_Ac_**) [8,9] with Pt(PPh_3_)_4_ in toluene, at 100 °C, affords the adenosine complex **1** (Scheme 1) in 76% isolated yield. Protection of the ribose is required, since the reaction with unprotected nucleoside leads to a mixture of products. Complex **1** was characterized by NMR spectroscopy. In the ^1^H NMR of **1**, the C’1-H of the ribose ring appears as a doublet at 6.93 ppm, shifted by 0.85 ppm with respect to the ligand precursor **BrAde_Ac_**. The most significant effect of the metalation is on the chemical shift of the NH_2_ protons, which undergo an upfield shift of 1.25 ppm with respect to **BrAde_Ac_**.

In the ^13^C{^1^H} NMR, the C8 carbon resonates at 152.1 ppm, a value similar to other platinum analogues [6], while the ribose ring of **1** gives rise to five signals between δ 62.7 and 89.0 ppm. The ^31^P{^1^H} NMR spectrum confirms the expected trans connectivity already described for similar platinum compounds [10,11]. The two phosphorous atoms are located in two non-interconverting diastereotopic sites, due to the presence of the chiral ribose unit combined with the hindered rotation around the Pt–C8 bond. For this reason, it is possible to observe the coupling between the two phosphorus nuclei, with an AB spin system at 19.29 and 18.13 ppm featuring a very substantial rooftop effect and a ^2^*J*_P,P_ = 435 Hz for both. Additionally, we observe two slightly different coupling constants with ^195^Pt, with a value of ^1^*J*_P_A_,Pt_ = 2754 Hz and ^1^*J*_P_B_,Pt_ = 2735 Hz. For ^195^Pt NMR, it is not possible to distinguish the different couplings of P_A_ and P_B_ and a triplet is observed at −4485 ppm, with a coupling constant of ^1^*J*_Pt,P_ = 2735 Hz, consistent with values determined by ^31^P{^1^H} NMR.

**Scheme 2:**
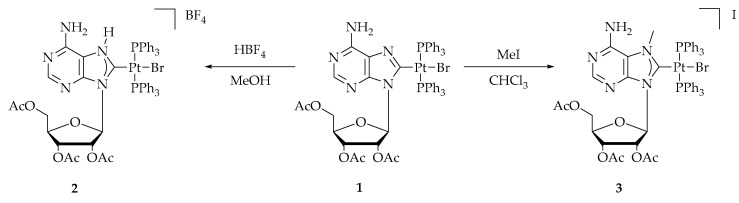
Synthesis of complexes **2** and **3** by protonation or methylation of complex **1**, respectively.

Compound **1** reacts readily at N7 in the presence of acid or methyl iodide, leading to the formation of compounds **2** and **3**, respectively (Scheme 2). This reactivity parallels the one found for palladium and platinum analogues [6,7].

Both complexes **2** and **3** were characterized by NMR spectroscopy. For complex **2,** the ^1^H NMR shows the new N7-H at 10.66 ppm, and significant variations for the NH_2_ protons, which undergo a downfield shift of 0.93 ppm, relatively to **1**. For compound **3**, the new N7-CH_3_ resonates at 3.67 ppm and, as with **2**, the NH_2_ protons undergo a downfield shift of 1.44 ppm to 6.39 ppm. In the ^13^C{^1^H}, for the protic NHC **2**, the carbenic carbon (C8) appears as a triplet at 163.6 ppm (^2^*J*_C,P_ = 9.5 Hz), with a downfield shift of 11 ppm, reflecting the formation of the NHC. This is also the case for **3**, with C8 at 163.8 ppm (^2^*J*_C,P_ = 9.0 Hz). Similarly to **1**, the ^31^P{^1^H} spectra of complexes **2** and **3** show the presence of AB spin systems due to the coupling of the two phosphorus. The AB quartet resonates at 16.15, 15.26 ppm for **2**, and at 16.12, 15.04 ppm for **3**, with ^2^*J*_P,P_ = 414 Hz and ^2^*J*_P,P_ = 413 Hz, respectively. From the ^195^Pt satellites, it is evident that in both **2** and **3**, the two phosphorus nuclei also have different coupling constants with the platinum centre, with ^1^*J*_P_A_,Pt_ = 2433 Hz; ^1^*J*_P_B_,Pt_ = 2423 Hz for **2**, and ^1^*J*_P_A_,Pt_ = 2415 Hz; ^1^*J*_P_B_,Pt_ = 2408 Hz for **3**. In the ^195^Pt NMR spectra, only one coupling constant is observed in both cases, and compounds **2** and **3** show triplets at −4498 (^1^*J*_Pt,P_ = 2421 Hz) and −4500 (^1^*J*_Pt,P_ = 2408 Hz) ppm, with ^1^*J*_Pt,P_ consistent with those determined by ^31^P NMR. This is a decrease of ^1^*J*_Pt,P_ of approximately 300 Hz relatively to **1** for both, which comes as result of carbene formation, which will be further discussed in a following section.

Crystals of compound **2** were obtained from a saturated chloroform solution and were analysed by single-crystal X-ray diffraction. Unfortunately, the X-ray analysis revealed that the crystals presented racemic twinning and thermal disorder, which prevented the refinement in the chiral space group P1, only allowing the structure refinement with full data convergence in the centrosymmetric space group P−1. Still, its molecular structure, which fully agrees with the remaining chemical analysis, is depicted in Figure S1. The platinum centre in **2** displays a square planar geometry, where the sum of the four cis-L–Pt–L’ angles is 360.3(3)°. The plane of the adenine ligand is nearly perpendicular to the coordination plane of the metal centre (dihedral angle of 92.8(3)°). All distances and angles are within the expected values for similar compounds [5]. 

**Scheme 3:**
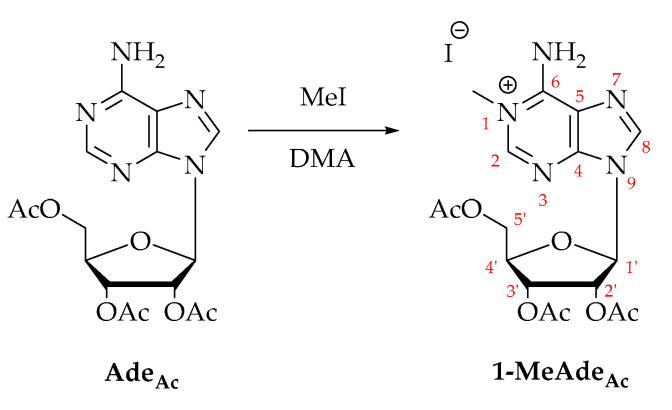
Synthesis of 1-methyl−2′,3′,5′-tri-*O*-acetyladenosinium iodide by methylation of 2′,3′,5′-tri-*O*-acetyladenosine with methyl iodide.

The formation of complexes **2** and **3** is indicative of the increased nucleophilic character of N7, which becomes the most nucleophilic position in complex **1**. This increased nucleophilic character is in line with our previous findings regarding the stability of C8-platinated guanosine derivatives. For these compounds, we have demonstrated that metalation induces a higher stability of the nucleoside towards hydrolysis [5,6,7]. This higher stability is a consequence of a stronger N9-C1′ bond, itself a result of the increased nucleophilic character of the N9 upon metalation. This is due to the electron donation from the metal centre to the adjacent nitrogen of the C-bonded heterocycle [5,6,12,13,14,15,16]. This is also the case for N7, where electron donation renders the N7 more prone to methylation and protonation. While in the case of guanosine, N7 is the most nucleophilic site, irrespectively of metalation at C8; for the adenosine fragment in **1**, the higher nucleophilic character of N7 is in striking contrast with what is observed for the free nucleoside. Indeed, the most nucleophilic position in adenosine is N1 and when adenosine is reacted with methyl iodide, 1-methyladenosine is formed exclusively (Scheme 3). We can conclude that platination at C8 induces a change in charge distribution within the adenine ring which can be further used to expand the reactivity of the nucleoside.

Importantly, for complex **1**, we were unable to determine if metalation induced a higher stability to the adenosine fragment. When adenosine and complex **1** are heated at 100 °C in DMF-*d*_7_ for several hours, both compounds remain stable and no decomposition is noted, contrasting with what we observed for guanosine complexes [5], for which the metalated nucleoside is far more stable.

### 2.2. Synthesis of Deprotected Adenosine Complexes

One of the main issues when synthesizing organometallic nucleosides is the need to protect the sugar moiety. While for protected sugars it is possible to direct the synthesis and improve efficiency, without protection, the synthesis can lead to a myriad of compounds. However, subsequent deprotection can compromise the integrity of the formed complex and lead to decomposition. Indeed, Messere et al. showed that a palladium complex C-bound thymidine decomposes upon deprotection [17]. There are nonetheless reports for the functionalization of nucleosides via cross-coupling with late transition metals that do not require protection [18], although the corresponding metalated intermediates have not yet, to the best of our knowledge, been isolated. 

**Scheme 4:**
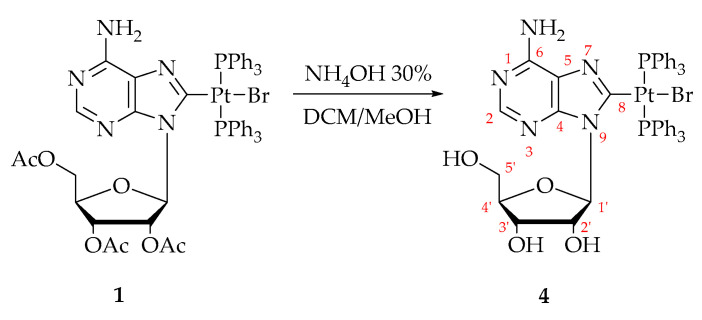
Deprotection of complex **1** with NH_4_OH.

We have recently reported a successful methodology for the deprotection of palladium and platinum compounds based on guanosine that does not compromise the stability of the M–C bond and does not promote the hydrolysis of the sugar [5,6]. This methodology makes use of acidic ethanolic solutions and can also lead to the direct formation of protic NHCs by concomitant protonation of N7. Unfortunately, when the same methodology was employed with adenosine complex **1**, deprotection was not efficient and led to a mixture of compounds. We were able to circumvent this issue by using ammonium hydroxide solutions, upon which the synthesis of compound **4** was achieved efficiently, with 66% isolated yield (Scheme 4). Again, this deprotection methodology does not compromise the Pt–C bond or the glycosidic bond, as confirmed by NMR. 

Compound **4** was characterized by NMR spectroscopy in DMSO-*d*_6_, due to the higher resolution achieved in this solvent as compared to CDCl_3_. Compounds **1** and **2**, as well as both ligand precursors, were also characterized in DMSO-*d*_6_ for comparative purposes. For compound **4**, the presence of the hydroxyl groups can be confirmed by the appearance of three additional signals 5.50(*dd*), 5.46(*d*) and 5.23(*d*) ppm. The chemical shifts of the H−2′ and H−3′ vary significantly upfield (~1.5 ppm), with respect to **1**. In the ^13^C{^1^H} NMR spectrum, the C−8 peak resonates at 147.5 ppm, similar to that of **1**. ^31^P{^1^H} shows a slightly broader singlet at 17.93 ppm with a ^1^*J*_P,Pt_ = 2820 Hz. The ^195^Pt NMR shows a triplet at −4470 ppm, with ^1^*J*_Pt,P_ ≈ 2812 Hz consistent with the value found in ^31^P{^1^H}.

**Scheme 5:**
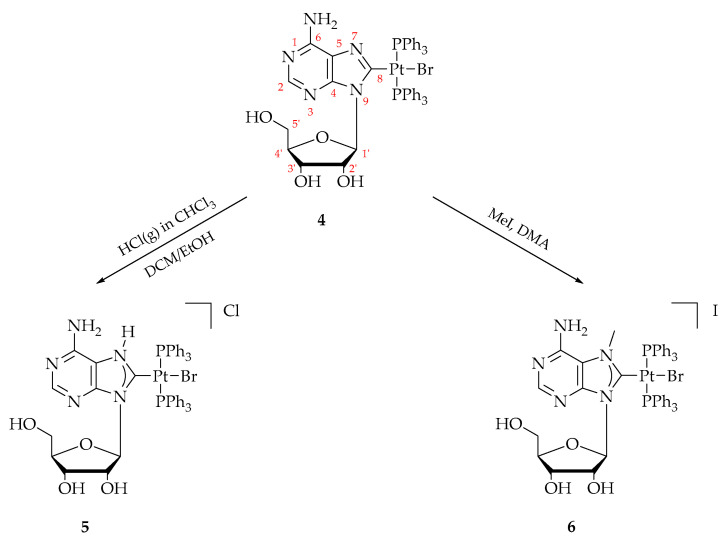
Protonation and methylation of complex **4** to obtain the corresponding NHCs.

As in the case of **1**, compound **4** reacts in the presence of a proton or methyl source leading to the formation of compounds **5** (97% yield) and **6** (70% yield), respectively (Scheme 5). Both compounds were characterized by NMR. In the ^1^H NMR, the new NH group in **5** can be detected at 14.03 ppm, a downfield shift of 1.54 ppm with respect to **2**, probably a result of hydrogen bonding with the chloride. For **6**, the new methyl group resonates as a singlet at 3.30 ppm. In both complexes, the NH_2_ group undergoes significant variations, with downfield shifts of approximately 0.9 ppm for both **5** and **6**. In the ^13^C{^1^H} NMR, the carbenic C8 in **5** resonates at 156.7 ppm as triplet with a ^2^*J*_C,P_ = 9.0 Hz, while for **6**, the triplet resonates at 161.0 ppm (^2^*J*_C,P_ = 10.0 Hz). As in the case of **2** and **3**, the variations of C8 in **5** (9 ppm) and **6** (14 ppm), with respect to **4**, reflect the formation of the NHC. In the ^31^P{^1^H} NMR, we observe an upfield shift of the ^31^P signals upon quaternization of N7. Again, an AB quartet due to the coupling between the two phosphorus nuclei is detected, at 16.79, 16.10 (^2^*J*_P,P_ = 418 Hz) for **5**, and 15.70, 15.06 (^2^*J*_P,P_ = 415 Hz) for **6**, and ^1^*J*_P,Pt_ values of 2493 Hz and 2456 Hz, respectively. In the ^195^Pt, the corresponding triplets can be detected at −4490 (**5**) and −4504 ppm (**6**), and calculated values of ^1^*J*_P,Pt_ are in agreement with those calculated for ^31^P NMR.

It should be noted that attempts to deprotect complexes **2** and **3**, where the NHC is already formed, were not successful. Thus, the synthesis of the complexes requires the deprotection of **1** and subsequent protonation/methylation. This methodology has the advantage of allowing the synthesis of complex **4**, the deprotected complex with an anionic adenosinyl ligand. For guanosine, the synthesis of the analogous compound was not possible thus far, since deprotection is performed under acidic conditions, which always lead to the formation of the corresponding protic NHC [6]. 

### 2.3. Anion Exchange

**Figure 1:**
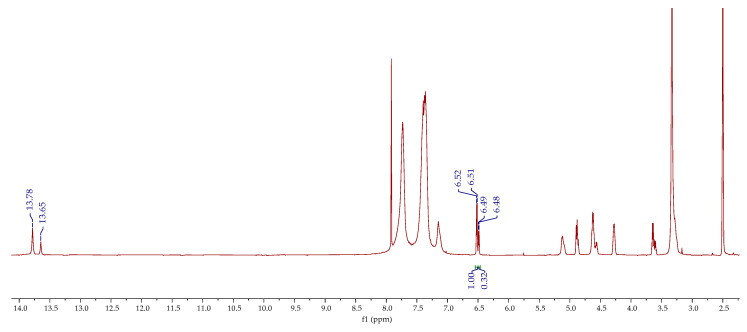
^1^H NMR spectrum of complex **5** in DMSO-*d*_6_ at room temperature, after 6 days in solution.

**Figure 2:**
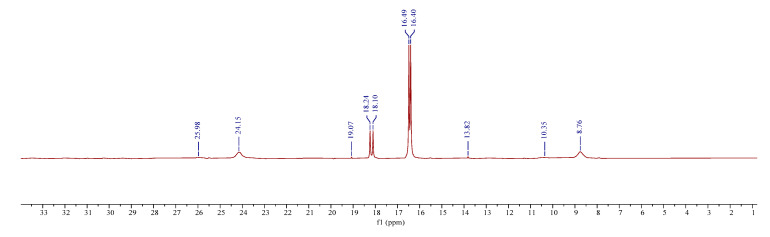
^31^P NMR spectrum of complex **5** in DMSO-*d*_6_ at room temperature, after 4 days in solution.

**Figure 3:**
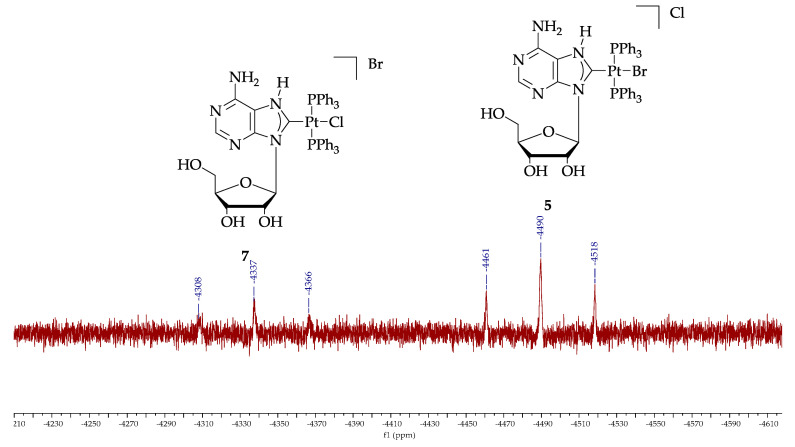
^195^Pt NMR spectrum of complex **5** in DMSO-*d*_6_ at room temperature, after 10 days in solution.

We have noted that, when in DMSO-*d*_6_ solutions, compound **5** is not stable. After a few days, the concentration of **5** decreases and another compound starts to form. An additional set of signals becomes very evident by ^1^H NMR after 6 days (Figure 1). This new compound is characterized by a new NH signal at 13.65 ppm and a doublet at 6.49 ppm, close to the H−1′ signal in **5** (6.52 ppm). The remaining ^1^H NMR signals for the sugar moiety are also indicative of the formation of the new compound in DMSO-*d*_6_ solutions, although they are mostly overlapped with those of **5**. The ^13^C{^1^H} NMR spectrum of the mixture after 6 days is not very informative, with only one clear extra signal at 94.4 ppm, close to the C−1′ signal of compound **5** (94.2 ppm). ^31^P{^1^H} NMR analysis of the solution confirms the presence of a second compound, with two singlets observed at 18.10, 18.24 ppm (Figure 2). Additionally, the ^195^Pt NMR spectrum also shows a second triplet at −4337 ppm (Figure 3), which is a shift of 153 ppm with respect to **5**.

The lability found for **5** is specific for DMSO, since solutions of **5** in CDCl_3_ (prepared in situ by addition of HCl) showed only the presence 5 and no further changes were observed, even after 2 weeks (see Supporting Information). 

**Figure 4:**
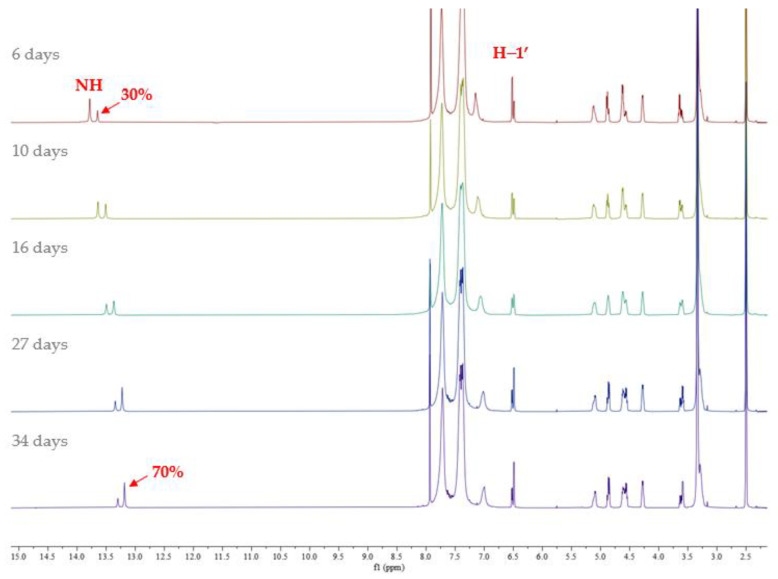
Evolution of the ^1^H NMR spectrum of complex **5** in DMSO-*d*_6_ at room temperature during different times. The mentioned percentages were calculated considering the signals for both NH and H-1′, which were in accordance.

We monitored the evolution of **5** by ^1^H NMR for several days (Figure 4). The concentration of the new compound increases very slowly, from circa 30% after 6 days to 70% after 5 weeks, after which the mixture remains essentially stable. During this time, the NH signals and the NH_2_ undergo an upfield shift for both compounds. For the remaining signals of **5**, no variation is noted, while new signals evolve with time, confirming the formation of a new compound (**7**).

**Scheme 6:**
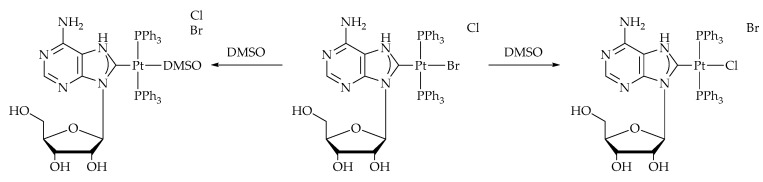
Possible co-ligand substitutions in compound **5**.

We hypothesized that this new compound could be the result of either a DMSO derivative of **5**, whereby the bromide is replaced by DMSO, or an exchange of halides between bromide and chloride, the counterion (Scheme 6).

**Figure 5:**
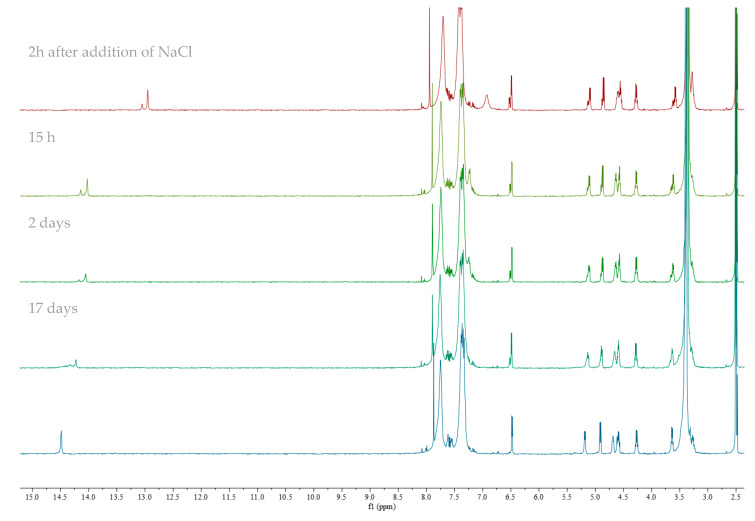
Evolution of the ^1^H NMR spectrum of complex **5** in deuterated DMSO at room temperature during different times after addition of 10 equivalents of NaCl to a 33-day-old solution.

To determine which hypothesis was correct, we prepared a solution of **5** in non-deuterated DMSO and examined this solution after 5 days by mass spectrometry (ESI-HRMS). The mass spectrum displayed a set of peaks at 1066.1608, corresponding to compound **5** (M + H), and 1022.2123, which can be attributed to a compound bearing a chloride instead of a bromide as co-ligand (M + H adduct). These values agree with the calculated theoretical values and also with the expected isotopic pattern. Additionally, to the solutions of **5** in DMSO-*d*_6_ that already had reached maximum conversion to **7** (3:7), 10 equivalents of NaCl were added. Analysis by ^1^H NMR showed that the amount of the new compound **7** increases (Figure 5), albeit very slowly, and after 17 days, it was the only compound detected. It should be noted that, in the absence of an external chloride source, the mixture remains unchanged for months. Upon the addition of NaCl, the NH signals change to lower field, probably due to hydrogen bonding. 

Compound **7** was fully characterized by NMR. Apart from the changes in the NH and H_1′_ group already highlighted, the carbenic C8 resonates at 155.2 ppm, a very slight shift with respect to **5**. The ^31^P{^1^H} NMR shows an AB quartet at 18.27, 18.14 ppm, ^2^*J*_P,P_ coupling of 420 Hz and ^195^Pt satellites with a ^1^*J*_Pt,P_ of 2530 Hz. In ^195^Pt NMR, a triplet is detected at −4340 ppm, with the coupling constant similar to that determined by ^31^P NMR. Anion exchange was also noted for DMSO solutions of compound **6**, with iodide as counterion. When dissolved in DMSO-*d*_6_, it shows the presence of two new minor species, around 6% each. The mixture remains stable and the ratio of the compounds does not increase with time nor with the addition of an iodide source. Mass spectrometry of compound **6** in DMSO solutions allowed to identify one of the compounds as the iodide adduct (**8**), but we were unable to identify the second complex. In ^195^Pt NMR, only the triplet corresponding to compound **6** is detected. ^31^P{^1^H} NMR shows two pairs of singlets at 17, 28, 17.15 (^1^*J*_Pt,P_ of 2482 Hz) and 11.47, 11.40 (^1^*J*_Pt,P_ of 2420 Hz), but we cannot confidently distinguish which one is compound **8**. Most probably, the singlets located at higher field correspond to compound **8**, being similar to other trans-[Pt(PPh_3_)_2_(NHC)I] [19]**,** but we emphasize that this assignment is purely speculative.

It should be noted that the substitutions have only been observed for the NHCs **5** and **6**. Probably, this is a consequence of the trans effect exerted by the NHC, which can accelerate [20] the dissociation of bromide to form a vacant site/solvento complex that would then lead to the formation of the halide complexes, to an extent that is most probably dependent on the stability of the final compound. The lability of compound **5** and **6** is indicative of the propensity of these complexes to undergo exchange of the ligand trans to the NHC moiety. This lability is suggestive of the capacity of adenosine complexes to coordinate to DNA and of their potential use as antiproliferative compounds which could complement the well-known activity of adenosine as an anticancer agent [21]. 

### 2.4. Comments on the ^31^P and ^195^Pt NMR

The coupling constant of the Pt–P bond can be used as an estimate of the bond strength in compounds **1**–**7**. Indeed, one bond coupling constants can be used as a measure of the bond strength when the bond involves some s character, as is the case of Pt–P [22]. Elegant work from Manassero and Pasini showed that in complexes of the type trans-[PtXY(PPh_3_)_2_], in which the mutual trans influence of the two PPh_3_ remains constant throughout, any variation on the properties of the Pt–P bond results from changes in X and Y, and thus reflect the cis influence of these ligands [23]. For these complexes, Pt(II) back donation to phosphorus is not of relevance [24], and the Pt–P bonds are formed mainly by sigma donation from P to Pt. Therefore, Pt–P bonds become weaker as the central platinum becomes less electrophilic [25]. In line with this, cis influence can be defined as the capacity of a given ligand to lower the positive charge on Pt [20,26]. For these reasons, we considered that the ^1^*J*_Pt,P_ obtained for complexes **1**–**7** should be diagnostic of the influence of the formation of the NHC and the corresponding σ-donor properties on the strength of Pt–P. Indeed, in complexes **1**–**7**, the trans effect for the phosphines remains constant. Compounds **1** and **4** are neutral, while **2**, **3**, **5**, **6** and **7** are salts, with a cationic platinum centre. The data for ^195^Pt, ^31^P and ^1^*J*_Pt,P_ is compiled in Table 1. All complexes were measured in DMSO-*d*_6_, except complex **3**, which was measured only in CDCl_3_.

When inspecting the values of ^1^*J*_Pt,P_ for complexes **1**, **2** and **3**, and **4**, **5** and **6**, it is evident that upon NHC formation, ^1^*J*_Pt,P_ decreases considerably, more than 300 Hz. This decrease is consistent in protic and methyl NHCs, both in protected and deprotected complexes. The decrease is higher for methyl NHCs **3** and **6** than for protic NHCs **2** and **5**, perhaps reflecting a slightly higher electron donation due to the methyl group [27]. This is demonstrative of the cis influence of the NHC as a consequence of its σ-donor capacity, which lowers the electrophilicity of the metal centre. Hence, we can conclude that formation of the NHC leads to a decrease in Pt–P bond strength. It is also possible to observe an upfield shift in the ^31^P signals when going from **1** to **2**/**3** and **4** to **5**/**6**, which agrees well with the reduction of electrophilic character of Pt [28,29]. As for the ^195^Pt values, an upfield shift is observed upon carbene formation, consistent with the increase of electronic density at the metal centre [30]. Although complexes bearing the NHCs are cationic, the overall charge of the cation seems to exert little effect on ^1^*J*_Pt,P_ values, being largely compensated by the increase of electron density due to the σ donation of the NHC. For compound **7**, ^1^*J*_Pt,P_ increases when changing from bromide (**6**) to chloride. It has been reported that for halogens, the cis influence decreases in the order I > Cl > F [23]. The cis influence for bromide was not computed in the aforementioned study, but the value of ^1^*J*_Pt,P_ for **7** agrees with a higher cis influence of bromide versus chloride. Although it is tempting to include in this discussion compound **8**, bearing an iodide coordinated to platinum, we cannot state, in rigor, which of the signals in ^31^P{^1^H} spectra correspond to **8**. Two minor compounds are present, one with chemical shifts of 17.28, 17.15 and ^1^*J*_Pt,P_ of 2482 Hz and the other with chemical shifts of 11.47, 11.40 and ^1^*J*_Pt,P_ of 2420 Hz. The latter values agree well with the presence of an iodide [19], and fit the trend previously reported. Still, our data is insufficient to state confidently that **8** corresponds to that specific peak. As indicated previously, we were unable to increase the concentration of **8** with the addition of an excess iodide source.

## 3. Conclusions

We have synthesized a new set of Platinum(II) complexes based on adenosine N-heterocyclic carbenes by means of C-Br oxidative addition to Pt(0)PPh_3_. A suitable deprotection methodology allowed to obtain the unprotected complexes while keeping intact the M–C and the glycosidic bonds. For adenosine complexes, platination at C8 induces a change in the charge distribution within the nucleoside and N7 becomes more nucleophilic than N1. Compound **5** and **6**, when in DMSO solutions, undergo an exchange with the corresponding counterions, reaching a maximum conversion of 70% for **5** and 7% for **6**. The formation of the NHCs from their anionic adenosinyl precursors occurs with a marked decrease in the ^1^*J*_Pt,P_, which is indicative of a decrease in Pt–P bond strength. This results from the higher cis influence of the NHC, which makes the Pt centre less positively charged, reducing its electrophilicity. Evaluation of the antiproliferative activity of these complexes is currently ongoing.

## Figures and Tables

**Table 1:** ^31^P{^1^H}and ^195^Pt NMR data for complexes **1**–**7**.

Compound	Solvent	^31^P (ppm)	^1^*J*_Pt–P_(Hz)	^195^Pt(ppm)	∆^1^*J*_Pt–P_(Hz)
**1**	CDCl_3_	19.29, 18.13	2735	−4485	-
**2**	CDCl_3_	16.15, 15.26	2421	−4498	∆ (**1**,**2**) = 314
**3**	CDCl_3_	16.12, 15.04	2408	−4500	∆ (**1**,**3**) = 327
**1**	DMSO-*d*_6_	19.05, 18.04	2786	−4488	-
**2**	DMSO-*d*_6_	17.24, 16.60	2454	−4494	∆ (**1**,**2**) ^a^ = 332
**4**	DMSO-*d*_6_	17.93	2812	−4470	-
**5**	DMSO-*d*_6_	16.79, 16.10	2494	−4490	∆ (**4**,**5**) = 326
**6**	DMSO-*d*_6_	15.70, 15.06	2456	−4504	∆ (**4**,**6**) = 364
**7**	DMSO-*d*_6_	18.62, 17.69	2530	−4340	∆ (**5**,**7**) = 74

^a^ Values for DMSO*-d*_6_.

## Data Availability

X-ray crystallographic data in CIF format are available from the Cambridge Crystallographic Data Centre (http://www.ccdc.cam.ac.uk accessed on 30 August 2021) -CCDC 2101606.

## Supplementary Materials

The synthetic procedures, NMR spectra, mass spectra and X-ray analysis are available online.

## References

[1] Jaouen: G., Vessières A., Top S. (2015). Ferrocifen Type Anti Cancer Drugs. Chem. Soc. Rev..

[2] Englinger B., Pirker C., Heffeter P., Terenzi A., Kowol C.R., Keppler B.K., Berger W. (2018). Metal Drugs and the Anticancer Immune Response. Chem. Rev..

[3] Jacobson K.A., Gao Z.G. (2006). Adenosine Receptors as Therapeutic Targets. Nat. Rev. Drug Discov..

[4] Jacobson K.A., Tosh D.K., Jain S., Gao Z.G. (2019). Historical and Current Adenosine Receptor Agonists in Preclinical and Clinical Development. Front. Cell. Neurosci..

[5] Leitão M.I.P.S., Gonzalez C., Francescato G., Filipiak Z., Petronilho A. (2020). On the Reactivity of MRNA Cap0: C-H Oxidative Addition of 7-Methylguanosine to Pt and Base Pairing Studies. Chem. Commun..

[6] Leitão M.I.P.S., Herrera F., Petronilho A. (2018). N-Heterocyclic Carbenes Derived from Guanosine: Synthesis and Evidences of Their Antiproliferative Activity. ACS Omega.

[7] Kampert F., Brackemeyer D., Tan T.T.Y., Hahn F.E. (2018). Selective C8-Metalation of Purine Nucleosides via Oxidative Addition. Organometallics.

[8] Ikehara M., Kaneko M. (1970). Studies of Nucleosides and Nucleotides-XLI. Purine Cyclonucleosides-8 Selective Sulfonylation of 8-Bromoadenosine Derivatives and an Alternate Synthesis of 8,2′- and 8,3′-S-Cyclonucleosides. Tetrahedron.

[9] Ikehara M., Yamada S. (1971). Studies of Nucleosides and Nucleotides. XLIX. Synthesis of 8-Fluoroadenosine. Chem. Pharm. Bull..

[10] Hill W.E., Minaban D.M.A., Taylor J.G., Mcauliffe C.A. (1982). Factors Influencing the Formation of Cis and Trans Isomers in Long-Chain Bis(Phosphine) Complexes of Platinum(II). J. Am. Chem. Soc..

[11] Manna J., Kuehl C.J., Whiteford J.A., Stang P.J. (1997). Preparation, via Double Oxidative Addition, and Characterization of Bimetallic Platinum and Palladium Complexes: Unique Building Blocks for Supramolecular Macrocycles. ^13^C NMR Analysis of the Nature of the Palladium-Carbon Bond. Organometallics.

[12] Alvarez E., Conejero S., Paneque M., Petronilho A., Poveda M.L., Serrano O., Carmona E. (2006). Iridium(III)-Induced Isomerization of 2-Substituted Pyridines to N-Heterocyclic Carbenes. J. Am. Chem. Soc..

[13] Álvarez E., Hernández Y.A., López-Serrano J., Maya C., Paneque M., Petronilho A., Poveda M.L., Salazar V., Vattier F., Carmona E. (2010). Metallacyclic Pyridylidene Structures from Reactions of Terminal Pyridylidenes with Alkenes and Acetylene. Angew. Chem. Int. Ed..

[14] Crociani B., di Bianca F., Giovenco A., Scrivanti A. (1983). Protonation and Methylation Reactions of 2-Pyridyl-Palladium(II) and -Platinum(II) Complexes. J. Organomet. Chem..

[15] Crociani B., Di Blanca F., Giovenco A., Berton A., Bertani R. (1989). Preparation and Reactions of 2-PyridylPlatinum(II) Complexes [PtCl(C_5_H_4_N-C_2_)(L)_2_] (L = Tertiary Phosphine) Compounds with a Markedly Nucleophilic Pyridine Nitrogen Atom. J. Organomet. Chem..

[16] Crociani B., Di Bianca F., Fontana A., Bertani R. (1992). Nucleophilic Attack by 2-Pyridylpalladium(II) and Platinum(II) Complexes on the Organic Chlorides ClCH_2_R (R = COMe, CN, Ph, Cl). J. Organomet. Chem..

[17] Romanelli A., Iacovino R., Piccialli G., Ruffo F., De Napoli L., Pedone C., Di Blasio B., Messere A. (2005). Reactions of Pd(PPh_3_)_4_ with 3′,5′-Di-*O*-Acetylthymidine: Oxidative Addition of Pd(PPh_3_)_4_ on Thymidine N3 and C4 Atoms. Organometallics.

[18] Liang Y., Wnuk S.F. (2015). Modification of Purine and Pyrimidine Nucleosides by Direct C-H Bond Activation. Molecules.

[19] Jin H., Kluth P., Hahn F.E. (2017). Synthesis of Complexes with Protic NH,NR-NHC Ligands by Oxidative Addition of N-Alkyl-2-Iodoimidazoles to [M(PPh_3_)_4_] (M = Pd, Pt) Complexes. Eur. J. Inorg. Chem..

[20] Jaganyi D., Reddy D., Gertenbach J.A., Hofmann A., Eldik R. (2004). van. Role of Chelate Substituents and Cis σ-Effect on the Rate of Ligand Substitution at Pt(N–N–N) and Pt(N–N–C) Centers. Dalt. Trans..

[21] Man S., Lu Y., Yin L., Cheng X., Ma L. (2021). Potential and Promising Anticancer Drugs from Adenosine and Its Analogs. Drug Discov. Today.

[22] Mason J. (1987). Multinuclear NMR.

[23] Rigamonti L., Manassero C., Rusconi M., Manassero M., Pasini A. (2009). Cis Influence in Trans-Pt(PPh_3_)_2_ Complexes. Dalt. Trans..

[24] Cobley C.J., Pringle P.G. (1997). Probing the Bonding of Phosphines and Phosphites to Platinum by NMR. Correlations of ^1^*J*(PtP) and Hammett Substituent Constants for Phosphites and Phosphines Coordinated to Platinum(II) and Platinum (0). Inorg. Chim. Acta.

[25] Bridgeman A.J., Gerloch M. (1995). Ligand Fields in Planar Platinum(II) Complexes. J. Chem. Soc. Dalt. Trans..

[26] Hofmann A., Dahlenburg L., Van Eldik R. (2003). Cyclometalated Analogues of Platinum Terpyridine Complexes: Kinetic Study of the Strong σ-Donor Cis and Trans Effects of Carbon in the Presence of a π-Acceptor Ligand Backbone. Inorg. Chem..

[27] Huynh H.V. (2018). Electronic Properties of N-Heterocyclic Carbenes and Their Experimental Determination. Chem. Rev..

[28] Pregosin P.S. (1986). Platinum NMR Spectroscopy. Annu. Rep. NMR Spectrosc..

[29] Still B.M., Kumar P.G.A., Aldrich-Wright J.R., Price W.S. (2007). ^195^Pt NMR-Theory and Application. Chem. Soc. Rev..

[30] Fuertes S., Chueca A.J., Martín A., Sicilia V. (2019). New NHC Cycloplatinated Compounds. Significance of the Cyclometalated Group on the Electronic and Emitting Properties of Bis-Cyanide Compounds. J. Organomet. Chem..

