# Supplementary material for: Synthesis of Platinum(II) N-Heterocyclic Carbenes Based on Adenosine
Source: Molecules. 2021 Sep 4;26(17):5384. doi: 10.3390/molecules26175384 (PMC8433631; doi:10.3390/molecules26175384)
Supplement: Supplementary file 1 [file molecules-26-05384-s001.zip › molecules-1355256-supplementary.pdf]

Supporting Information

Synthesis of Platinum(II) N-Heterocyclic Carbenes based on Adenosine

Maria Inês P.S. Leitão[1], Giulia Francescato[1], Clara S. B. Gomes[2,3,4] and Ana Petronilho[1*]

[1] ITQB – Instituto de Tecnologia Química e Biológica, Universidade Nova de Lisboa, Estação Agronómica Nacional, Oeiras, Portugal.

[2] LAQV-REQUIMTE, Department of Chemistry, NOVA School of Science and Technology, Universidade Nova de Lisboa, 2829-516 Caparica, Portugal.

[3] Associate Laboratory i4HB - Institute for Health and Bioeconomy, School of Science and Technology, NOVA University Lisbon, 2819-516 Caparica, Portugal

[4] UCIBIO – Applied Molecular Biosciences Unit, Department of Chemistry, School of Science and Technology, NOVA University Lisbon, 2819-516 Caparica, Portugal

* Correspondence: ana.petronilho@itqb.unl.pt; Tel.: +351-214-469-716

Table of contents

1. Synthetic Procedures 3
1.1. General considerations 3
1.2. Adenosine 3
1.3. 8-Bromoadenosine 4
1.4. 2′,3′,5′-Tri-*O*-acetyl-8-bromoadenosine 4
1.5. Complex **1** 5
1.6. Complex **2** 6
1.7. Complex **3** 8
1.8. Complex **4** 9
1.9. Complex **5** 10
1.10. Complex **6** 11
1.11. Complex **7** 12
2. NMR and MS spectra 13
2.1. NMR spectra 13
2.2. HRMS spectra 30
3. Crystallographic details for complex **2** 38
4. Measurement of the stability of **Ade$_{Ac}$** by ^{1}H NMR 40
5. Measurement of the stability of **1** by ^{1}H NMR 41
6. Measurement of the stability of **5** in DMSO-d$_6$ by ^{1}H NMR 43
7. References 45

1. Synthetic Procedures

1.1. General considerations

The syntheses of complexes were carried out under an inert atmosphere of N_2 using Schlenk techniques. All ^{1}H and $^{13}C\{^{1}H\}$ NMR spectra were recorded at room temperature on Bruker spectrometers (400 MHz). Chemical shifts are reported as δ-values in ppm relative to the deuterated solvent peaks: DMSO-d_6 (δH: 2.50; δC: 39.52) and chloroform-*d* (δH: 7.26; δC: 77.16). 8-bromoadenosine[1] and 2',3',5′-Tri-*O*-acetyl-8-bromoadenosine[2] were synthesized according to reported procedures. Mass Spectroscopy measurements were obtained by the UniMass Laboratory at Instituto de Tecnologia Química e Biológica, Portugal.

1.2. Adenosine

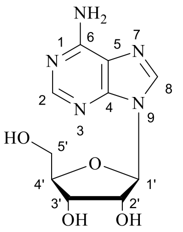

^{1}H NMR (400 MHz, DMSO-d_6): δ 8.36 (s, 1H, H-8), 8.16 (s, 1H, H-2), 7.38 (br s, 2H, NH_2), 5.90 (d, 1H, H-1′, $^{3}J_{H\text{-}1', H\text{-}2'}$ ≈ 6.3 Hz), 5.47 (d, 1H, OH-2′, $^{3}J_{OH\text{-}2', H\text{-}2}$ ≈ 6.3 Hz), 5.45 (dd, 1H, OH-5′, $^{3}J_{OH\text{-}5', H\text{-}5'a}$ ≈ 4.0 Hz, $^{3}J_{OH\text{-}5', H\text{-}5'b}$ ≈ 7.2 Hz), 5.21 (d, 1H, 3′-OH, $^{3}J_{OH\text{-}3', H\text{-}3'}$ ≈ 4.8 Hz), 4.63 (ddd, 1H, H-2′, $^{3}J_{H\text{-}2', H\text{-}3'}$ ≈ 4.8 Hz, $^{3}J_{H\text{-}2', H\text{-}1'}$ ≈ $^{3}J_{H\text{-}2', OH\text{-}2'}$ ≈ 6.3 Hz), 4.16 (ddd, 1H, H-3′, $^{3}J_{H\text{-}3',H\text{-}2'}$ ≈ $^{3}J_{H\text{-}3', OH\text{-}3'}$ ≈ 4.8 Hz, $^{3}J_{H\text{-}3', H\text{-}4'}$ ≈ 6.8 Hz), 3.98 (ddd, 1H, H-4′, $^{3}J_{H\text{-}4',H\text{-}5'a}$ ≈ $^{3}J_{H\text{-}4', H\text{-}5'b}$ ≈ 4.0 Hz, $^{3}J_{H\text{-}4', H\text{-}3'}$ ≈ 6.8 Hz), 3.69 (dt, 1H, H-5′a, $^{3}J_{H\text{-}5'a,OH\text{-}5'}$ ≈ $^{3}J_{H\text{-}5'a, H\text{-}4'}$ ≈ 4.0 Hz, $^{3}J_{H\text{-}5'a, H\text{-}5'b}$ ≈ 12.0 Hz), 3.57 (ddd, 1H, H-5′b, $^{3}J_{H\text{-}5'b, H\text{-}4'}$ ≈ 4.0 Hz, $^{3}J_{H\text{-}5'b,OH\text{-}5'}$ ≈ 7.2 Hz, $^{3}J_{H\text{-}5'b, H\text{-}5'a}$ ≈ 12.0 Hz) ppm.

$^{13}C\{^{1}H\}$ NMR (100 MHz, DMSO-d_6): δ 156.2 (C-6), 152.5 (C-2), 149.1 (C-4), 140.1 (C-8), 119.5 (C-5), 88.0 (C-1′), 86.0 (C-4′), 73.5 (C-2′), 70.8 (C-3′), 61.8 (C-5′) ppm.

1.3. 8-Bromoadenosine

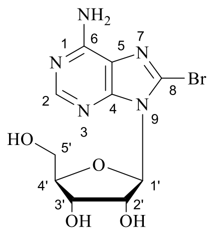

^{1}H NMR (400 MHz, DMSO-d_6): δ 8.12 (s, 1H, H-2), 7.57 (br s, 2H, NH_2), 5.83 (d, 1H, H-1′, $^{3}J_{H-1′, H-2′}$ ≈ 7.0 Hz), 5.50 (dd, 1H, OH-5′, $^{3}J_{OH-5′, H-5′a}$ ≈ 4.0 Hz, $^{3}J_{OH-5′, H-5′b}$ ≈ 8.4 Hz), 5.46 (d, 1H, OH-2′, $^{3}J_{OH-2′, H-2}$ ≈ 6.3 Hz), 5.23 (d, 1H, 3′-OH, $^{3}J_{OH-3′, H-3′}$ ≈ 4.4 Hz), 5.09 (ddd, 1H, H-2′, $^{3}J_{H-2′, H-3′}$ ≈ 4.8 Hz, $^{3}J_{H-2′, H-1′}$ ≈ $^{3}J_{H-2′, OH-2′}$ ≈ 6.5 Hz), 4.16 (ddd, 1H, H-3′, $^{3}J_{H-3′,H-2′}$ ≈ $^{3}J_{H-3′, OH-3′}$ ≈ 4.8 Hz, $^{3}J_{H-3′, H-4′}$ ≈ 2.4 Hz), 3.98 (ddd, 1H, H-4′, $^{3}J_{H-4′, H-3′}$ ≈ 2.4 Hz, $^{3}J_{H-4′,H-5′a}$ ≈ $^{3}J_{H-4′, H-5′b}$ ≈ 4.0 Hz), 3.68 (dt, 1H, H-5′a, $^{3}J_{H-5′a,OH-5′}$ ≈ $^{3}J_{H-5′a, H-4′}$ ≈ 4.0 Hz, $^{3}J_{H-5′a, H-5′b}$ ≈ 12.0 Hz), 3.52 (ddd, 1H, H-5′b, $^{3}J_{H-5′b, H-4′}$ ≈ 4.0 Hz, $^{3}J_{H-5′b,OH-5′}$ ≈ 8.4 Hz, $^{3}J_{H-5′b, H-5′a}$ ≈ 12.0 Hz) ppm.

^{13}C{^{1}H} NMR (100 MHz, DMSO-d_6): δ 155.2 (C-6), 152.5 (C-2), 149.9 (C-4), 127.2 (C-8), 119.7 (C-5), 90.4 (C-1′), 86.7 (C-4′), 71.1 (C-2′), 70.9 (C-3′), 62.1 (C-5′) ppm.

1.4. 2′,3′,5′-Tri-*O*-acetyl-8-bromoadenosine

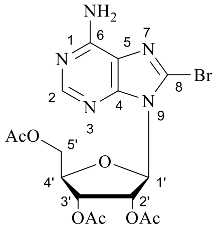

^{1}H NMR (400 MHz, DMSO-d_6): δ 8.17 (s, 1H, H-2), 7.56 (br s, 2H, NH_2), 6.28 (dd, 1H, H-2′, $^{3}J_{H-2′, H-1′}$ ≈ 4.4 Hz, $^{3}J_{H-2′, H-3′}$ ≈ 6.4 Hz), 6.05 (d, 1H, H-1′, $^{3}J_{H-1′, H-2′}$ ≈ 4.4 Hz), 5.77 (dd, 1H, H-3′, $^{3}J_{H-3′,H-2′}$ ≈ 6.4 Hz, $^{3}J_{H-3′, H-4′}$ ≈ 6.0 Hz), 4.42 (dt, 1H, H-5′a, $^{3}J_{H-5′a, H-4′}$ ≈ 3.6 Hz, $^{3}J_{H-5′a, H-5′b}$ ≈ 11.6 Hz), 4.40-4.36 (m, 1H, H-4′), 4.18 (dd, 1H, H-5′b, $^{3}J_{H-5′b, H-4′}$ ≈ 4.8 Hz, $^{3}J_{H-5′b, H-5′a}$ ≈ 11.6 Hz), 2.11 (s, 3H, 3′-CH_3CO), 2.07 (s, 3H, 2′-CH_3CO), 1.93 (s, 3H, 5′-CH_3CO) ppm.

^{13}C{^{1}H} NMR (100 MHz, DMSO-d_6): δ 170.0 (5′-CH_3CO), 169.5, 169.5 (2′+3′-CH_3CO), 155.1 (C-6), 153.1 (C-2), 150.2 (C-4), 126.5 (C-8), 119.3 (C-5), 87.9 (C-1′), 79.3 (C-4′), 71.2 (C-2′), 69.6 (C-3′), 62.3 (C-5′), 20.4 (3′+5′-CH_3CO), 20.3 (2′-CH_3CO) ppm.

1.5. Complex 1

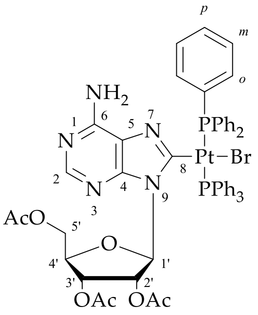

2',3',5′-Tri-*O*-acetyl-8-bromoadenosine (72 mg, 0.15 mmol) and $Pt(PPh_3)_4$ (190 mg, 0.15 mmol) were suspended in degassed toluene (9 mL) and stirred at 100 °C for 16h. After cooling, the precipitate was filtered and washed with Et_2O (20 mL). To the filtrate, pentane is added to form a precipitate, which was then filtered off and washed profusely with Et_2O and pentane to remove the toluene. Both solids were dried under vacuum to yield **1** (138 mg, 76%) as a white solid.

^{1}H NMR (400 MHz, $CDCl_3$): δ 7.88 (s, 1H, H-2), 7.75-7.56 (m, 12H, PPh_3, H_o), 7.36-7.15 (m, 20H, PPh_3, H_m+H_p), 6.93 (d, 1H, H-1′, $^{3}J_{H\text{-}1', H\text{-}2'}$ ≈ 3.6 Hz), 5.99 (dd, H-3′, 1H, $^{3}J_{H\text{-}3', H\text{-}2'}$ ≈ 6.0 Hz, $^{3}J_{H\text{-}3', H\text{-}4'}$ ≈ 6.4 Hz), 5.84 (dd, 1H, H-2′, $^{3}J_{H\text{-}2', H\text{-}1'}$ ≈ 3.6 Hz, $^{3}J_{H\text{-}2', H\text{-}3'}$ ≈ 6.0 Hz), 4.95 (br s, 2H, NH_2), 3.96-3.88 (m, 2H, H-5′), 3.82-3.70 (m, 1H, H-4′), 2.08 (s, 3H, 7-NCH_3), 2.15, 1.74 (s, 3H each, 2′+3′-CH_3CO), 1.89 (s, 3H, 5′-CH_3CO) ppm.

$^{13}C\{^{1}H\}$ NMR (100 MHz, $CDCl_3$): δ 170.6 (5′-CH_3CO), 169.2, 169.1 (2′&3′-CH_3CO), 152.1 (C-8), 151.9 (C-6), 151.2 (C-4), 149.4 (C-2), 134.9, 134.7 (2×$P(C_6H_5)_3$, CH_o), 130.8, 130.6 (2×$P(C_6H_5)_3$, CH_m & CH_p), 130.2-129.4 (2×$P(C_6H_5)_3$, C_{quat}), 128.2, 127.9 (2×$P(C_6H_5)_3$, CH_m & CH_p), 123.7 (C-5), 89.0 (C-1′), 78.3 (C-4′), 72.9 (C-2′), 70.1 (C-3′), 62.7 (C-5′), 20.8, 20.7, 20.4 (2′ & 3′ & 5′-CH_3CO) ppm.

$^{31}P\{^{1}H\}$ NMR (162 MHz, $CDCl_3$): δ = 19.29 (d, $^{2}J_{PP}$ = 435 Hz, P_A), 18.13 (d, $^{2}J_{PP}$ = 435 Hz, P_B) ppm. Pt satellites $^{1}J_{PA, Pt}$ = 2754 Hz, $^{1}J_{PB, Pt}$ = 2735 Hz.

^{195}Pt NMR (86 MHz, $CDCl_3$): δ = -4485 (t, $^{1}J_{Pt, P}$ ≈ 2735 Hz).

^{1}H NMR (400 MHz, DMSO-d_6): δ 7.62 (s, 1H, H-2), 7.65-7.23 (m, 30H, 2×PPh_3), 6.85 (d, 1H, H-1′, $^{3}J_{H\text{-}1', H\text{-}2'}$ ≈ 3.2 Hz), 6.30 (br s, 2H, NH_2), 5.77 (dd, H-3′, 1H, $^{3}J_{H\text{-}3', H\text{-}2'}$ ≈ 6.0 Hz, $^{3}J_{H\text{-}3', H\text{-}4'}$ ≈ 6.8 Hz), 5.41 (dd, 1H, H-2′, $^{3}J_{H\text{-}2', H\text{-}1'}$ ≈ 3.2 Hz, $^{3}J_{H\text{-}2', H\text{-}3'}$ ≈ 6.0 Hz), 3.89 (dd, 1H, H-5′a, $^{3}J_{H\text{-}5'a, H\text{-}4'}$ ≈ 2.8 Hz, $^{2}J_{H\text{-}}$

$_{5'a, H\text{-}5'b}$ ≈ 11.2 Hz), 3.81-3.69 (m, 2H, H-4′+H-5′b), 2.02, 1.81 (s, 3H each, 2′+3′-C*H*$_3$CO), 1.80 (s, 3H, 5′-C*H*$_3$CO), ppm.

^{13}C{^{1}H} NMR (100 MHz, DMSO-d$_6$): δ 169.8 (5′-CH$_3$CO), 168.8 (2′+3′-CH$_3$CO), 152.9 (C-6), 150.3 (C-4), 148.7 (C-2), 147.4 (C-8), 134.5, 134.1, 130.8, 130.4 (2×P(C$_6$H$_5$)$_3$, *C*H), 129.9-128.8 (2×P(C$_6$H$_5$)$_3$, *C*$_{quat}$), 128.0, 127.6 (P(C$_6$H$_5$)$_3$, *C*H), 123.5 (C-5), 88.5 (C-1′), 77.4 (C-4′), 72.4 (C-2′), 68.9 (C-3′), 62.6 (C-5′), 20.3, 20.0 (2′+3′+5′-*C*H$_3$CO) ppm.

^{31}P{^{1}H} NMR (162 MHz, DMSO-d$_6$): δ = 19.05 (d, $^2J_{PP}$ = 437 Hz, P$_A$), 18.14 (d, $^2J_{PP}$ = 437 Hz, P$_B$) ppm. Pt satellites $^1J_{P, Pt}$ = 2786 Hz.

^{195}Pt NMR (86 MHz, DMSO-d$_6$): δ = -4488 (t, $^1J_{Pt, P}$ = 2786 Hz).

HRMS (ESI) for $C_{52}H_{49}BrN_5O_7P_2Pt$ [M+H] calcd. 1191.1933. Found: 1191.1939

1.6. Complex 2

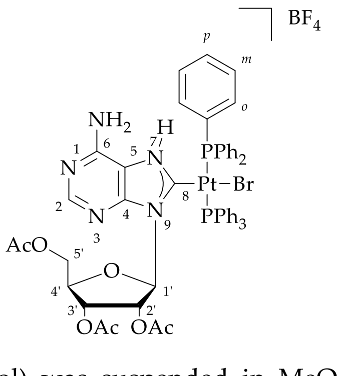

Complex **1** (100 mg, 0.084 mmol) was suspended in MeOH (4 mL) and stirred at room temperature for 5 minutes. 200 μL of HBF4 (48% in water) were added to the suspension, upon which complete solubilisation of the solid was observed. Within a few seconds a white precipitate forms and the suspension was kept stirring at room temperature for 30 minutes. The precipitate was filtered-off, washed with Et2O (3×5 mL) and dried under air to afford the complex **2** as a white powder (86 mg, 80%). The compound can be recrystallized by slow evaporation of a saturated chloroform solution.

^{1}H NMR (400 MHz, CDCl$_3$): δ 10.66 (br s, 1H, 7-NH), 8.10 (s, 1H, H-2), 8.0-7.52 (m, 12H, PPh$_3$, H$_o$), 7.50-7.08 (m, 18H, PPh$_3$, H$_m$ & H$_p$), 6.85 (d, 1H, H-1′, $^3J_{H\text{-}1', H\text{-}2'}$ ≈ 5.2 Hz), 6.44 (dd, H-2′, 1H,

$^3J_{H\text{-}2', H\text{-}1'}$ ≈ 5.2 Hz, $^3J_{H\text{-}2', H\text{-}3'}$ ≈ 5.5 Hz), 5.88 (br s, 2H, NH_2), 5.77 (dd, 1H, H-3′, $^3J_{H\text{-}3', H\text{-}2'}$ ≈ 5.5 Hz, $^3J_{H\text{-}3', H\text{-}4'}$ ≈ 5.8 Hz), 4.03-3.89 (m, 2H, H-5′), 3.85-3.73 (m, 1H, H-4′), 2.16 (s, 3H, 2′-CH_3CO), 1.94 (s, 3H, 5′-CH_3CO), 1.54 (s, 3H, 3′-CH_3CO) ppm.

$^{13}C\{^1H\}$ NMR (100 MHz, $CDCl_3$): δ 170.2 (5′-CH_3CO), 169.2 (3′-CH_3CO), 168.8 (2′-CH_3CO), 163.6 (t, C-8, $^2J_{C,P}$ = 9.5 Hz), 152.7 (C-2), 149.0 (C-6), 148.3 (C-4), 134.6 (2×P(C_6H_5)$_3$, CH_o), 131.4, 131.2, 128.7, 128.4 (2×P(C_6H_5)$_3$, CH_m & CH_p), 128.3-127.3 (2×P(C_6H_5)$_3$, C_{quat}), 111.3 (C-5), 90.6 (C-1′), 80.0 (C-4′), 71.0 (C-2′), 69.8 (C-3′), 62.0 (C-5′), 20.7 (5′-CH_3CO), 20.6 (3′-CH_3CO), 19.9 (2′-CH_3CO) ppm.

$^{31}P\{^1H\}$ NMR (162 MHz, $CDCl_3$): δ = 16.15 (d, $^2J_{PP}$ = 414 Hz, P_A), 15.26 (d, $^2J_{PP}$ = 414 Hz, P_B) ppm. Pt satellites $^1J_{PA, Pt}$ = 2433 Hz, $^1J_{PB, Pt}$ = 2423 Hz.

^{195}Pt NMR (86 MHz, $CDCl_3$): δ = -4498 (t, $^1J_{Pt, P}$ ≈ 2421 Hz).

^{1}H NMR (400 MHz, DMSO-d$_6$): δ 12.49 (br s, 1H, 7-NH), 8.08 (s, 1H, H-2), 8.19-6.65 (m, 32H, 2×PPh_3 + NH_2), 6.72 (d, 1H, H-1′, $^3J_{H\text{-}1', H\text{-}2'}$ ≈ 5.2 Hz), 6.19 (dd, H-2′, 1H, $^3J_{H\text{-}2', H\text{-}1'}$ ≈ 5.2 Hz, $^3J_{H\text{-}2', H\text{-}3'}$ ≈ 6.0 Hz), 5.59 (dd, 1H, H-3′, $^3J_{H\text{-}3', H\text{-}2'}$ ≈ 6.0 Hz, $^3J_{H\text{-}3', H\text{-}4'}$ ≈ 5.2 Hz), 4.02-3.84 (m, 2H, H-5′), 3.84-3.75 (m, 1H, H-4′), 2.17 (s, 3H, 3′-CH_3CO), 1.87 (s, 3H, 5′-CH_3CO), 1.61 (s, 3H, 2′-CH_3CO) ppm.

$^{13}C\{^1H\}$ NMR (100 MHz, DMSO-d$_6$): δ 169.7 (5′-CH_3CO), 169.2 (3′-CH_3CO), 168.7 (2′-CH_3CO), 158.6 (t, C-8, $^2J_{C,P}$ = 9.0 Hz), 153.2 (C-2), 149.7 (C-6), 147.8 (C-4), 136.8-126.2 (3×P(C_6H_5)$_3$), 111.5 (C-5), 89.9 (C-1′), 79.6 (C-4′), 70.3 (C-2′), 68.8 (C-3′), 61.6 (C-5′), 20.4, 20.4 (3′+5′-CH_3CO), 19.6 (2′-CH_3CO) ppm.

$^{31}P\{^1H\}$ NMR (162 MHz, DMSO-d_6): δ = 17.24 (d, $^2J_{PP}$ = 415 Hz, P_A), 16.60 (d, $^2J_{PP}$ = 415 Hz, P_B) ppm. Pt satellites $^1J_{P, Pt}$ = 2454 Hz.

^{195}Pt NMR (86 MHz, DMSO-d$_6$): δ = -4494 (t, $^1J_{Pt, P}$ = 2454 Hz).

HRMS (ESI) for $C_{52}H_{50}BrN_5O_7P_2Pt$ [M+H] calcd. 1192.2011. Found: 1192.1944

1.7. Complex 3

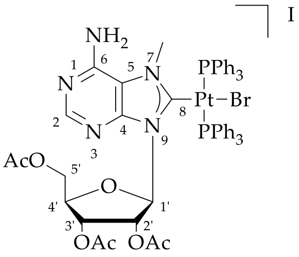

Complex **1** (100 mg, 0.084 mmol) was dissolved in chloroform (2.6 mL) and methyl iodide (16 μL, 0.251 mmol) was added. The resulting solution was stirred at room temperature for 2 days. The volatiles were removed under vacuum to give complex **3** as a yellowish solid (104 mg, 92%).

^{1}H NMR (400 MHz, $CDCl_3$): δ 8.44-6.67 (m, 30H, PPh_3), 8.13 (s, 1H, H-2), 6.92 (d, 1H, H-1′, $^{3}J_{H\text{-}1', H\text{-}2'}$ ≈ 4.8 Hz), 6.49 (dd, 1H, H-2′, $^{3}J_{H\text{-}2', H\text{-}1'}$ ≈ 4.8 Hz, $^{3}J_{H\text{-}2', H\text{-}3'}$ ≈ 5.6 Hz), 6.39 (br s, 2H, NH_2), 5.79 (dd, H-3′, 1H, $^{3}J_{H\text{-}3', H\text{-}2'}$ ≈ $^{3}J_{H\text{-}3', H\text{-}4'}$ ≈ 5.6 Hz), 3.97 (dd, 1H, H-5′a, $^{3}J_{H\text{-}5'a, H\text{-}4'}$ ≈ 4.8 Hz, $^{2}J_{H\text{-}5'a, H\text{-}5'b}$ ≈ 12.4 Hz), 3.84 (dd, 1H, H-5′b, $^{3}J_{H\text{-}5'b, H\text{-}4'}$ ≈ 3.6 Hz, $^{2}J_{H\text{-}5'b, H\text{-}5'a}$ ≈ 12.4 Hz), 3.67 (s, 3H, 7-N*C*H_3), 3.64-3.80 (m, 1H, H-4′), 2.16 (s, 3H, 2′-CH_3CO), 1.95 (s, 3H, 5′-CH_3CO), 1.60 (s, 3H, 3′-CH_3CO).

^{13}C{^{1}H} NMR (100 MHz, $CDCl_3$): δ 170.1 (5′-CH_3*C*O), 169.1 (3′-CH_3*C*O), 168.7 (2′-CH_3*C*O), 163.8 (t, C-8 $^{2}J_{C,P}$ = 9.0 Hz), 153.9 (C-2), 150.0 (C-6), 149.4 (C-4), 138.2-125.7 (2×P(C_6H_5)$_3$), 112.0 (C-5), 90.2 (C-1′), 80.0 (C-4′), 71.1 (C-2′), 69.6 (C-3′), 61.4 (C-5′), 40.4 (7-N*C*H_3), 20.8 (5′-*C*H_3CO), 20.7 (2′-*C*H_3CO), 20.1 (3′-*C*H_3CO) ppm.

^{31}P{^{1}H} NMR (162 MHz, $CDCl_3$): δ = 16.12 (d, $^{2}J_{PP}$ = 413 Hz, P_A), 15.04 (d, $^{2}J_{PP}$ = 413 Hz, P_B) ppm. Pt satellites $^{1}J_{PA, Pt}$ = 2415 Hz, $^{1}J_{PB, Pt}$ = 2408 Hz.

^{195}Pt NMR (86 MHz, $CDCl_3$): δ = -4500 (t, $^{1}J_{Pt, P}$ ≈ 2408 Hz).

HRMS (ESI) for $C_{53}H_{51}BrN_5O_7P_2Pt$ [M+H] calcd. 1206.2168. Found: 1206.2133.

1.8. Complex 4

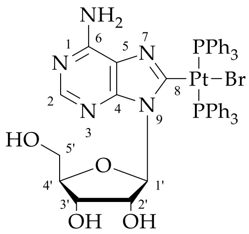

Complex **1** (198 mg, 0.16 mmol) was dissolved in a mixture of dichloromethane (6 mL) and methanol (6 ml), then NH_4OH 30% (4.1 ml) was added. The resulting solution was stirred at room temperature for 24 hours. The volatiles were removed under vacuum and the solid recrystallized in methanol to give complex **4** as a white solid (112 mg, 66%).

^{1}H NMR (400 MHz, DMSO): δ 7.74-7.18 (m, 30H, PPh_3), 7.53 (s, 1H, H-2), 6.70 (d, 1H, H-1′, $^3J_{H-1', H-2'}$ ≈ 4.4 Hz), 6.27 (br s, 2H, NH_2), 5.03 (d, 1H, OH-3′, $^3J_{OH-3', H-3'}$ ≈ 5.6 Hz), 4.84 (d, OH-2′, 1H, $^3J_{OH-2', H-2'}$ ≈ 5.6 Hz), 4.38-4.26 (m, 2H, H-3′ & OH-5′), 4.01-3.82 (m, 1H, H-2′), 3.68-3.55 (m, 1H, H-4′), 3.41-3.25* (m, 2H, H-5′). *Determined by HSQC

^{13}C{^{1}H} NMR (100 MHz, DMSO): δ 152.8 (C-6), 150.2 (C-4), 147.9 (C-2), 147.5 (C-8), 134.5, 130.4, 130.2, 130.1-128.9, 127.7 (2×$P(C_6H_5)_3$), 123.7 (C-5), 92.2 (C-1′), 83.7 (C-4′), 72.3 (C-2′), 70.1 (C-3′), 61.9 (C-5′) ppm.

^{31}P{^{1}H} NMR (162 MHz, DMSO): δ = 17.93 (s) ppm. Pt satellites $^1J_{P, Pt}$ = 2820 Hz.

^{195}Pt NMR (86 MHz, DMSO-d_6): δ = -4470 (t, $^1J_{Pt, P}$ ≈ 2812 Hz).

HRMS (ESI) for $C_{46}H_{44}BrN_5O_7P_2Pt$ [M+H2] calcd. 1066.1694. Found: 1066.1630.

1.9. Complex 5

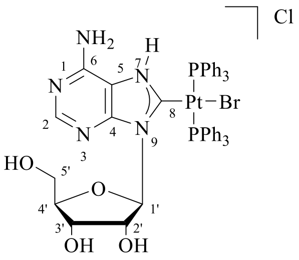

Complex **4** (50 mg, 0.047 mmol) was dissolved in dichloromethane (1.1 mL) and ethanol was added (11 mL). To the resulting suspension was added a saturated solution of HCl (g) in chloroform and the reaction mixture was stirred at room temperature for 15 minutes. The volatiles were removed under vacuum to give complex **5** as a white solid (50 mg, 97%).

^{1}H NMR (400 MHz, DMSO-d_6): δ 14.03 (s, 1H, 7-NH), 8.08-7.05 (m, 30H, PPh_3), 7.91 (s, 1H, H-2), 7.21 (br s, 2H, NH_2), 6.52 (d, 1H, H-1′, $^3J_{H\text{-}1',H\text{-}2'}$ ≈ 4.4 Hz), 5.21-5.02 (m, 1H, OH), 4.94-4.83 (m, 1H, OH), 4.74-4.49 (m, 2H, OH & H-2′), 4.28 (ddd, 1H, H-3′, $^3J_{H\text{-}3',H\text{-}2'}$ ≈ $^3J_{H\text{-}3',H\text{-}4'}$ ≈ $^3J_{H\text{-}3',OH\text{-}3'}$ ≈ 5.0 Hz), 3.65 (ddd, 1H, H-4′, $^3J_{H\text{-}4',H\text{-}3'}$ ≈ 4.4 Hz, $^3J_{H\text{-}4',H\text{-}5'a}$ ≈ $^2J_{H\text{-}4',H\text{-}5'b}$ ≈ 5.0 Hz), 3.35-3.24* (m, 2H, H-5′). *Determined by HSQC

^{13}C{^{1}H} NMR (100 MHz, DMSO-d_6): δ 156.7 (t, C-8 $^2J_{C,P}$ = 9.0 Hz), 152.4 (C-2), 149.9 (C-6), 147.4 (C-4), 136-127 (2×$P(C_6H_5)_3$), 111.7 (C-5), 94.2 (C-1′), 84.7 (C-4′), 71.3, 69.9 (C-2′ & C-3′), 61.2 (C-5′) ppm.

^{31}P{^{1}H} NMR (162 MHz, DMSO-d_6): δ = 16.79 (d, $^2J_{PP}$ = 418 Hz, P_A), 16.10 (d, $^2J_{PP}$ = 418 Hz, P_B) ppm. Pt satellites $^1J_{P,Pt}$ = 2493 Hz.

^{195}Pt NMR (86 MHz, DMSO-d_6): δ = -4490 (t, $^1J_{Pt,P}$ = 2494 Hz).

HRMS (ESI) for $C_{46}H_{44}BrN_5O_4P_2Pt$ [M+H] calcd. 1066.1694. Found: 1066.1608.

1.10. Complex 6

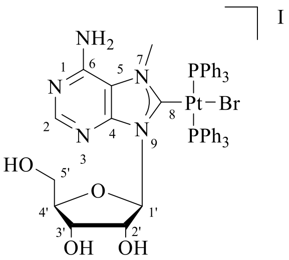

Complex **4** (50 mg, 0.047 mmol) was dissolved in dimethylacetamide (240 μL) and methyl iodide (14 μL, 0.23 mmol) was added. The resulting solution was stirred at room temperature overnight, after which Et_2O is added. A precipitate is formed, which was then filtered and washed profusely with Et_2O and pentane to remove the dimethylacetamide. The resulting solid was subsequently dried under vacuum to yield **6** as a white solid (38 mg, 70%).

^1H NMR (400 MHz, DMSO-d_6): δ 8.24-6.49 (m, 30H, PPh_3), 8.05 (s, 1H, H-2), 7.12 (br s, 2H, NH_2), 6.65 (d, 1H, H-1′, $^3J_{H-1', H-2'}$ ≈ 5.2 Hz), 5.15 (d, 1H, OH-3′, $^3J_{OH-3', H-3'}$ ≈ 5.2 Hz), 4.87 (ddd, 1H, H-2′, $^3J_{H-2', H-1'}$ ≈ $^3J_{H-2', H-3'}$ ≈ 5.2 Hz, $^3J_{H-2', OH-2'}$ ≈ 6.8 Hz,), 4.56 (dd, 1H, 5′-OH, $^3J_{OH-5', H-5a}$ ≈ $^3J_{OH-5', H-5b}$ ≈ 5.6 Hz), 4.40 (d, 1H, 2′-OH, $^3J_{OH-2', H-2'}$ ≈ 6.8 Hz), 4.24 (ddd, 1H, H-3′, $^3J_{H-3',H-2'}$ ≈ $^3J_{H-3', OH-3'}$ ≈ $^3J_{H-3', H-4'}$ ≈ 5.2 Hz), 3.39-3.30* (m, 1H, H-4′), 3.30 (s, 3H, 7-CH_3), 3.25-3.16 (m, 2H, H-5′) ppm. *Determined by HSQC

$^{13}C\{^1H\}$ NMR (100 MHz, DMSO-d_6): δ 161.0 (t, C-8 $^2J_{C,P}$ = 9.0 Hz), 153.1 (C-2), 149.9 (C-6), 149.2 (C-4) 137.0-126.3 (2×$P(C_6H_5)_3$), 111.6 (C-5), 93.5 (C-1′), 84.7 (C-4′), 70.5 (C-2′), 69.5 (C-3′), 60.5 (C-5′), 37.5 (7-NCH_3) ppm.

$^{31}P\{^1H\}$ NMR (162 MHz, DMSO-d_6): δ = 15.70 (d, $^2J_{PP}$ = 415 Hz, P_B), 15.06 (d, $^2J_{PP}$ = 414 Hz, P_B) ppm. Pt satellites $^1J_{P, Pt}$ = 2456 Hz.

^{195}Pt NMR (86 MHz, DMSO-d_6): δ = -4504 (t, $^1J_{Pt, P}$ ≈ 2456 Hz).

HRMS (ESI) for $C_{47}H_{46}BrN_5O_4P_2Pt$ [M+H] calcd. 1080.1851. Found: 1080.1777.

1.11. Complex 7

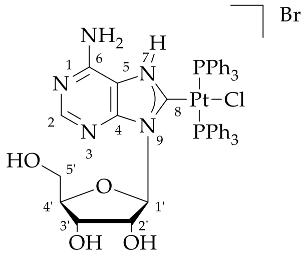

To a mixture of **5** and **7** in DMSO-d6, already at maximum conversion (3:7) and with an adenosine concentration of 8 mM, 10 equivalents of NaCl were added (2 mg). The evolution of the resulting mixture at room temperature was followed by ^{1}H NMR. After 17 days, compound **7** is the only species detected in solution, by NMR, but was not isolated and the analysed solution contains NaCl.

^{1}H NMR (400 MHz, DMSO-*d6*): δ 14.49 (s, 1H, 7-NH), 7.87 (s, 1H, H-2), 8.1-7.12 (m, 32H, 2×PPh3 + NH2), 6.48 (d, 1H, H-1′, $^3J_{H\text{-}1', H\text{-}2'}$ ≈ 5.6 Hz), 5.18 (d, 1H, OH-3′, $^3J_{OH\text{-}3', H\text{-}3'}$ ≈ 5.6 Hz), 4.91 (d, 1H, 2′-OH, $^3J_{OH\text{-}2', H\text{-}2'}$ ≈ 6.8 Hz), 4.69 (dd, 1H, 5′-OH, $^3J_{OH\text{-}5', H\text{-}5a}$ = 5.6 Hz, $^3J_{OH\text{-}5', H\text{-}5b}$ = 6.0 Hz), 4.59 (ddd, 1H, H-2′, $^3J_{H\text{-}2', H\text{-}1'}$ = $^3J_{H\text{-}2', OH\text{-}2'}$ = $^3J_{H\text{-}2', H\text{-}3'}$ ≈ 5.6 Hz), 4.27 (ddd, 1H, H-3′, $^3J_{H\text{-}3',H\text{-}2'}$ ≈ $^3J_{H\text{-}3', OH\text{-}3'}$ ≈ $^3J_{H\text{-}3', H\text{-}4'}$ ≈ 5.6 Hz), 3.64 (ddd, 1H, H-4′, $^3J_{H\text{-}4', H\text{-}3'}$ = $^3J_{H\text{-}4', H\text{-}5'a}$ = $^3J_{H\text{-}4', H\text{-}5'b}$ ≈ 4.5 Hz), 3.35-3.23* (m, 2H, H-5′) ppm. *Determined by HSQC

^{13}C{^{1}H} NMR (100 MHz, DMSO-d6): δ 155.2 (C-8), 152.3 (C-2), 150.0 (C-6), 147.5 (C-4), 135-127 (2×P(C_6H_5)3), 111.7 (C-5), 94.5 (C-1′), 84.7 (C-4′), 71.4 (C-2′), 69.9 (C-3′), 61.3 (C-5′) ppm.

^{31}P{^{1}H} NMR (162 MHz, DMSO-d6): δ = 18.62 (d, $^2J_{PP}$ = 420 Hz, P_A), 17.79 (d, $^2J_{PP}$ = 420 Hz, P_B) ppm. Pt satellites $^1J_{P, Pt}$ = 2530 Hz.

^{195}Pt NMR (86 MHz, DMSO-d6): δ = -4340 (t, $^1J_{Pt, P}$ = 2530 Hz).

HRMS (ESI) for $C_{46}H_{44}ClN_5O_4P_2Pt$ [M+H] calcd. 1022.2199. Found: 1022.2123.

2. NMR and MS spectra

2.1. NMR spectra

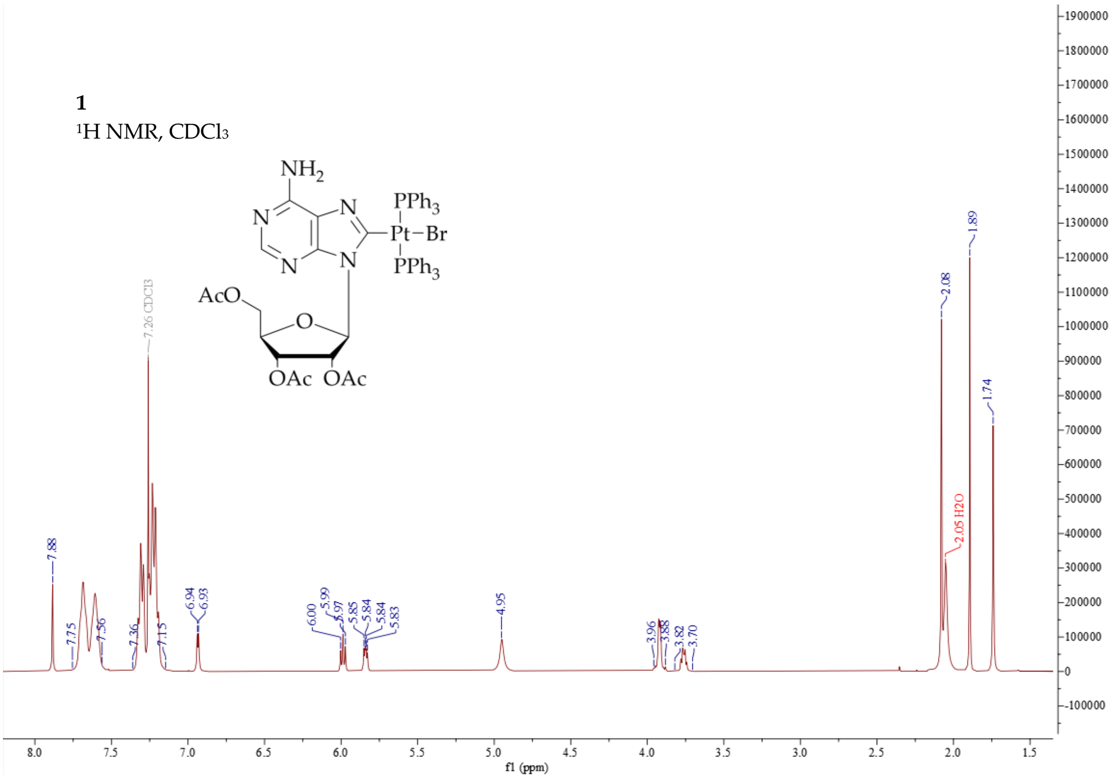

Figure S1. ^{1}H NMR spectrum of compound **1** recorded in $CDCl_3$.

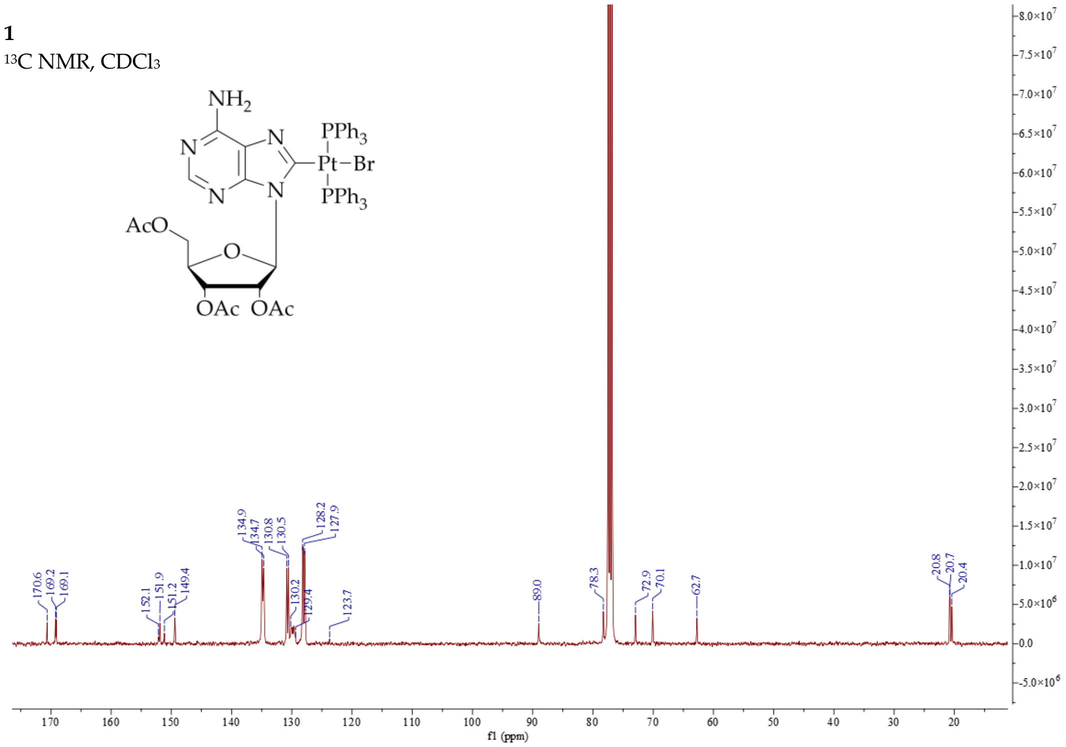

Figure S2. $^{13}C\{^{1}H\}$ NMR spectrum of compound **1** recorded in $CDCl_3$.

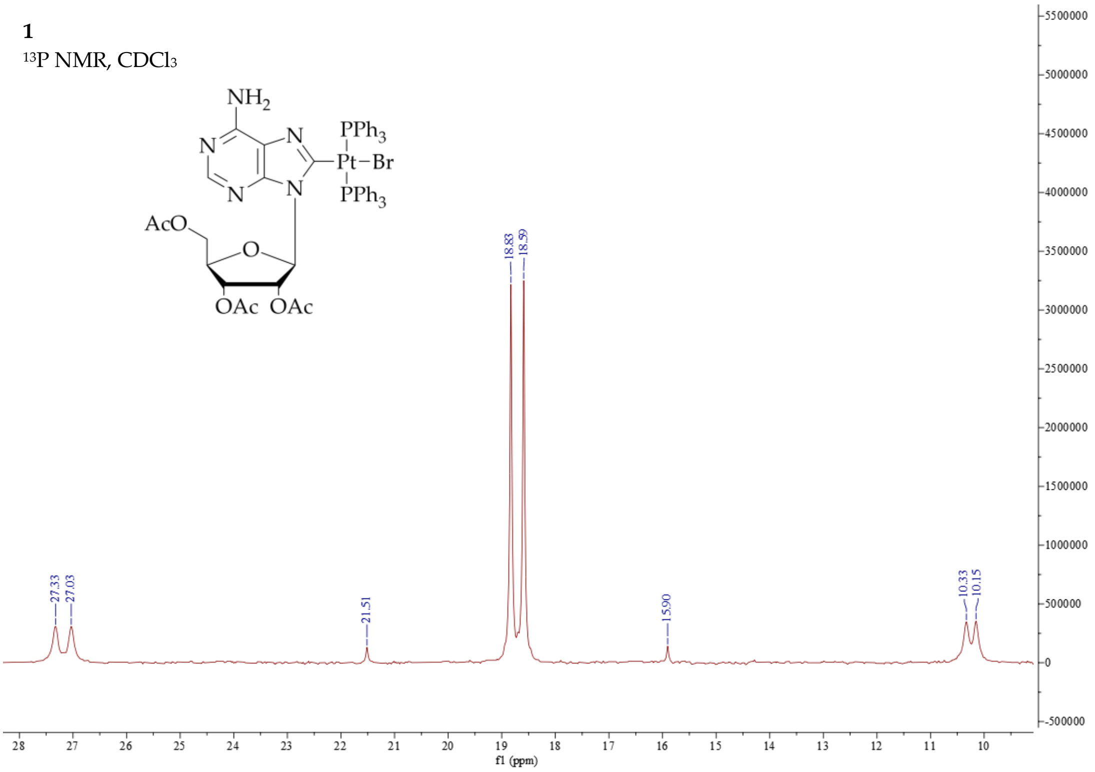

Figure S3. $^{31}P\{^{1}H\}$ NMR spectrum of compound **1** recorded in $CDCl_3$.

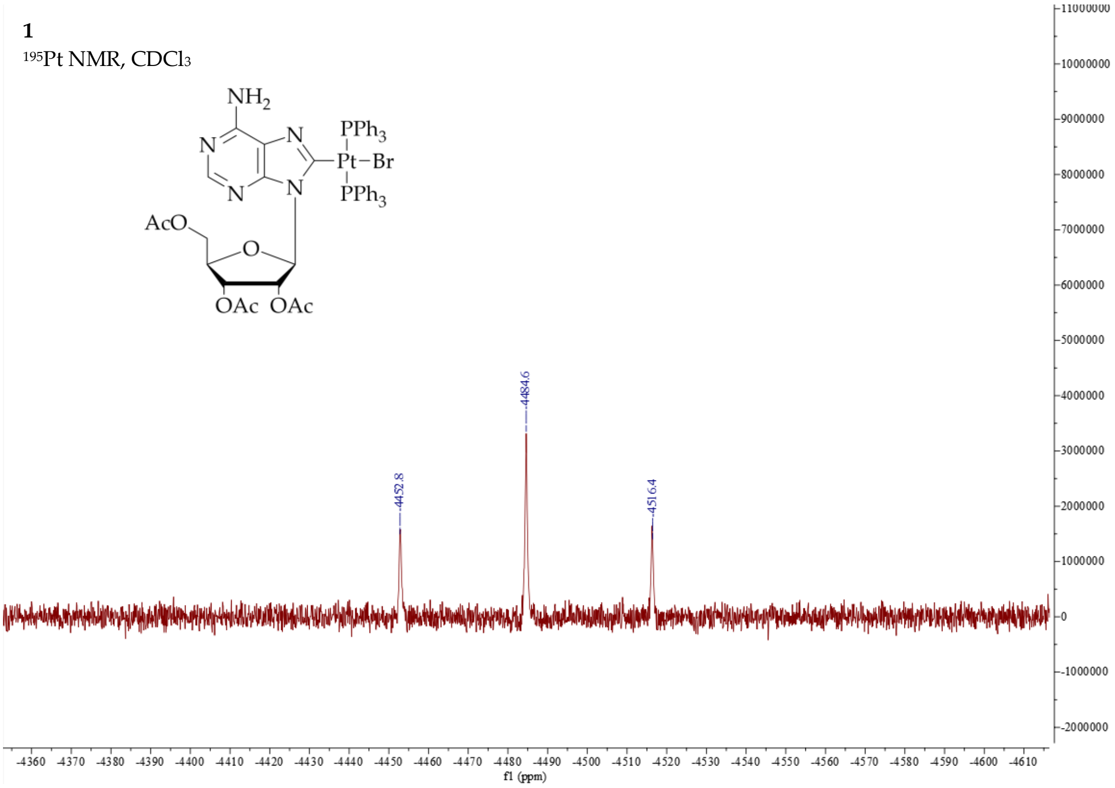

Figure S4. ^{195}Pt NMR spectrum of compound **1** recorded in $CDCl_3$.

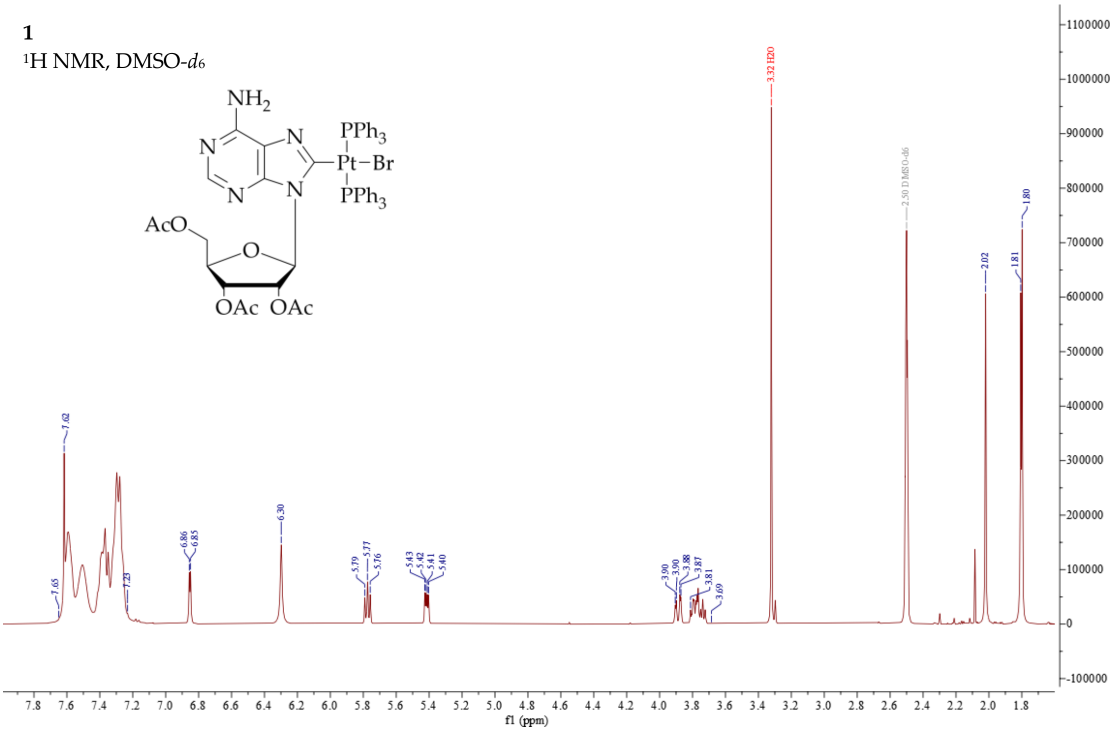

Figure S5. ^{1}H NMR spectrum of compound **1** recorded in DMSO-*d*$_6$.

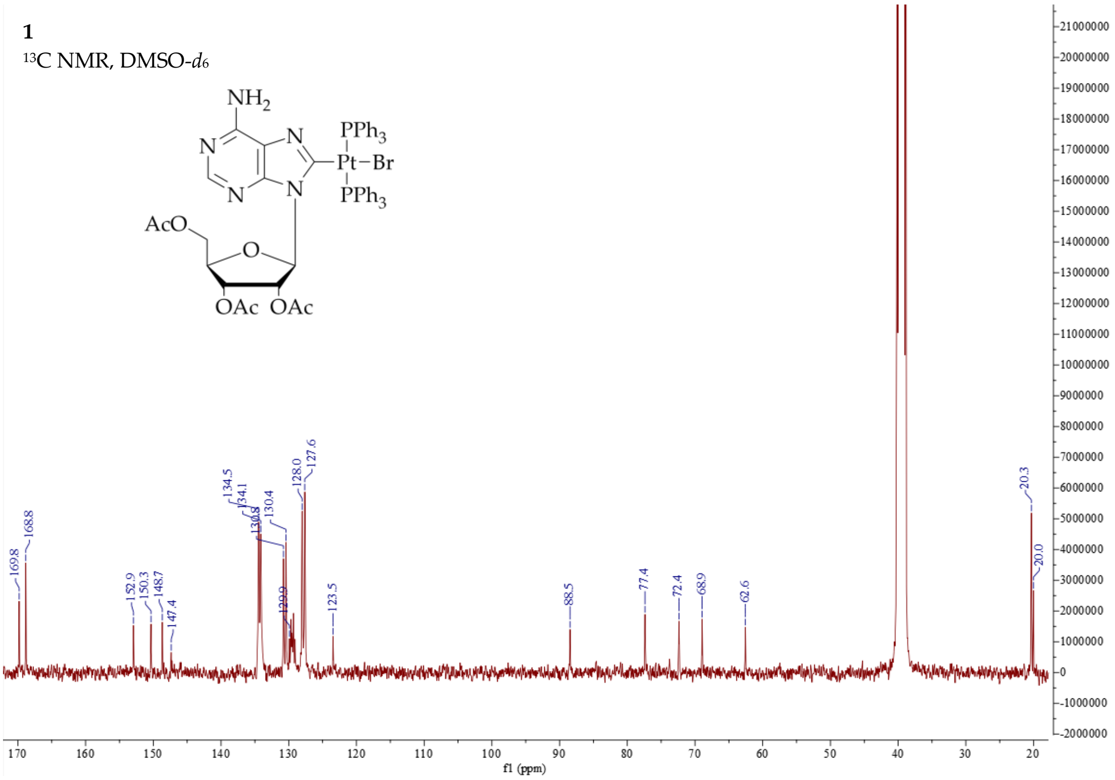

Figure S6. ^{13}C{^{1}H} NMR spectrum of compound **1** recorded in DMSO-*d*$_6$.

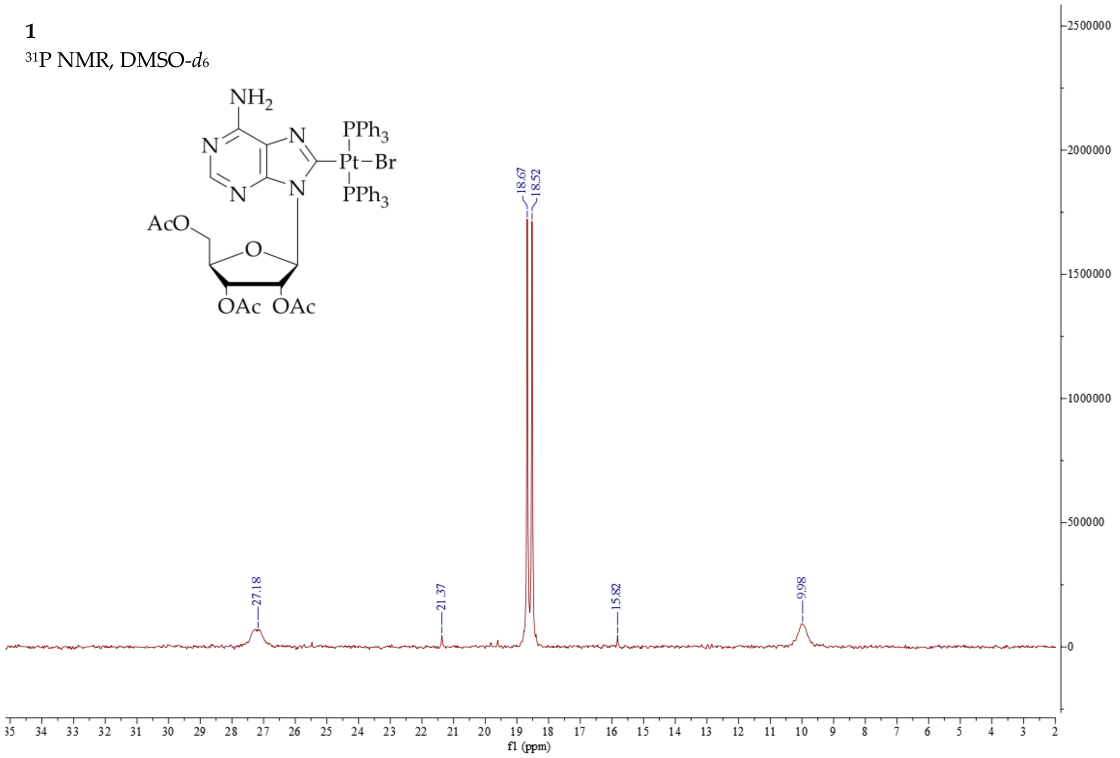

Figure S7. $^{31}P\{^{1}H\}$ NMR spectrum of compound **1** recorded in DMSO-d_6.

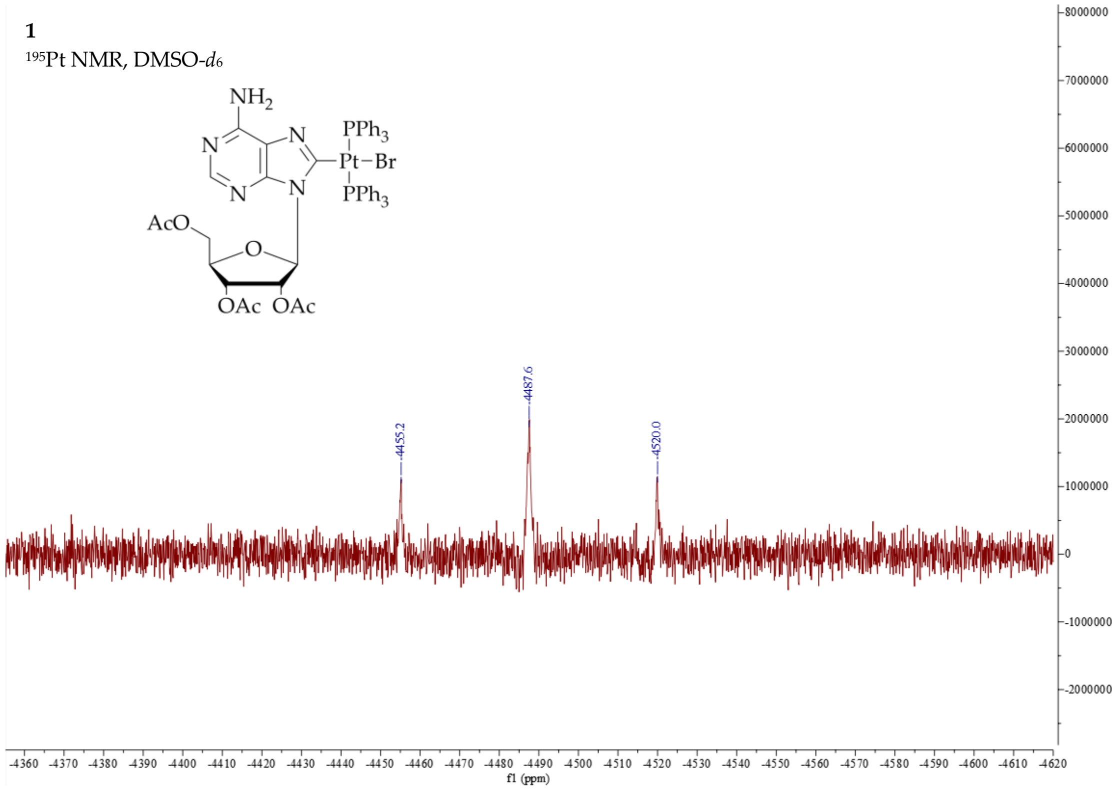

Figure S8. ^{195}Pt NMR spectrum of compound **1** recorded in DMSO-d_6.

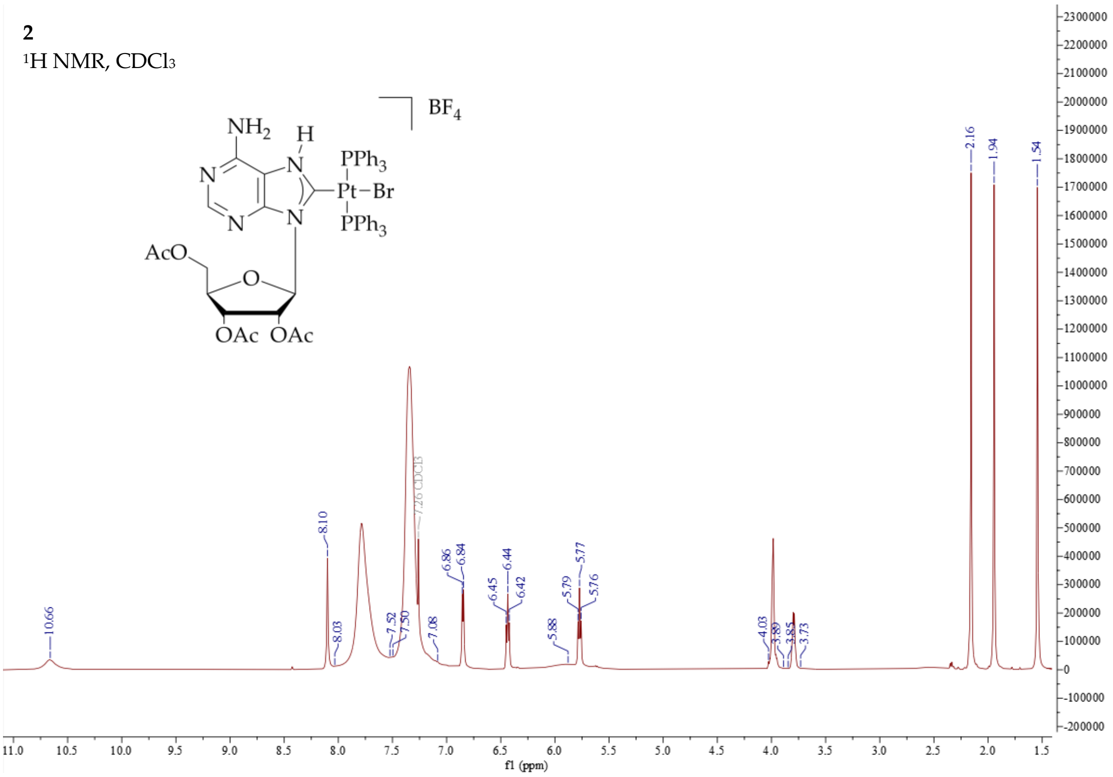

Figure S9. ^{1}H NMR spectrum of compound **2** recorded in $CDCl_3$.

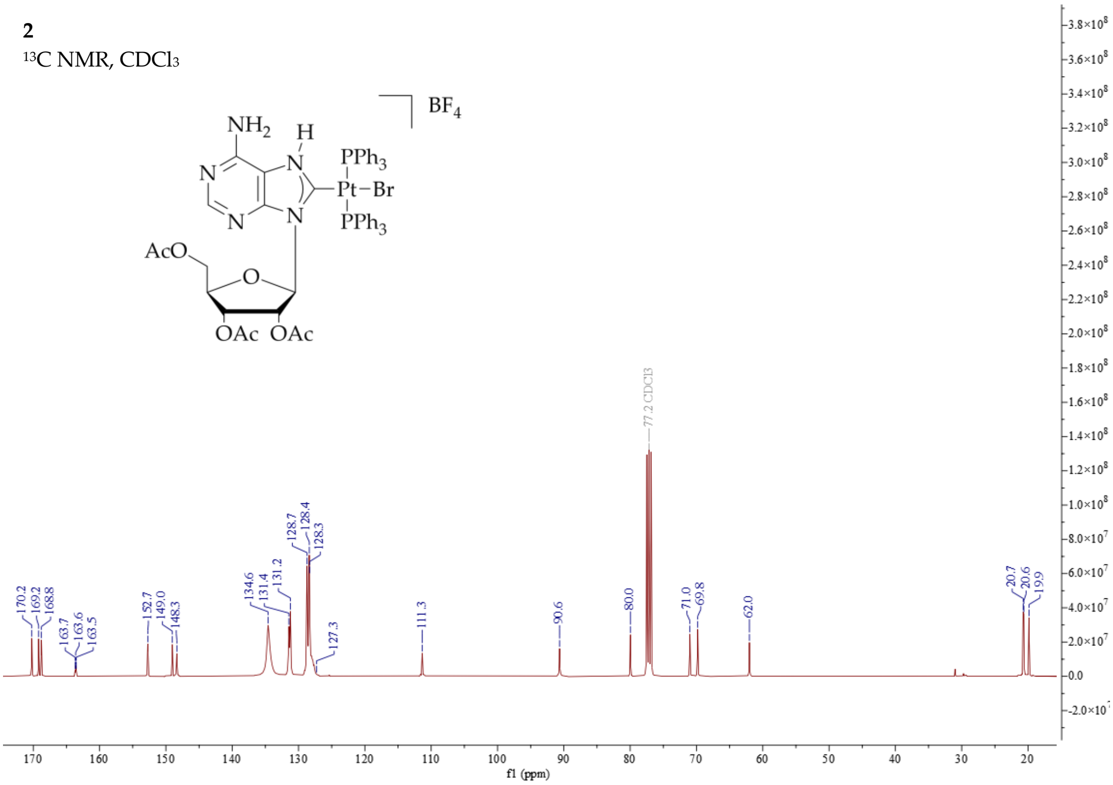

Figure S10. $^{13}C\{^{1}H\}$ NMR spectrum of compound **2** recorded in $CDCl_3$.

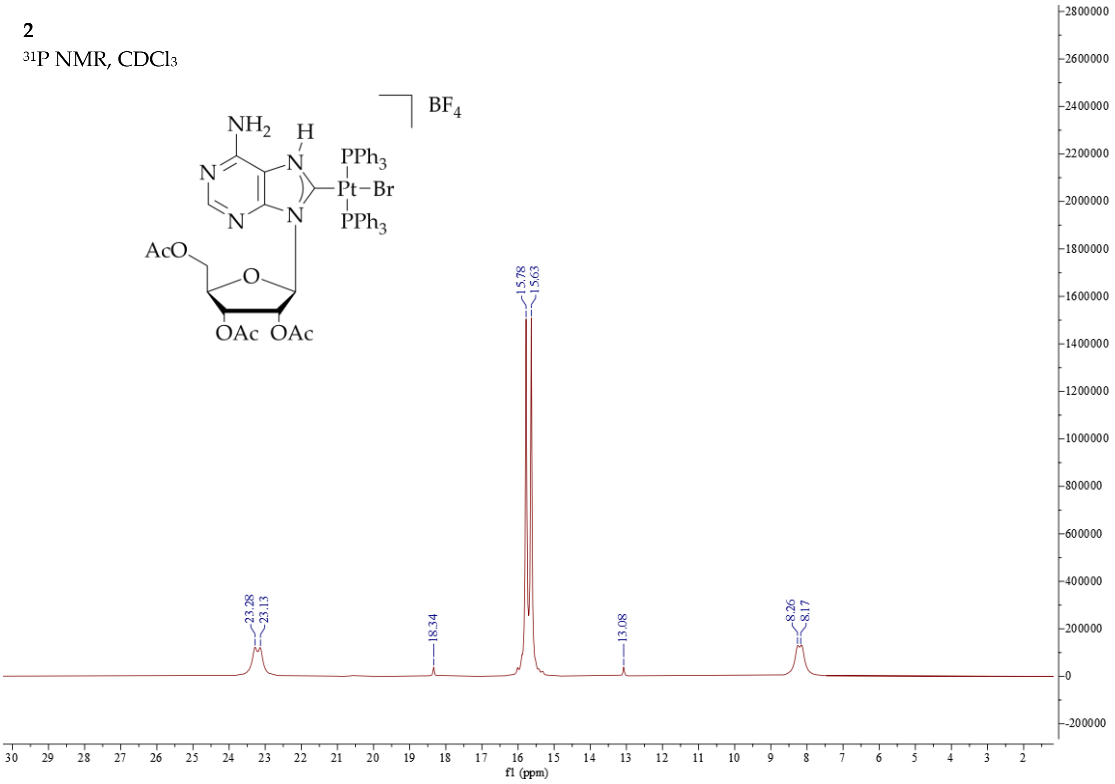

Figure S11. $^{31}P\{^{1}H\}$ NMR spectrum of compound **2** recorded in CDCl3₆.

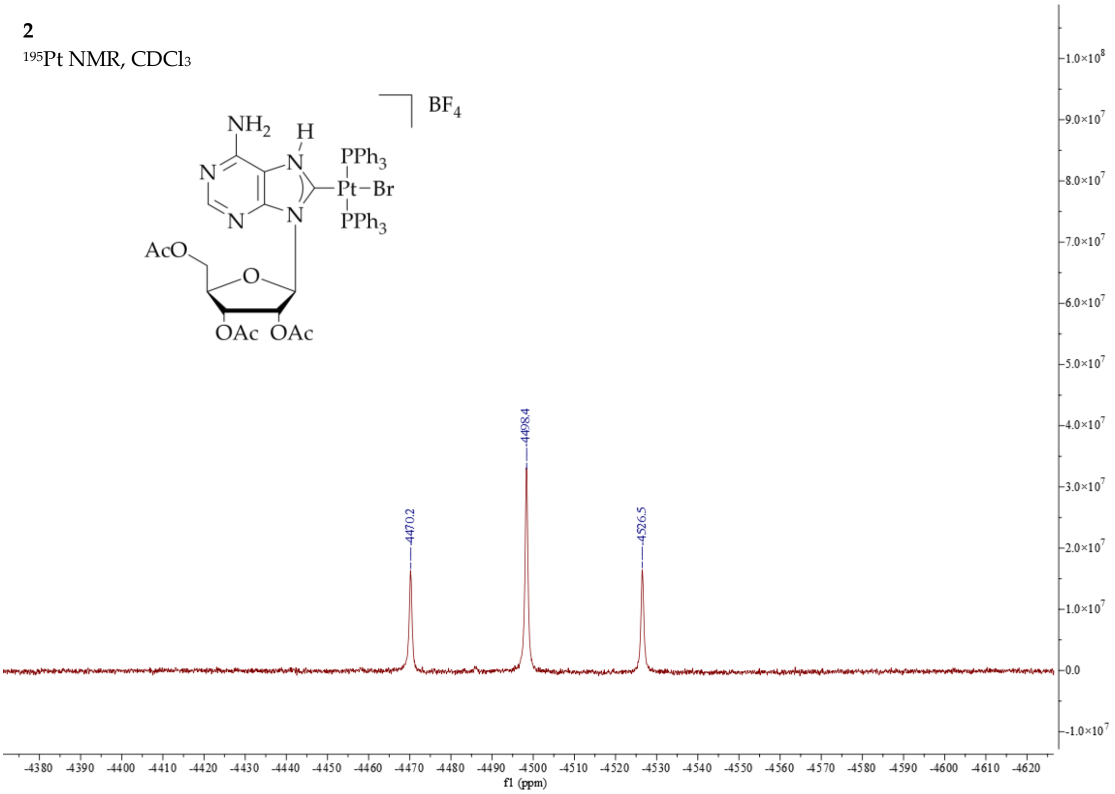

Figure S12. ^{195}Pt NMR spectrum of compound **2** recorded in $CDCl_3$.

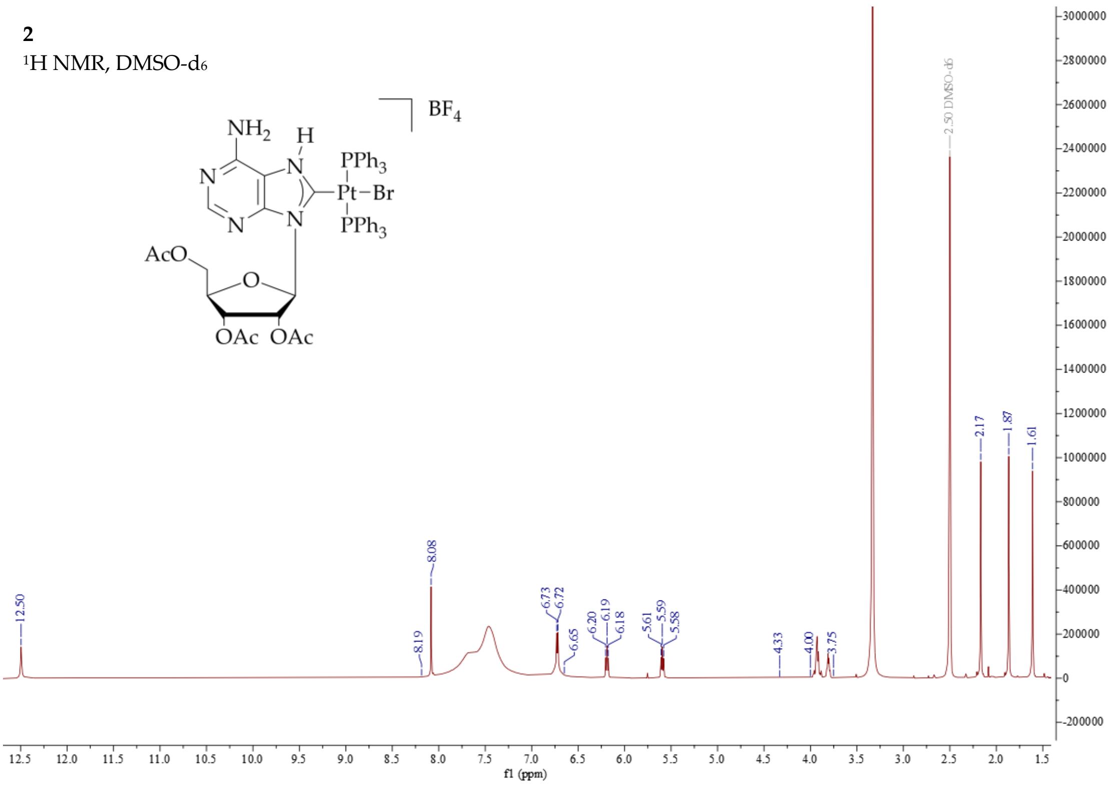

Figure S13. ^{1}H NMR spectrum of compound **2** recorded in DMSO-d_6.

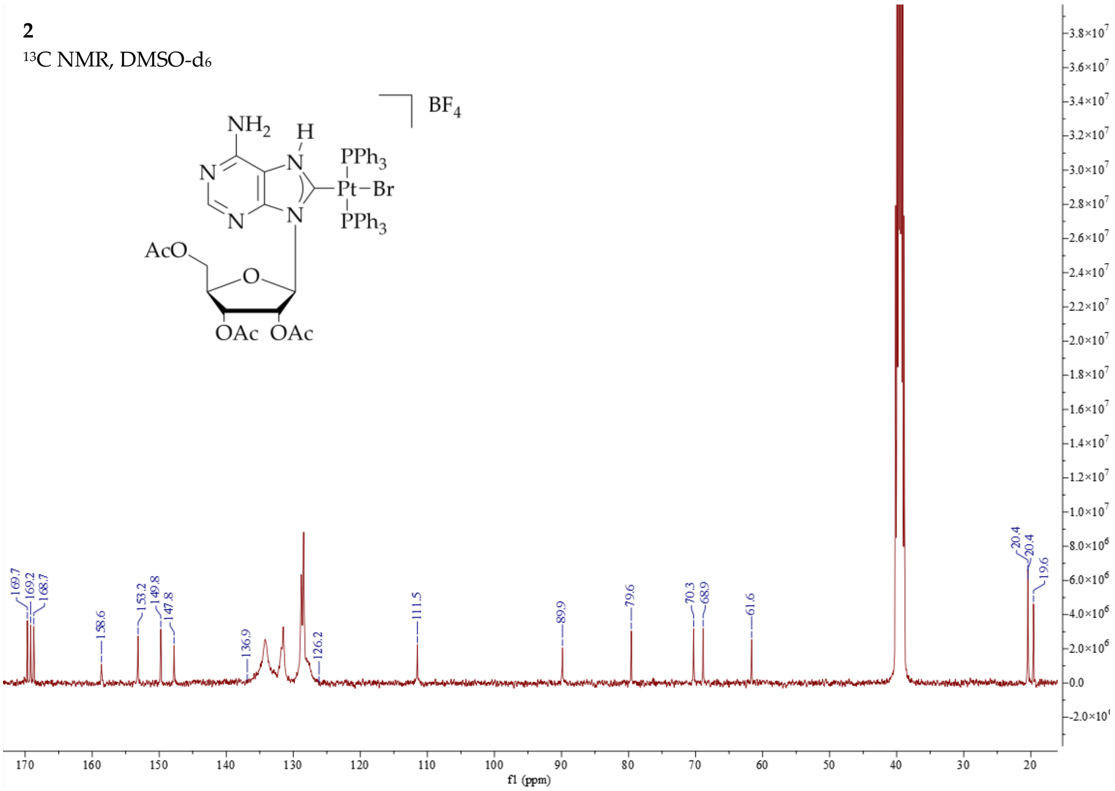

Figure S14. $^{13}C\{^{1}H\}$ NMR spectrum of compound **2** recorded in DMSO-d_6.

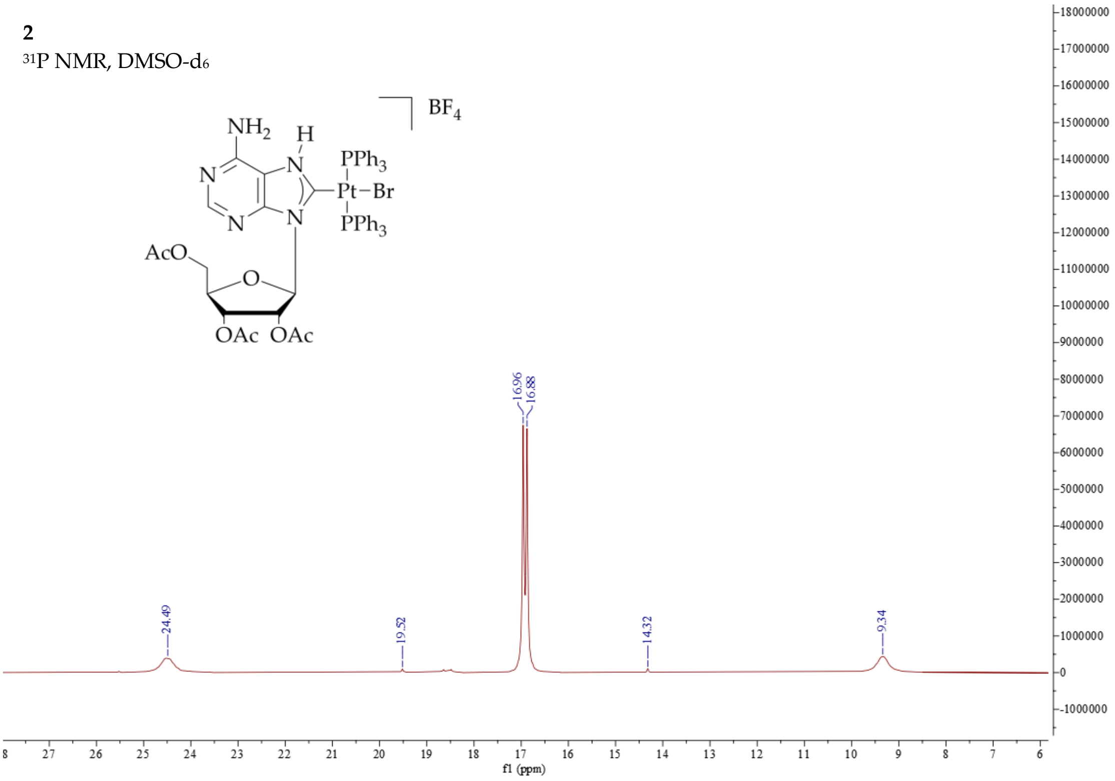

Figure S15. $^{31}P\{^{1}H\}$ NMR spectrum of compound **2** recorded in DMSO-*d*6.

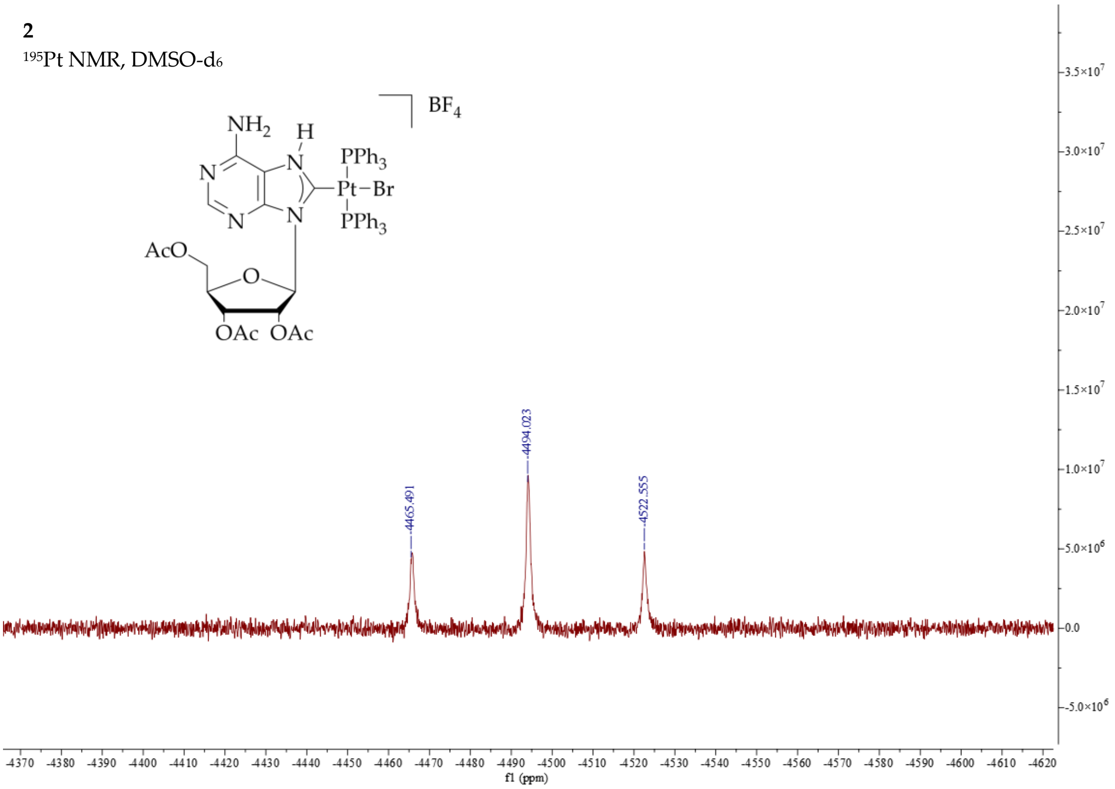

Figure S16. ^{195}Pt NMR spectrum of compound **2** recorded in DMSO-*d*6.

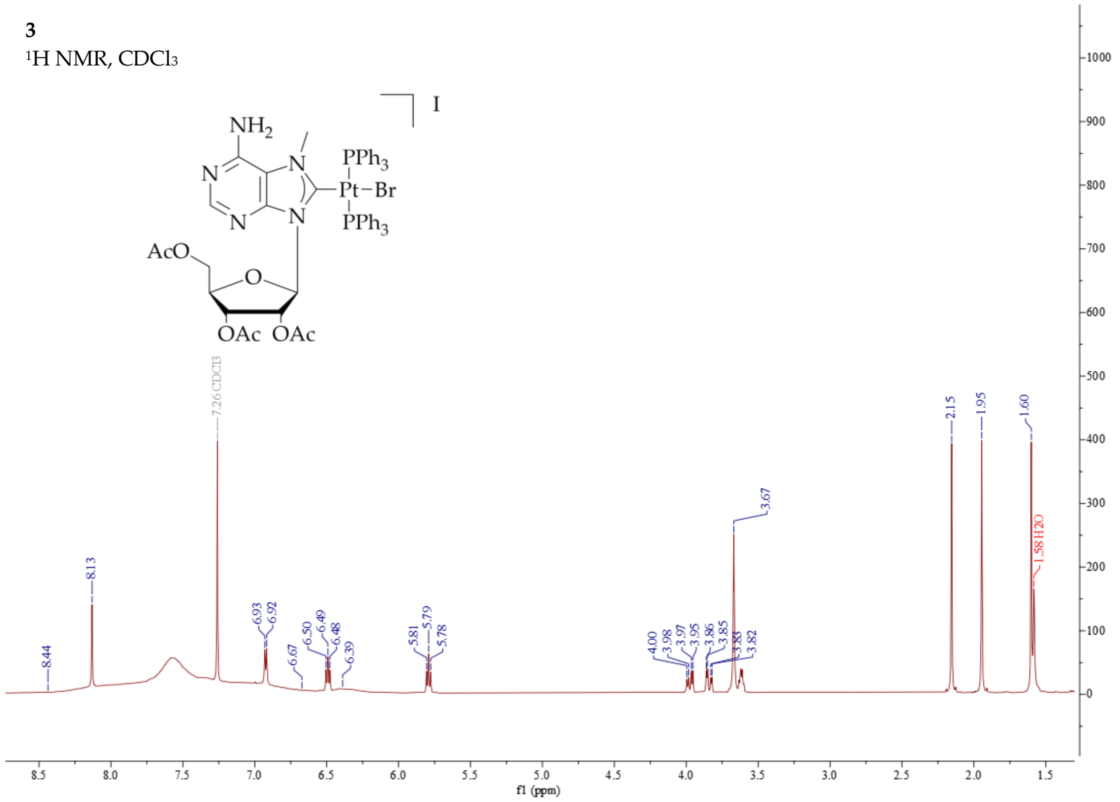

Figure S17. ^{1}H NMR spectrum of compound **3** recorded in $CDCl_3$.

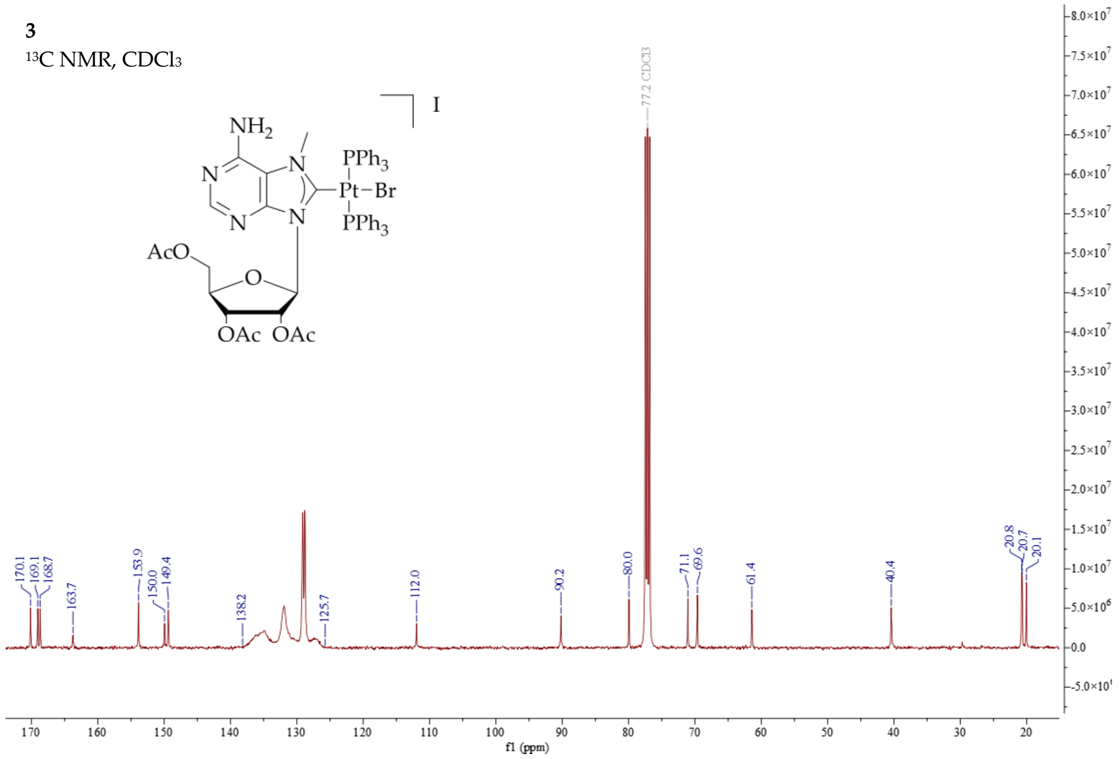

Figure S18. $^{13}C\{^{1}H\}$ NMR spectrum of compound **3** recorded in $CDCl_3$.

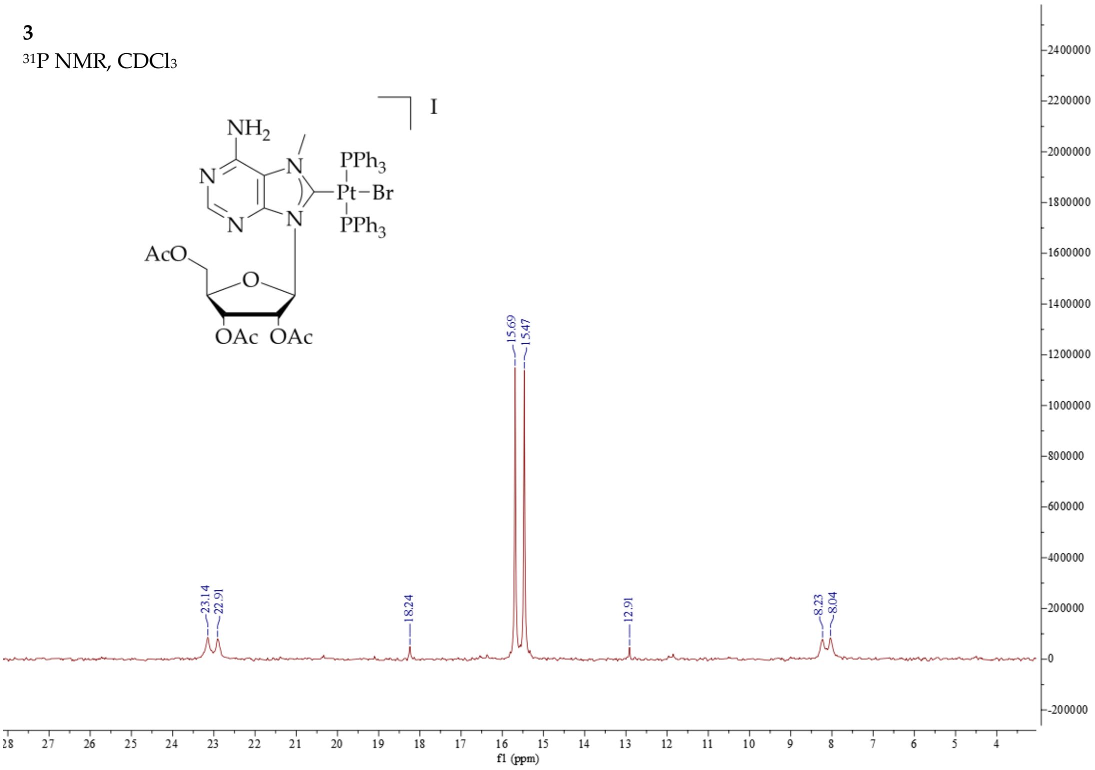

Figure S19. $^{31}P\{^{1}H\}$ NMR spectrum of compound **3** recorded in $CDCl_3$.

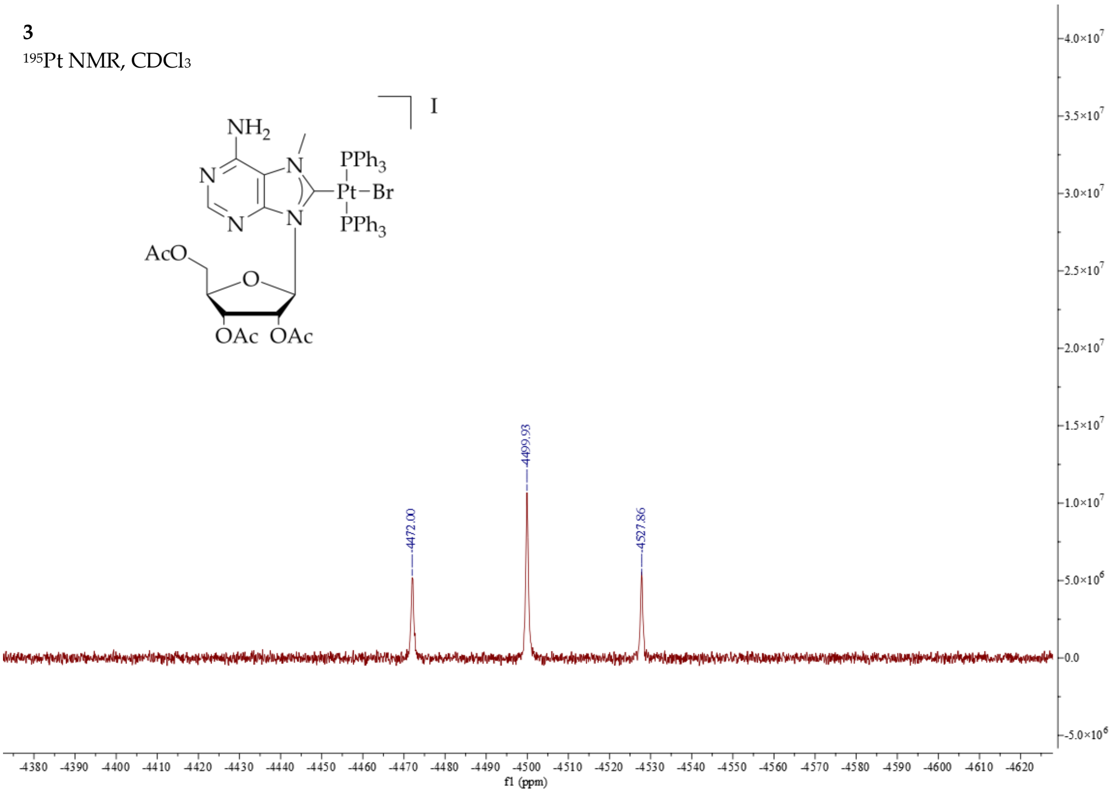

Figure S20. ^{195}Pt NMR spectrum of compound **3** recorded in $CDCl_3$.

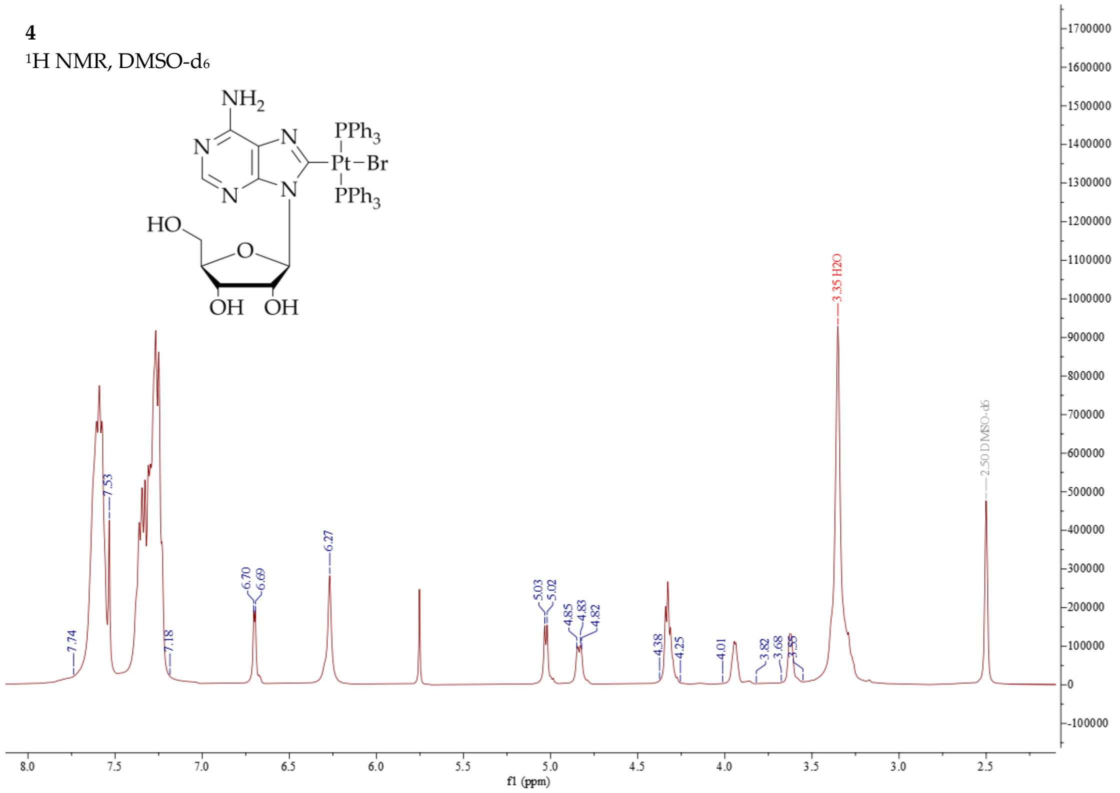

Figure S21. ^{1}H NMR spectrum of compound **4** recorded in DMSO-*d*$_6$.

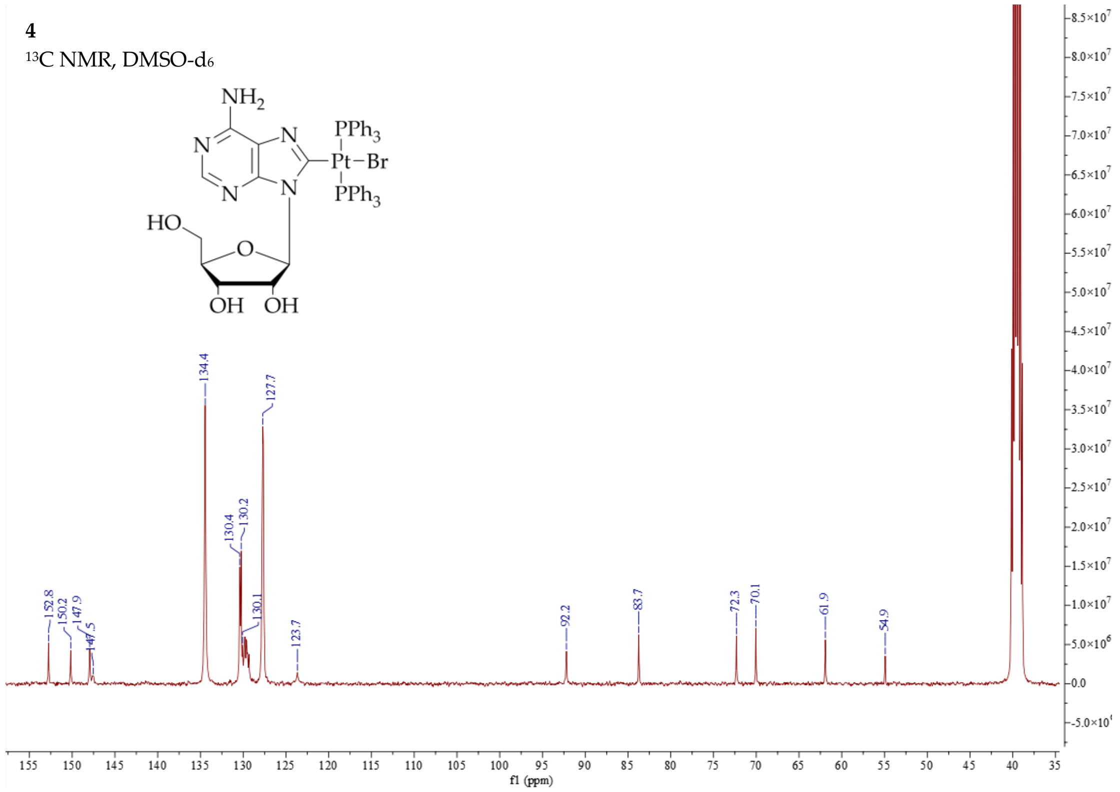

Figure S22. $^{13}C\{^{1}H\}$ NMR spectrum of compound **4** recorded in DMSO-*d*$_6$.

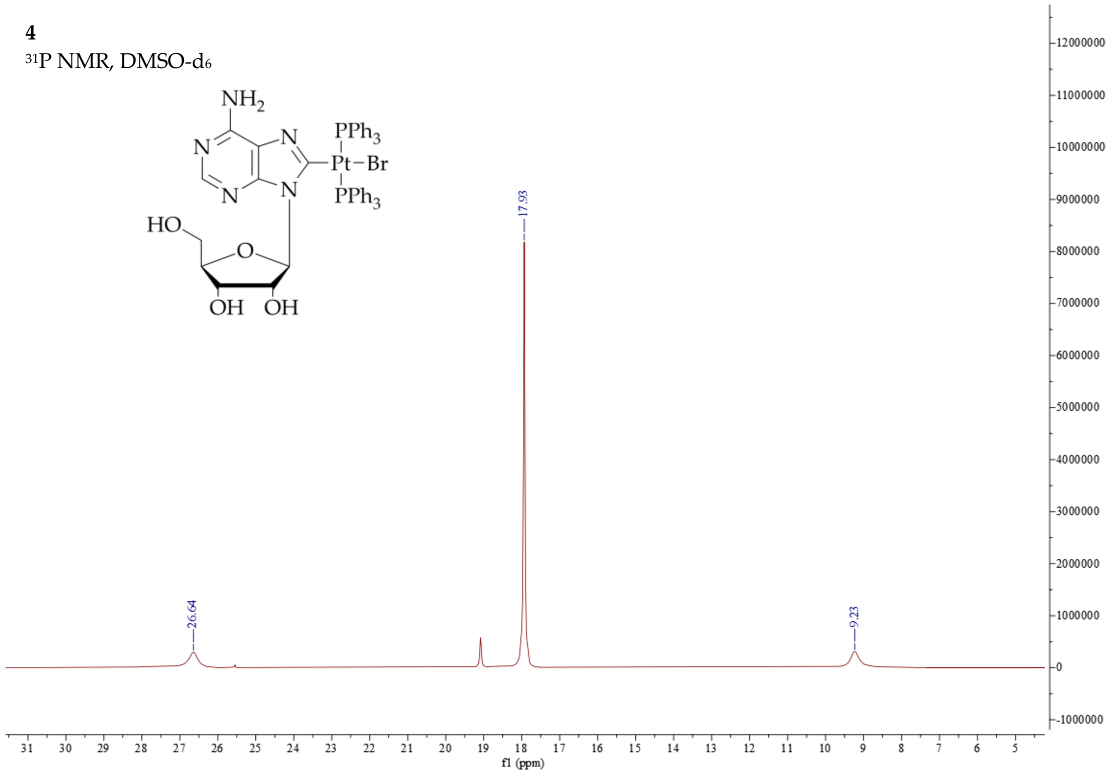

Figure S23. $^{31}P\{^{1}H\}$ NMR spectrum of compound **4** recorded in DMSO-*d*6.

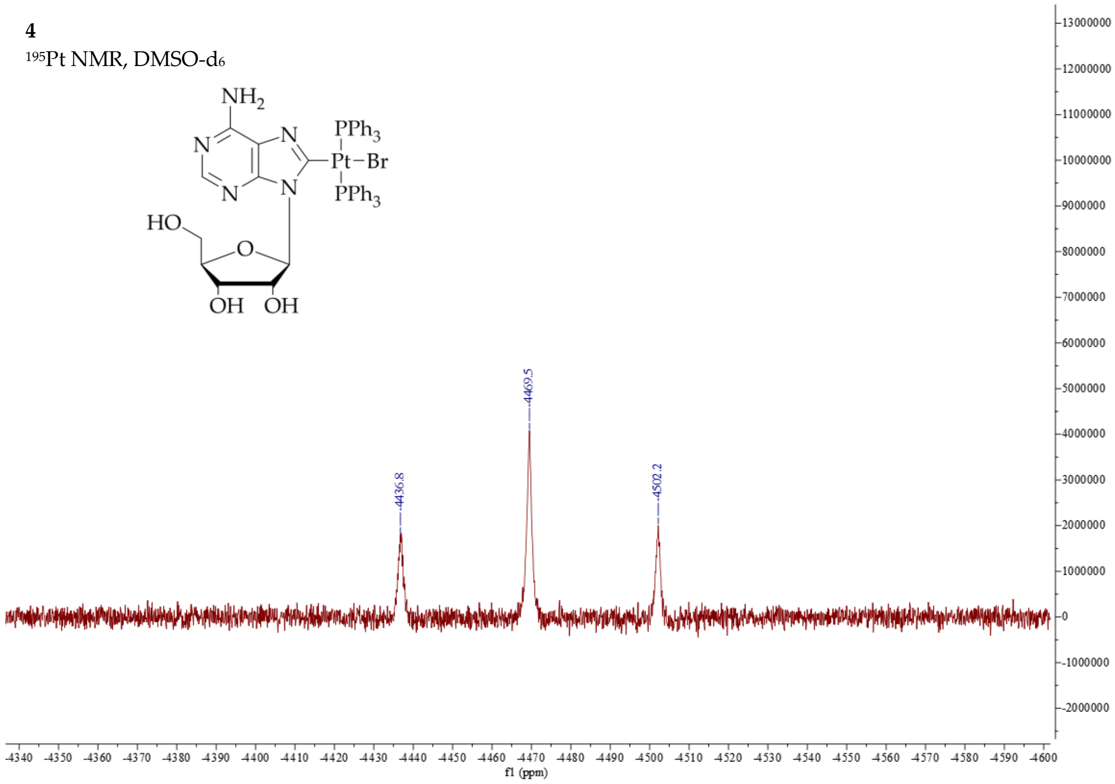

Figure S24. ^{195}Pt NMR spectrum of compound **4** recorded in DMSO-*d*6.

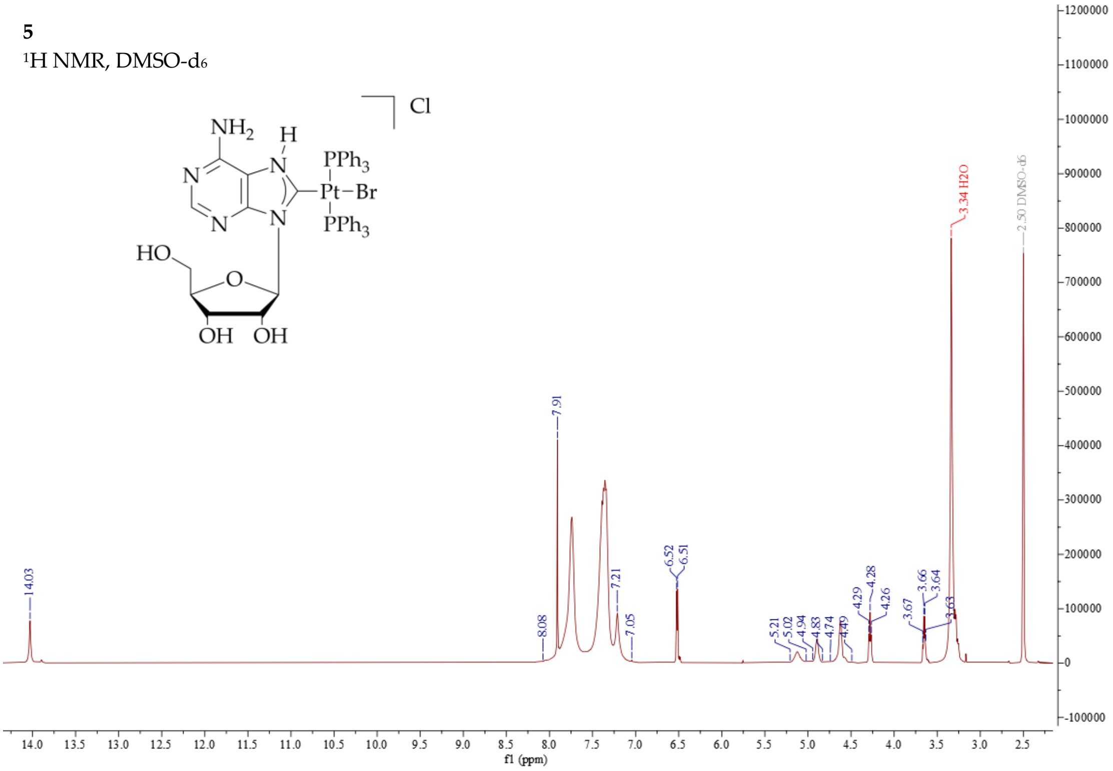

Figure S25. ^{1}H NMR spectrum of compound **5** recorded in DMSO-d_6.

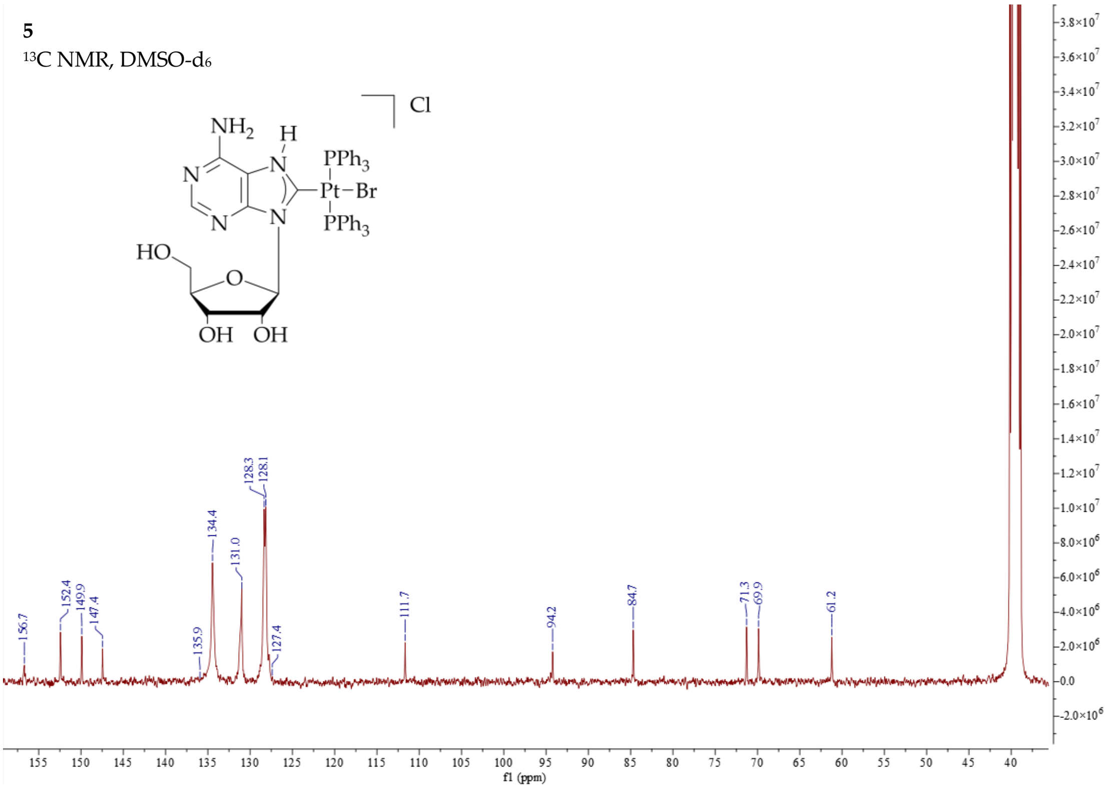

Figure S26. $^{13}C\{^{1}H\}$ NMR spectrum of compound **5** recorded in DMSO-d_6.

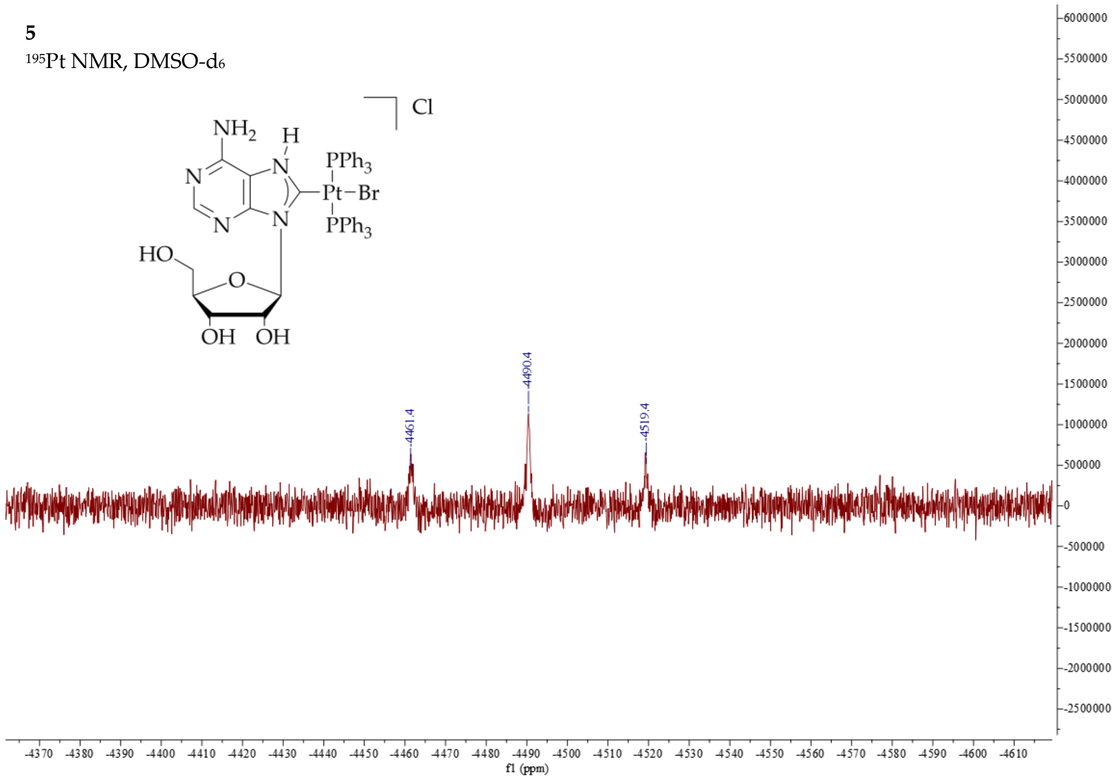

Figure S27. ^{195}Pt NMR spectrum of compound **5** recorded in DMSO-d_6.

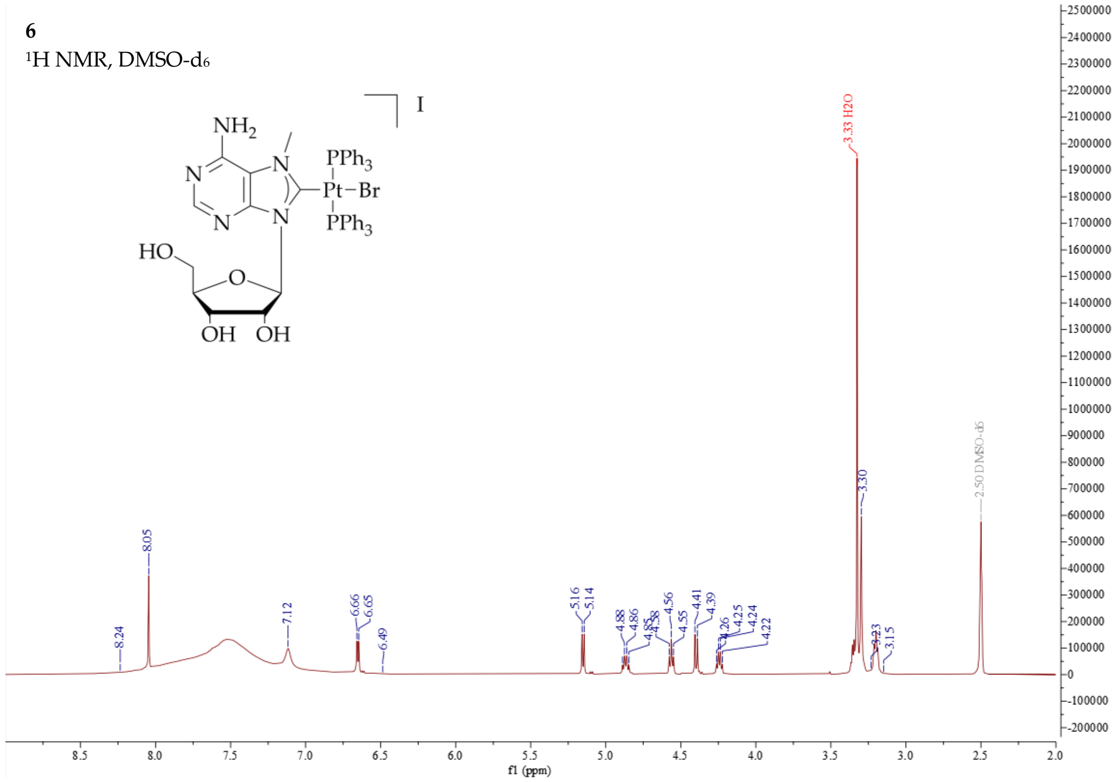

Figure S28. ^{1}H NMR spectrum of compound **6** recorded in DMSO-d_6.

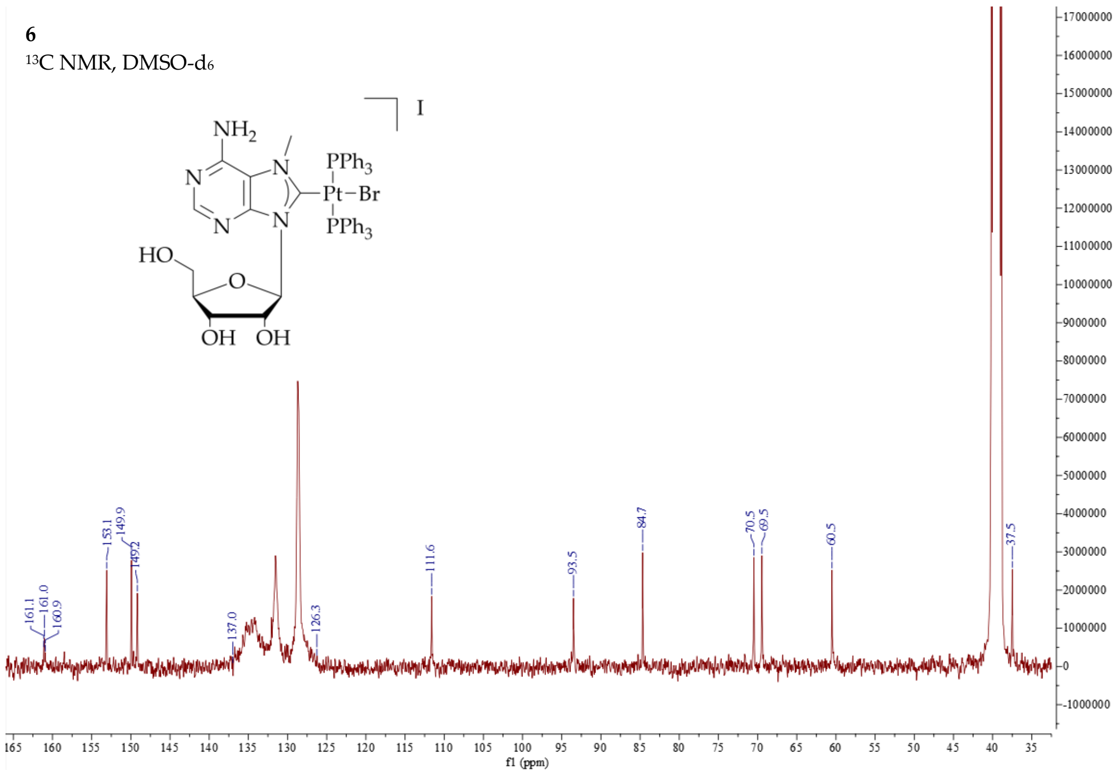

Figure S29. $^{13}C\{^1H\}$ NMR spectrum of compound **1** recorded in DMSO-d_6.

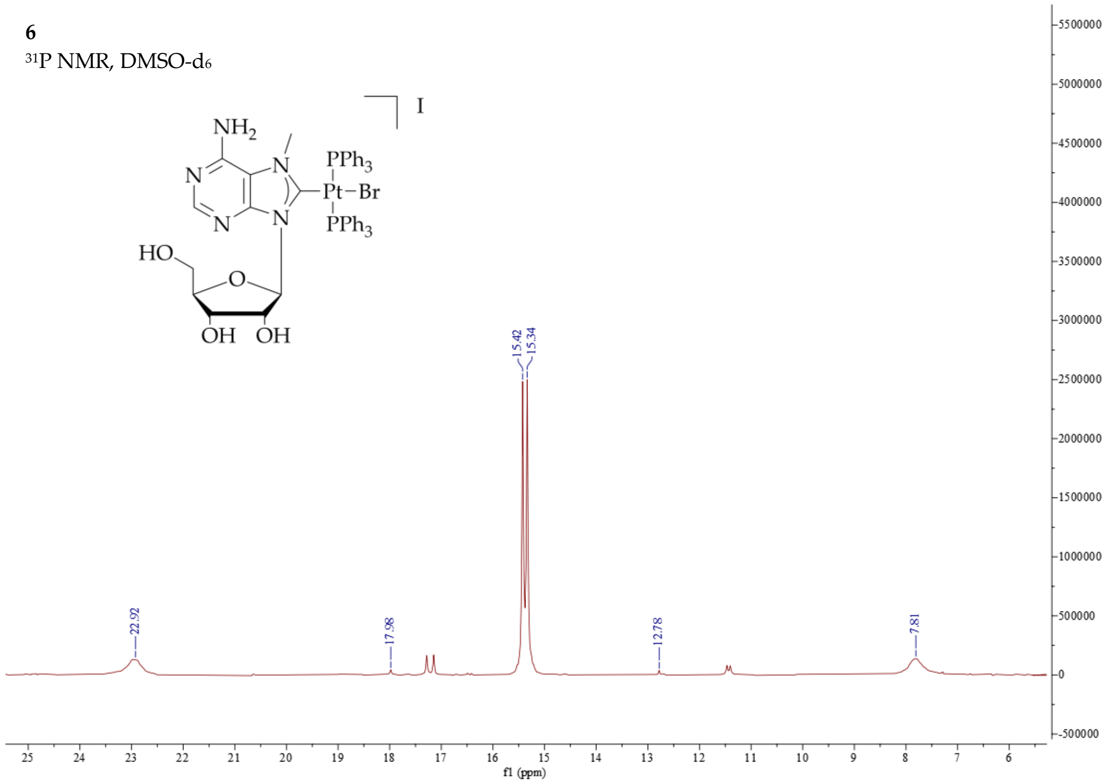

Figure S30. $^{31}P\{^1H\}$ NMR spectrum of compound **6** recorded in DMSO-d_6.

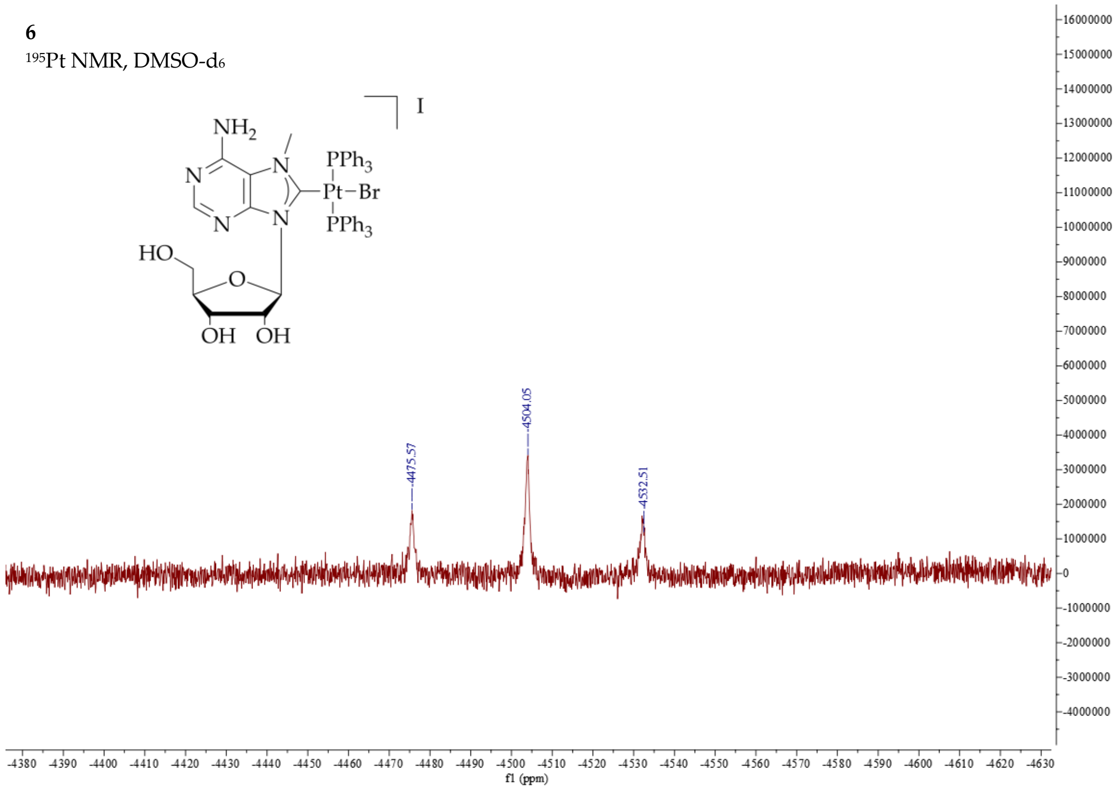

Figure S31. ^{195}Pt NMR spectrum of compound **6** recorded in DMSO-*d*6.

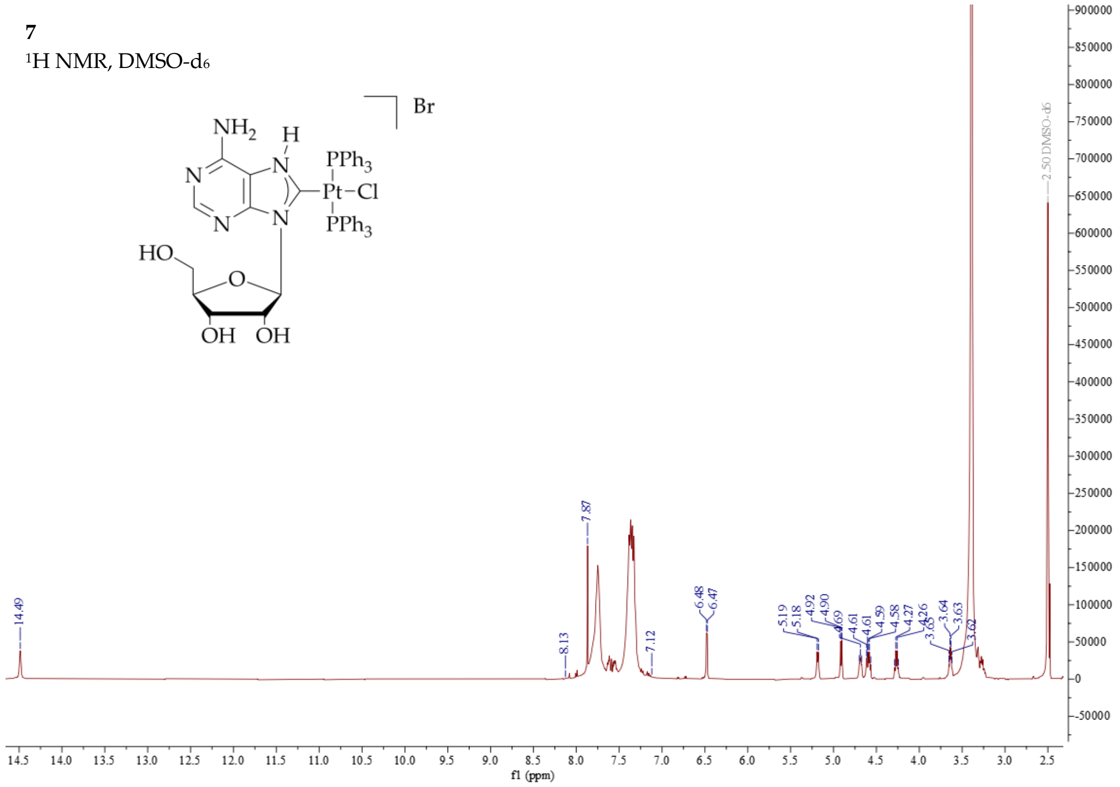

Figure S32. ^{1}H NMR spectrum of compound **7** recorded in DMSO-*d*6.

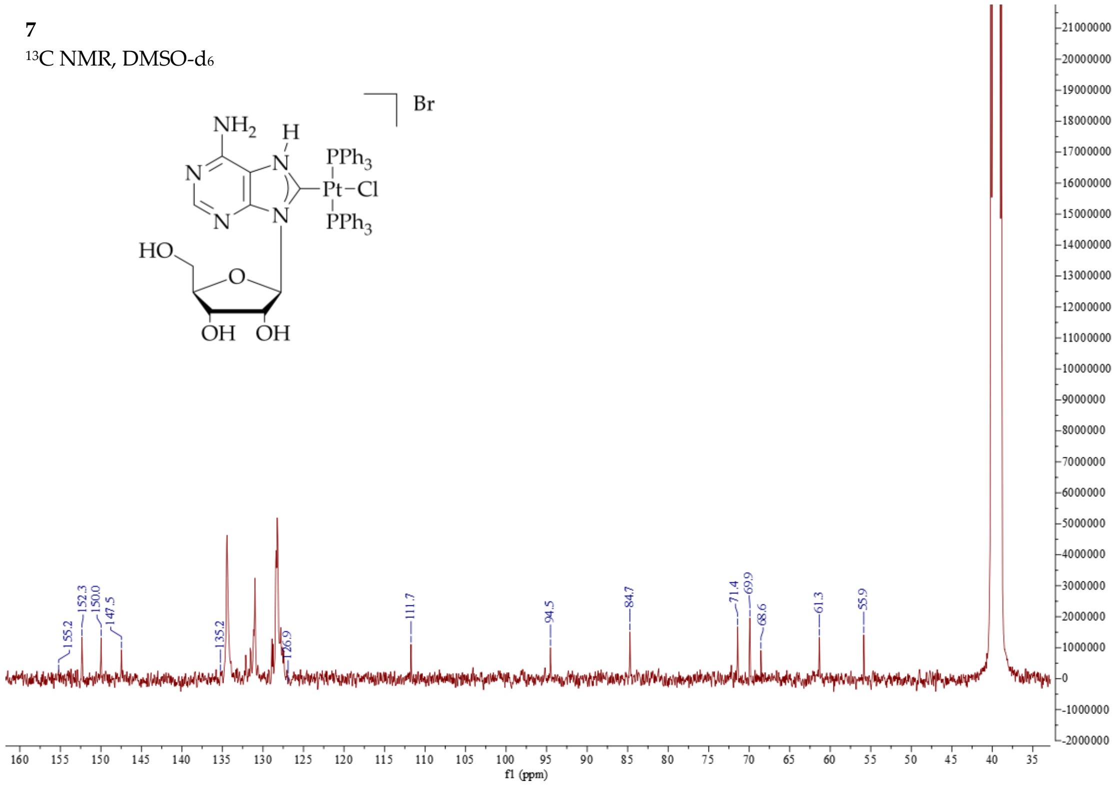

Figure S33. $^{13}C\{^{1}H\}$ NMR spectrum of compound **7** recorded in DMSO-d_6.

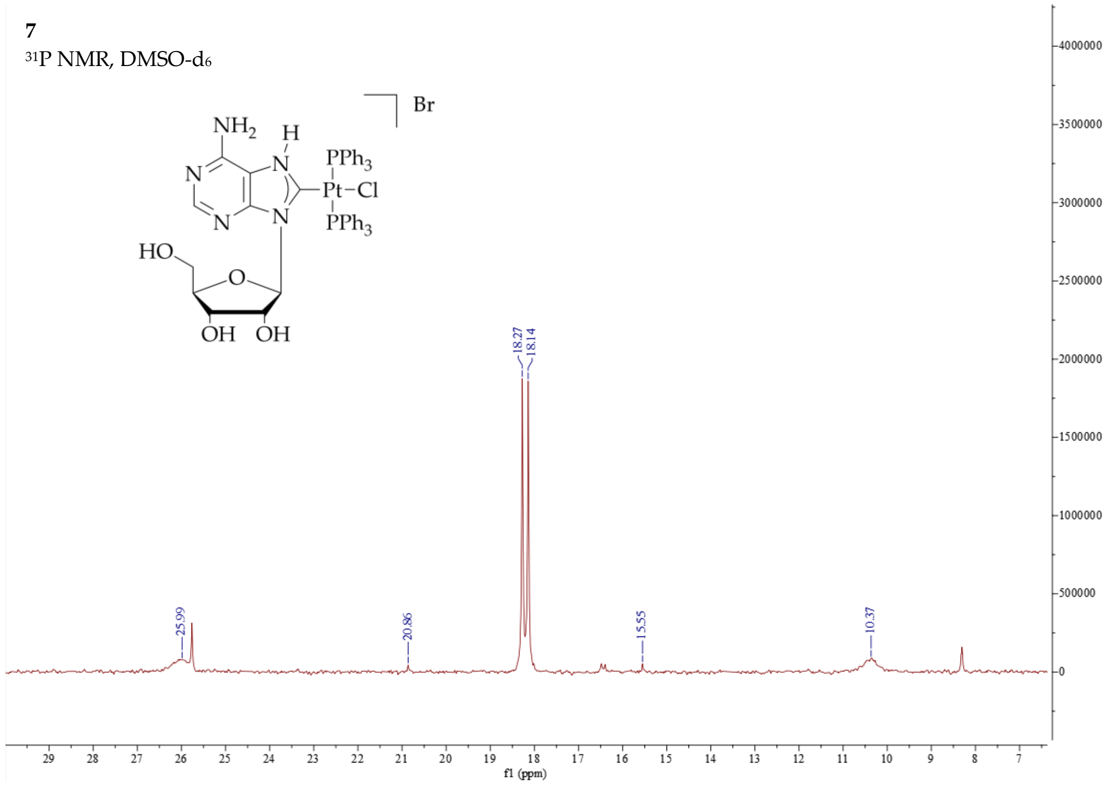

Figure S34. $^{31}P\{^{1}H\}$ NMR spectrum of compound **7** recorded in DMSO-d_6.

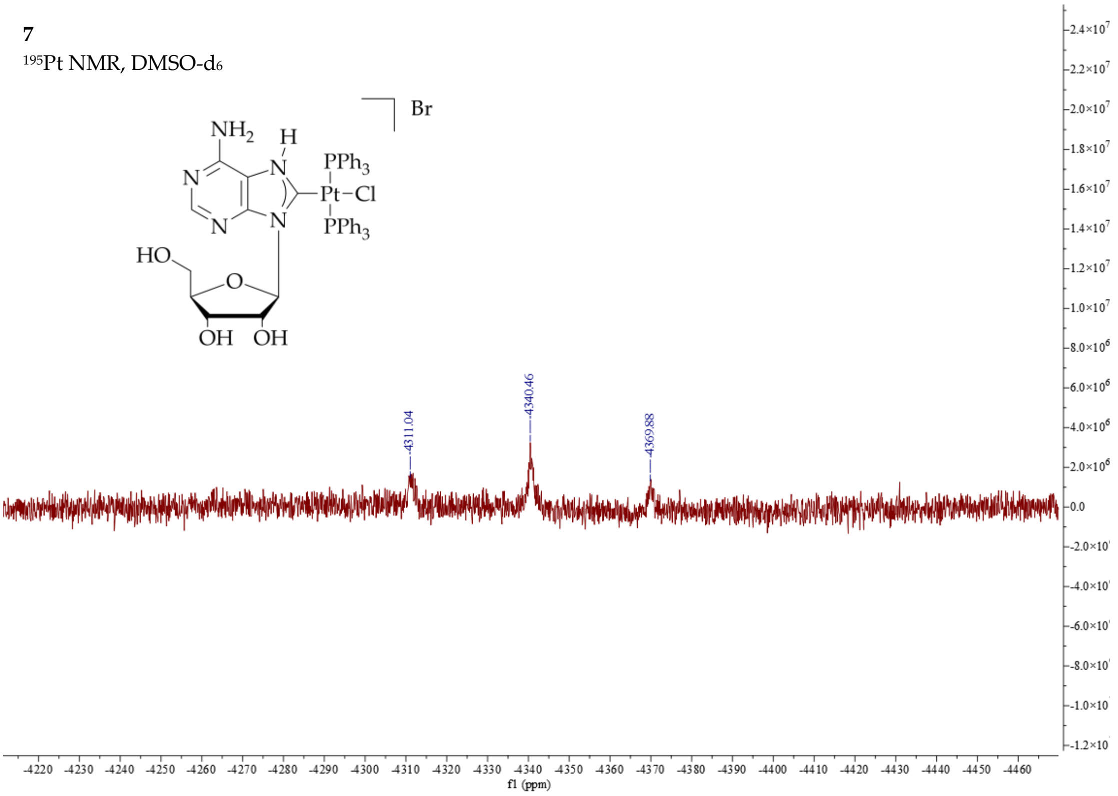

Figure S35. ^{195}Pt NMR spectrum of compound **7** recorded in DMSO-*d*$_6$.

2.2. HRMS spectra

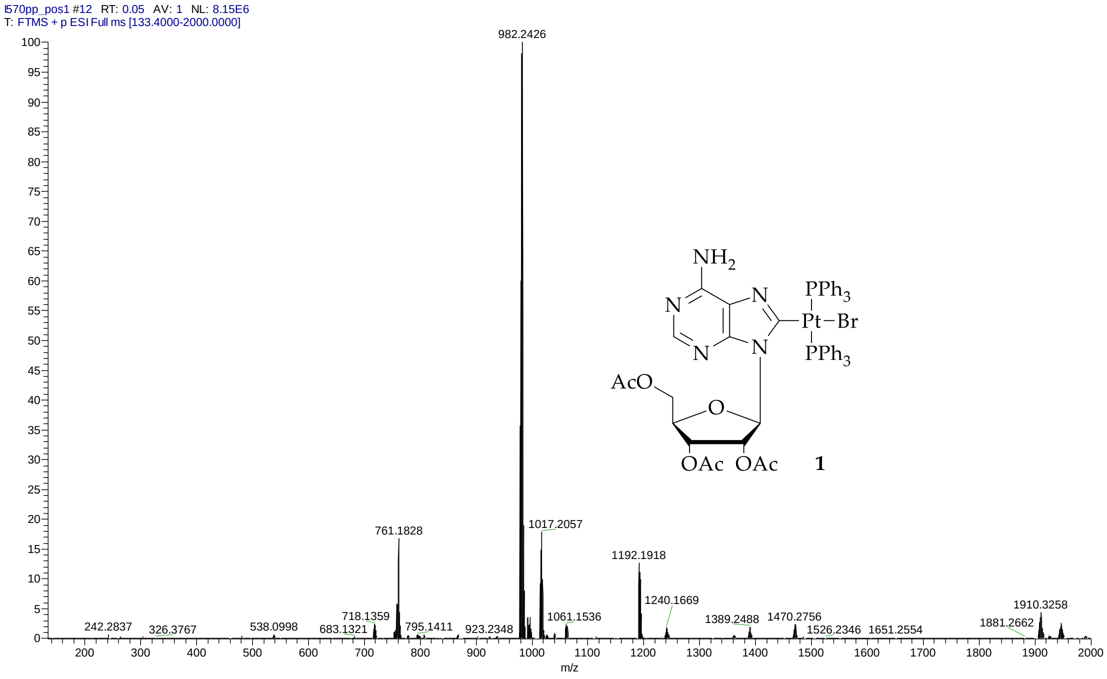

Figure S36. HRMS (ESI) of compound **1** (acquired in positive mode).

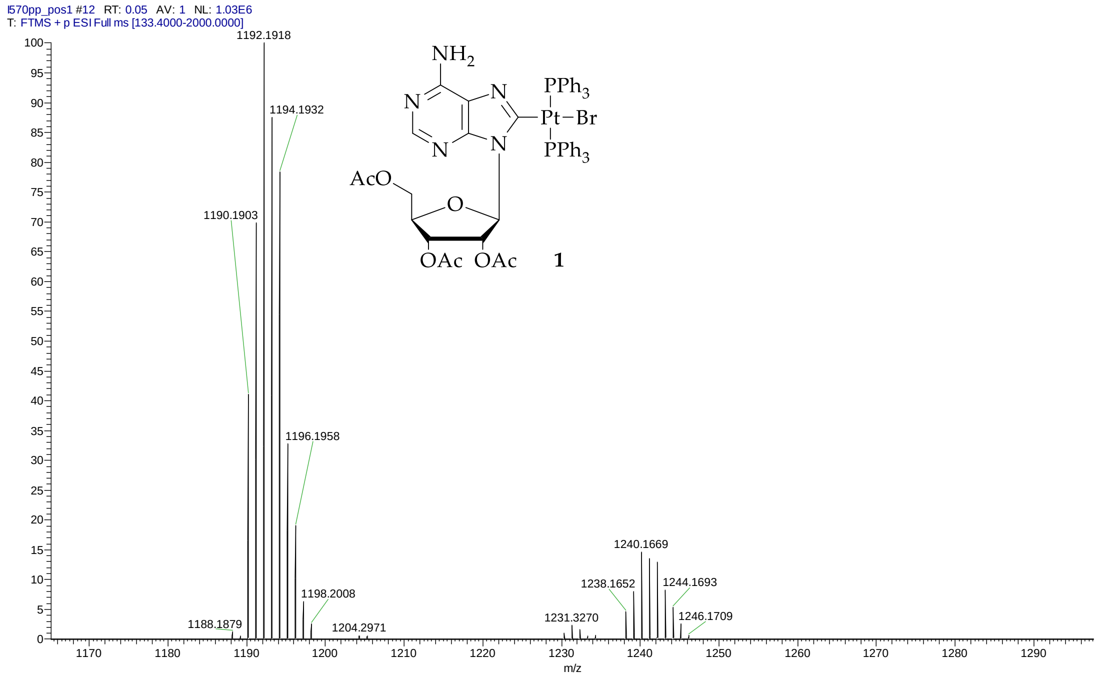

Figure S37. HRMS (ESI) of compound **1** (zoom of m/z 1192.1918).

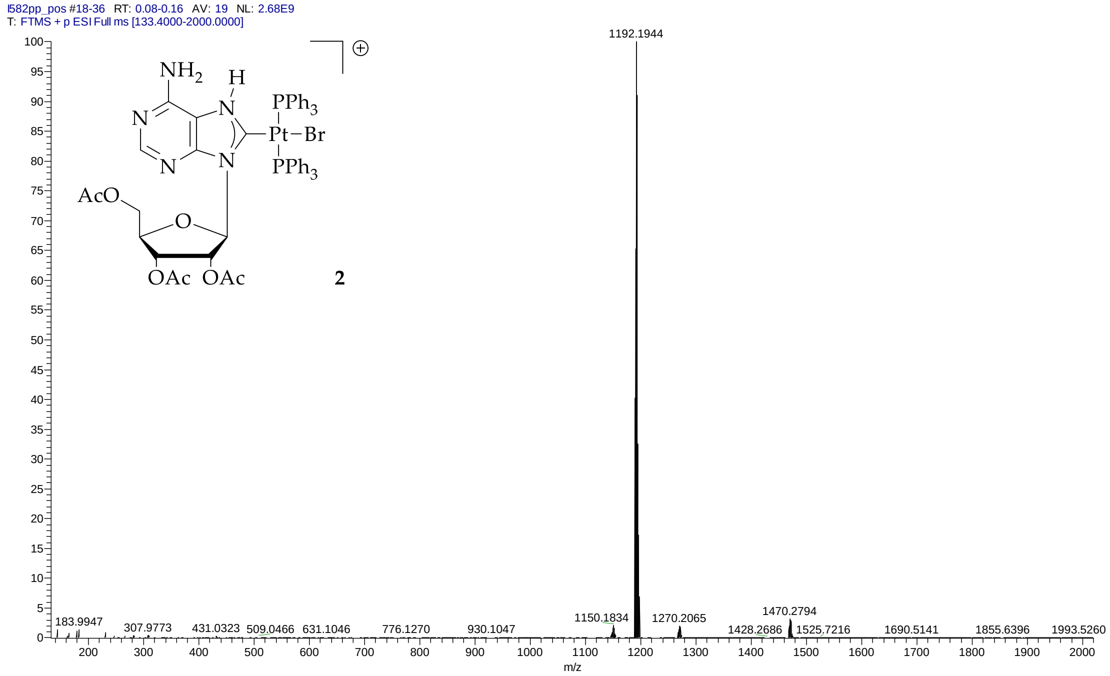

Figure S38. HRMS (ESI) of compound **2** (acquired in positive mode).

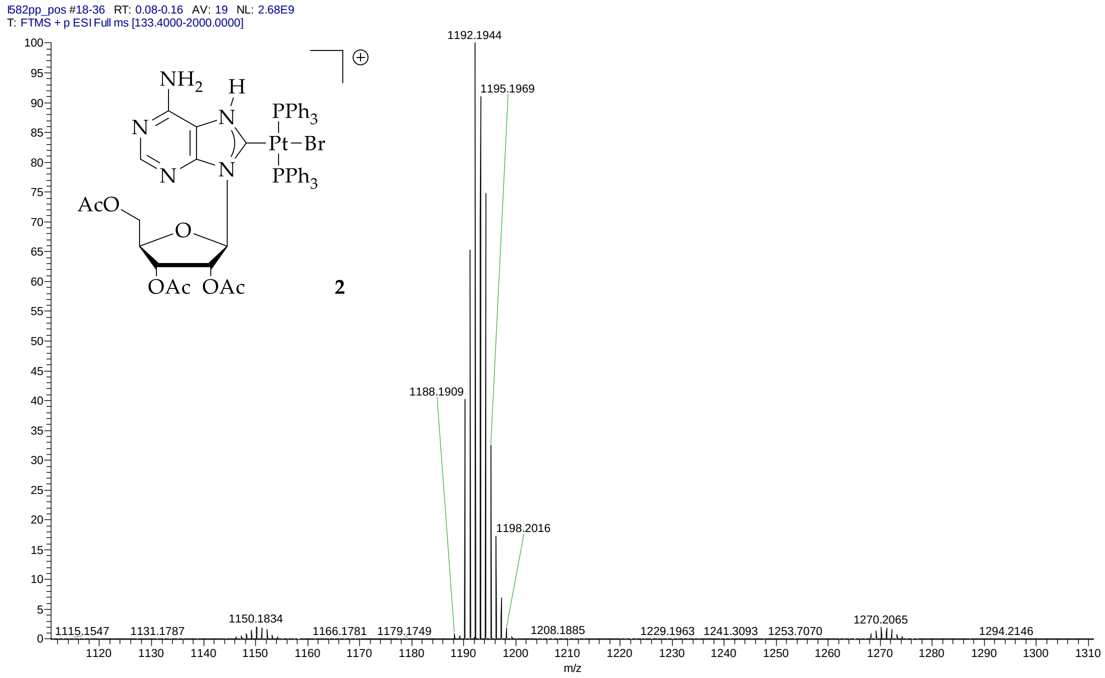

Figure S39. HRMS (ESI) of compound **2** (zoom of m/z 1192.1944).

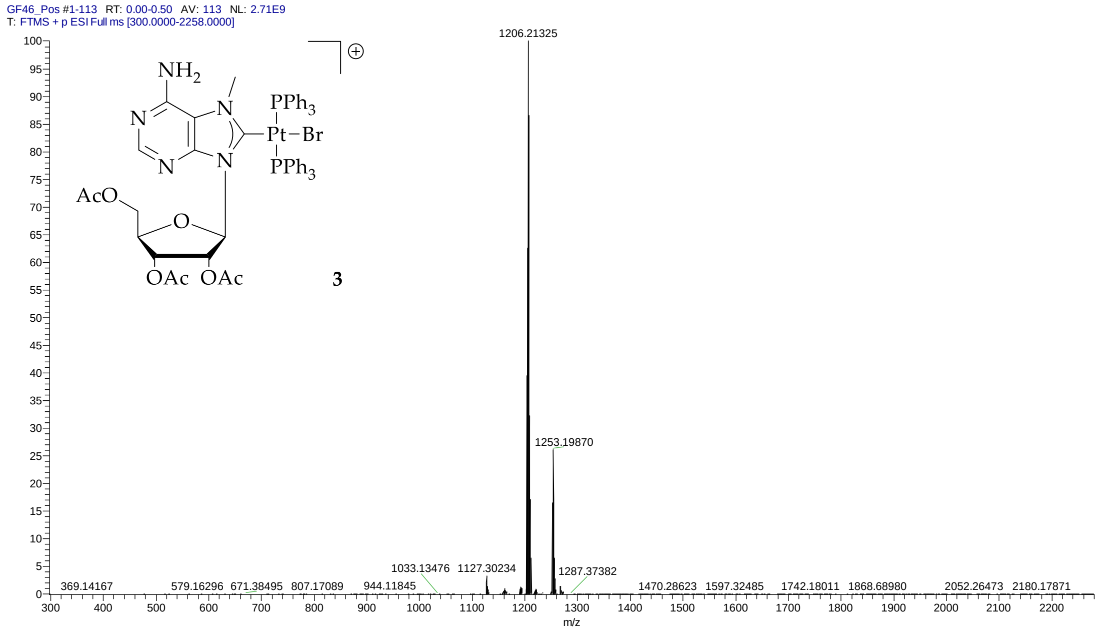

Figure S40. HRMS (ESI) of compound **3** (acquired in positive mode).

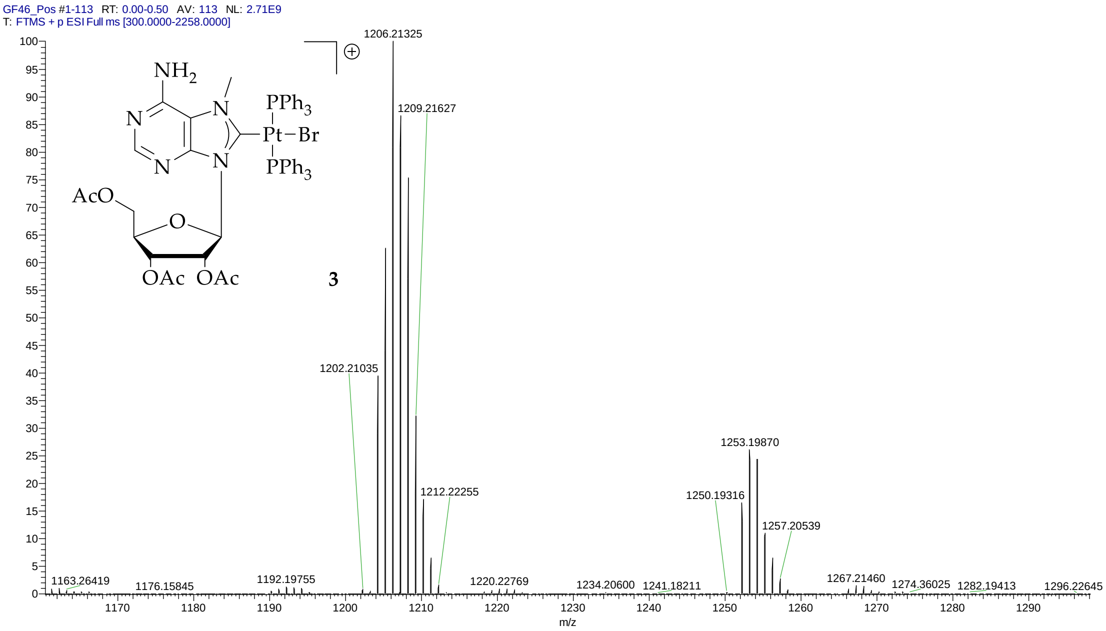

Figure S41. HRMS (ESI) of compound **3** (zoom of m/z 1206.21325).

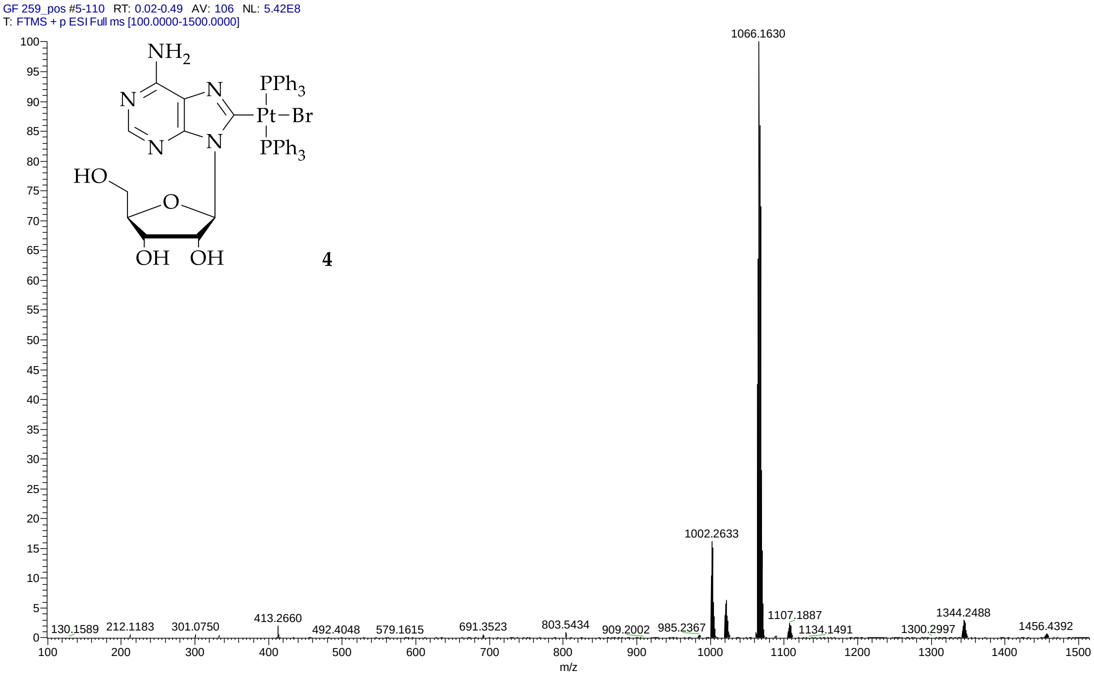

Figure S42. HRMS (ESI) of compound **4** (acquired in positive mode).

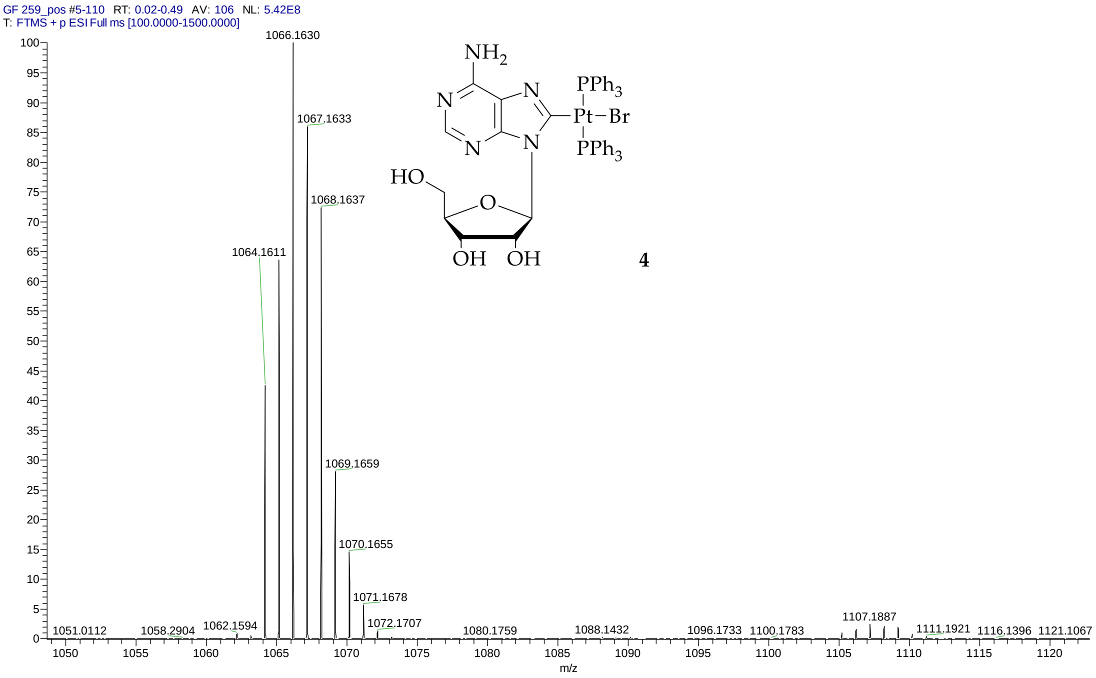

Figure S43. HRMS (ESI) of compound **4** (zoom of m/z 1066.1630).

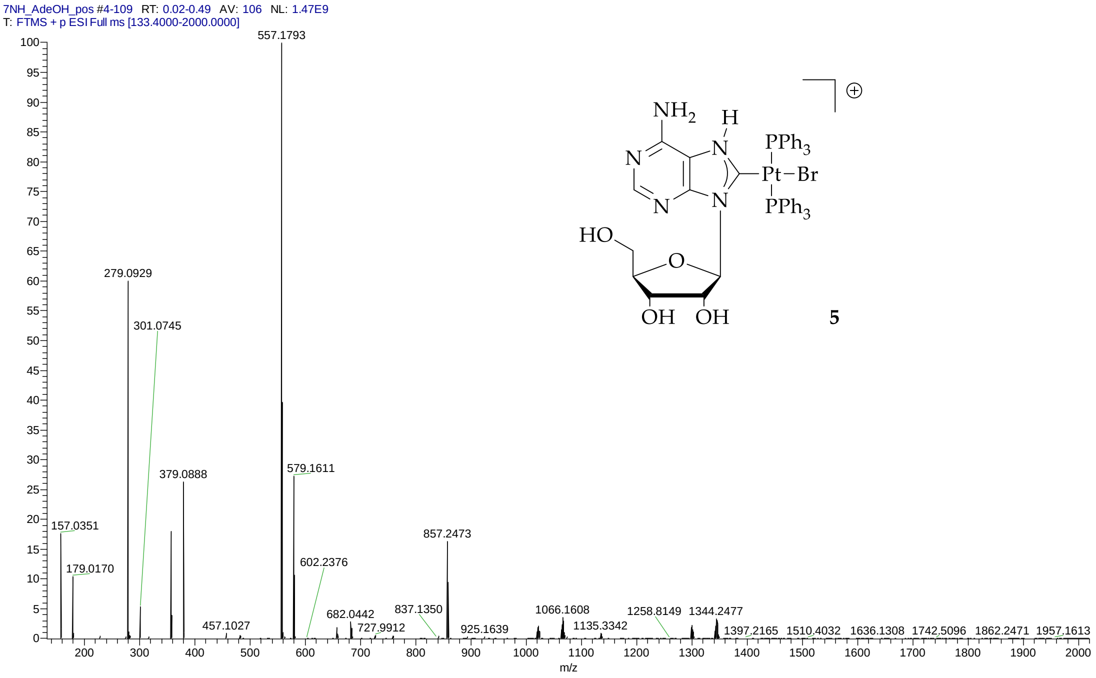

Figure S44. HRMS (ESI) of compound **5** in DMSO (acquired in positive mode).

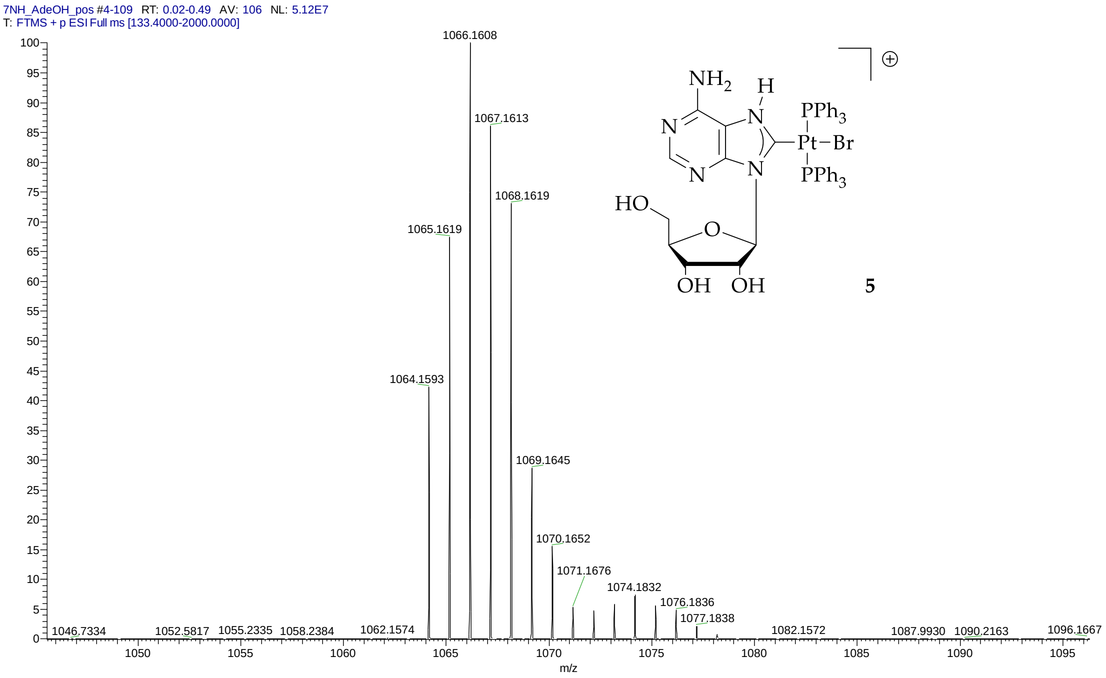

Figure S45. HRMS (ESI) of compound **5** in DMSO (zoom of 1066.1608).

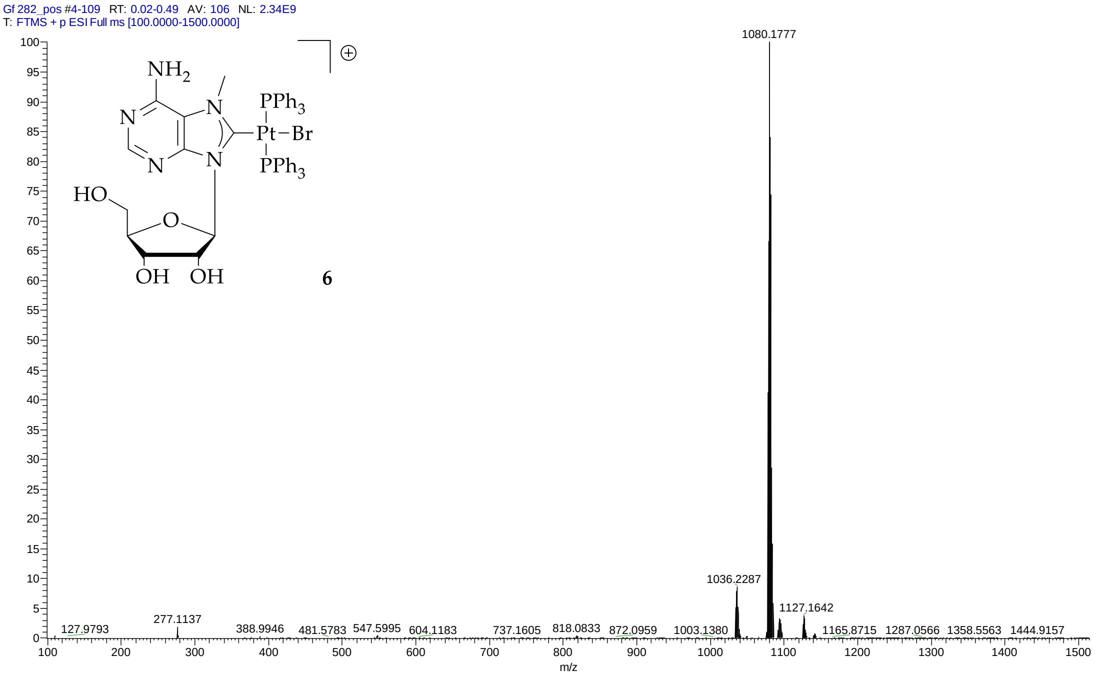

Figure S46. HRMS (ESI) of compound **6** (acquired in positive mode).

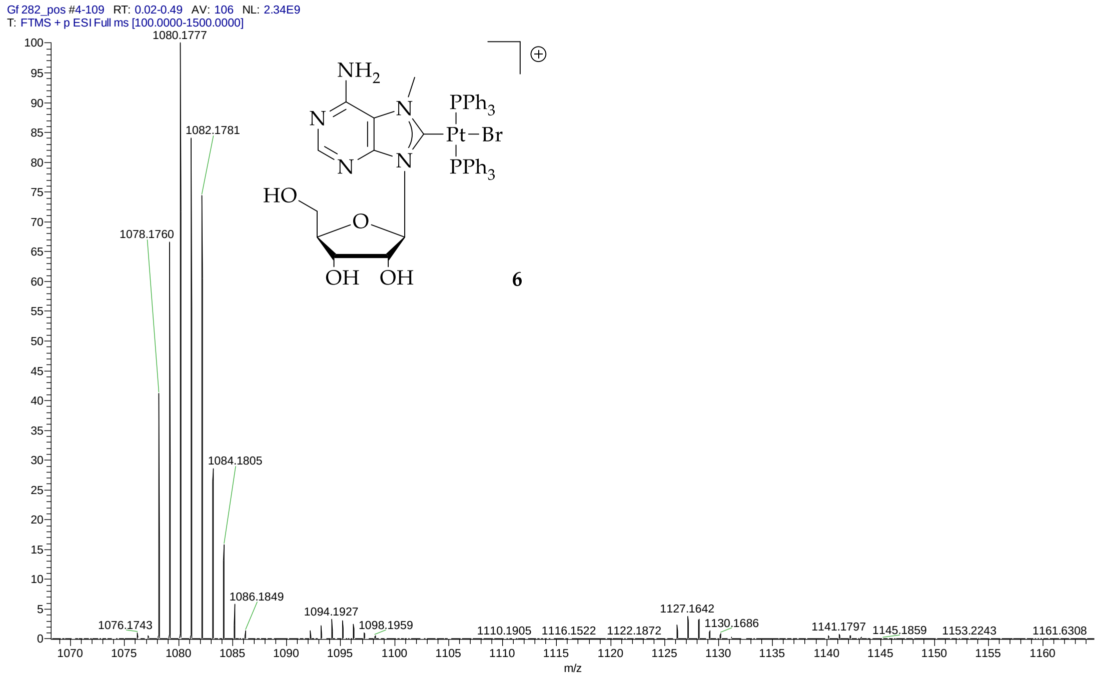

Figure S47. HRMS (ESI) of compound **6** (zoom of 1080.1777).

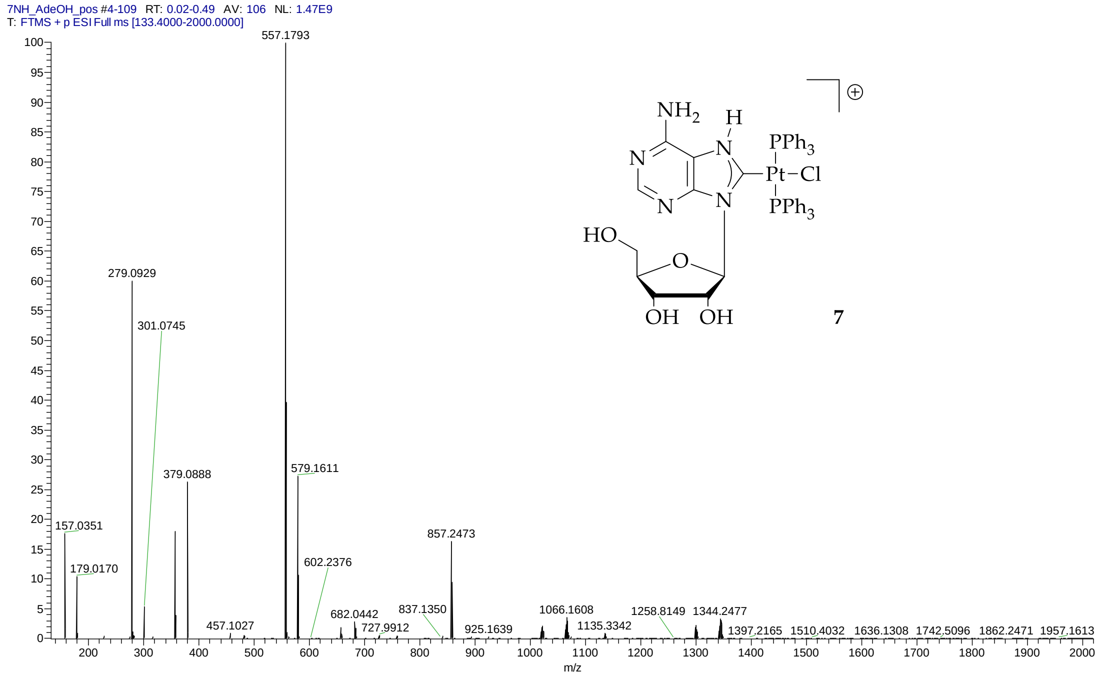

Figure S48. HRMS (ESI) of compound **7** in DMSO (acquired in positive mode).

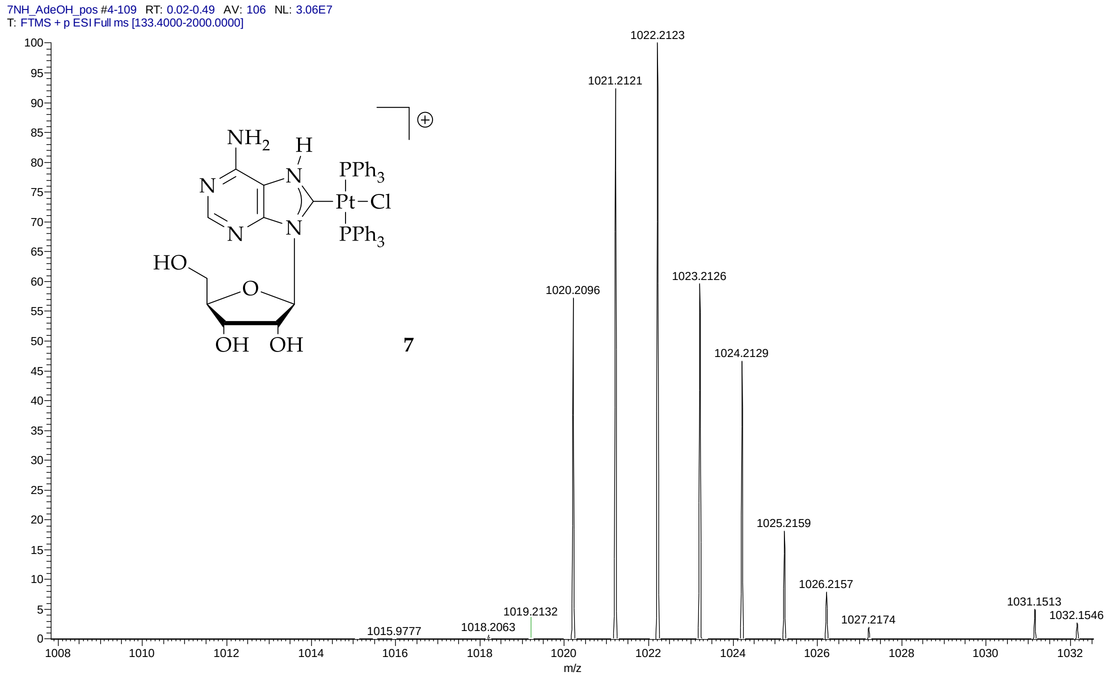

Figure S49. HRMS (ESI) of compound **7** in DMSO (zoom of 1022.2123).

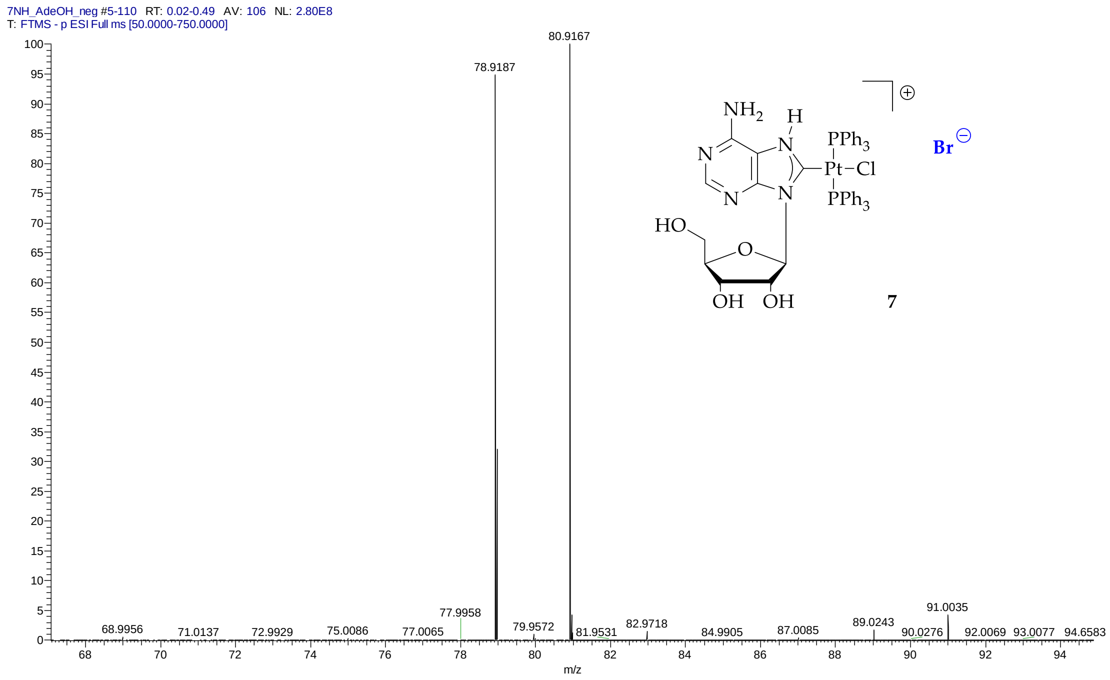

Figure S50. HRMS (ESI) of compound **7** in DMSO (acquired in negative mode).

3. Crystallographic details for complex 2

A crystal suitable for single-crystal X-ray analysis of complex **2** was selected and covered with Fomblin (polyfluoro ether oil) and mounted on a nylon loop. The data was collected at room temperature on a Bruker D8 Venture diffractometer equipped with a Photon 100 CMOS detector, using graphite monochromated Mo-Kα radiation (λ = 0.71073 Å). The data was processed using the APEX3 suite software package, which includes integration and scaling (SAINT), absorption corrections (SADABS [3]) and space group determination (XPREP). Structure solution and refinement were done using direct methods with the programs SHELXT 2014/5 and SHELXL (version 2018/3 [4,5]) inbuilt in APEX, and WinGX-Version 2021.3 [6] software packages. All non-hydrogen atoms were refined anisotropically. Except for NH and NH_2 atoms, all hydrogen atoms were inserted in idealized positions and allowed to refine riding on the parent carbon atom with C–H distances of 0.93 Å, 0.96 Å, and 0.97 Å for aromatic, methyl, and methylene H atoms, respectively. The crystal of **2** showed the presence of disordered solvent molecules, the PLATON/SQUEEZE [7] routine being applied as a good disorder model was impossible to attain. The crystals of **2** also presented racemic twinning, with a Flack parameter of *ca.* 0.50, and thermal disorder, which led to difficulties in the refinement of the structure in the chiral triclinic space group *P*1. Attempts to obtain better crystals were unsuccessful and low temperature measurements led to similar problems. Thus, the structure was refined in the centrosymmetric triclinic space group *P*-1, leading to convergence of the data and being consistent with the remaining analytical data. Restraints were applied to atoms in the disordered chains, but several of the atoms were still not ideally shaped. Nonetheless, a disorder model was not applied, as the resulting structure presented values of chemically impossible bond distances and angles. Therefore, and since the structure was also confirmed by other analytic methods, the authors decided not to apply a disorder model to the structure. The molecular diagrams were drawn with Mercury [8]. Crystal data for **2**: $C_{52}H_{49}BBrF_4N_5O_7P_2Pt$, FW = 1279.70, colourless crystal, 0.30×0.20×0.20, prism, triclinic, space group *P*-1 (no.2), D*c* = 1.362 g cm^{-3}, Z = 2, a = 12.2971(13), b = 13.8228(15), c = 19.730(2) Å, α = 91.563(4), β = 106.251(4), γ = 103.145(4) °, V = 3120.4(6) $Å^3$, T = 296(2) K, ω- and φ-scans, Bruker D8 Venture diffractometer with Photon 100 CMOS area detector, λ (MoK_α) = 0.71073 Å, μ = 2.999 mm^{-1}. Of 93418 reflections measured, 11917 were unique (R_{int} = 0.1340). Refinement on F^2 concluded with the values R_1 = 0.0752 and wR_2 = 0.2122 for 541 parameters and 9250 data with I > 2σI. The asymmetric unit contains one formula unit. The data was deposited in the CCDC under deposit number 2101606.

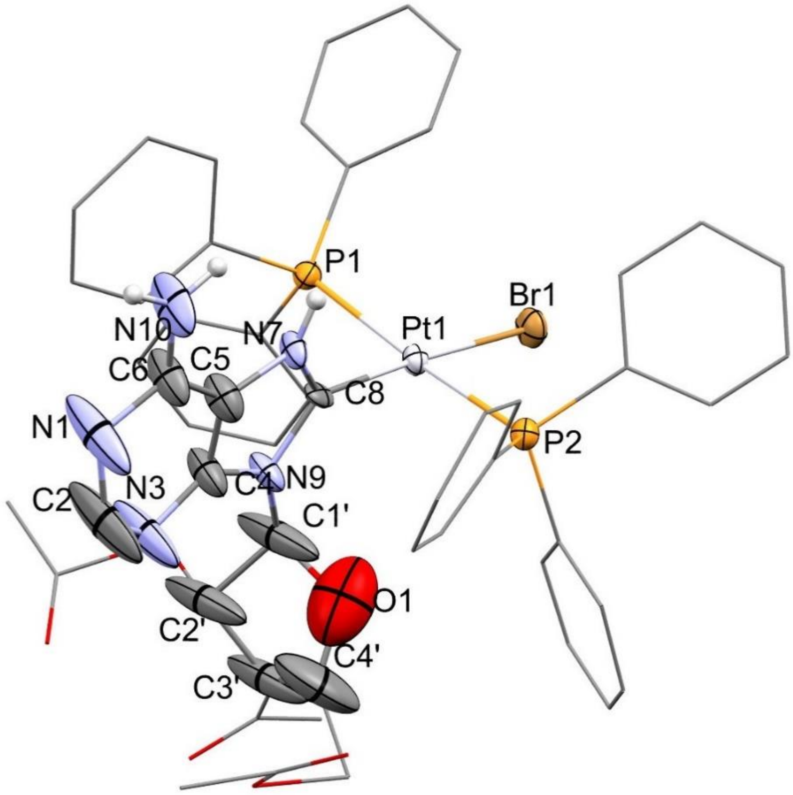

Figure S51. Mercury diagram of compound **2**, using 30% probability level ellipsoids. Except the NH and NH_2, all hydrogen atoms and the counterion BF_4^- were omitted for clarity. Selected bond distances (Å): C8–Pt1 2.020(10); P1–Pt1 2.339(2); P2–Pt1 2.321(2); Br1–Pt1 2.475(1); C8–N7 1.311(11); C8–N9 1.378(12). Selected bond angles (°): P1−Pt1−Br1 91.37(7), P2−Pt1−Br1 88.58(6), C8−Pt1−Br1 175.8(2), P1−Pt1−P2 175.67(8), C8−Pt1−P1 89.5(3), C8−Pt1−P2 90.9(3), N7−C8−N9 110.0(9), C8−N7−C5 107.3(9), C8−N9−C4 106.0(9)

4. Measurement of the stability of Ade$_{Ac}$ by ^{1}H NMR

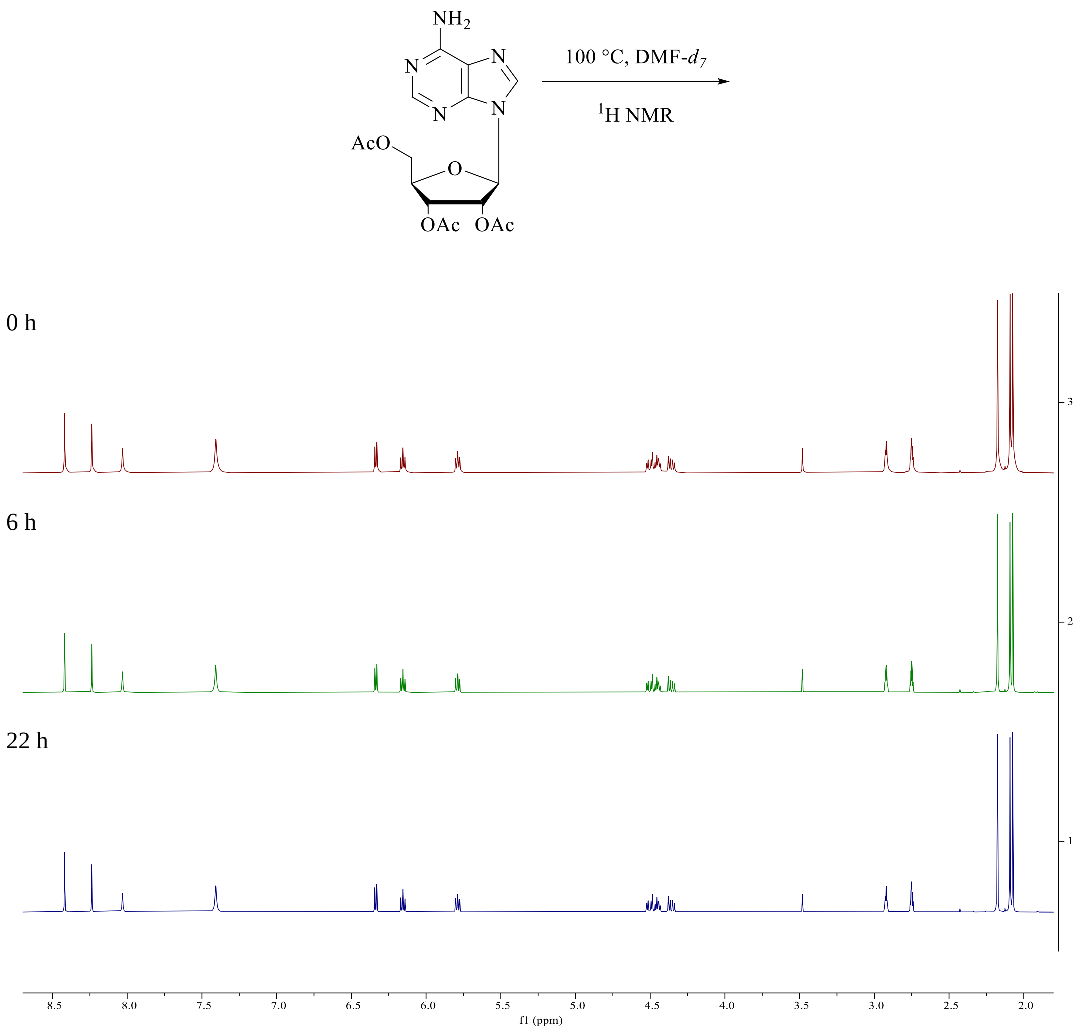

Figure S52. ^{1}H NMR spectrum of **Ade$_{Ac}$** in deuterated DMF after heating at 100 °C for different time periods (0 h, 3 h and 22 h).

5. Measurement of the stability of 1 by ¹H NMR

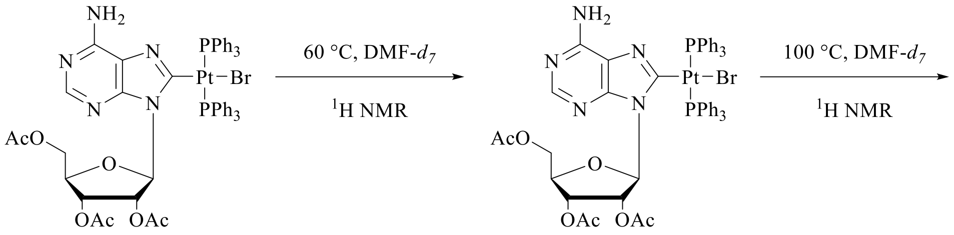

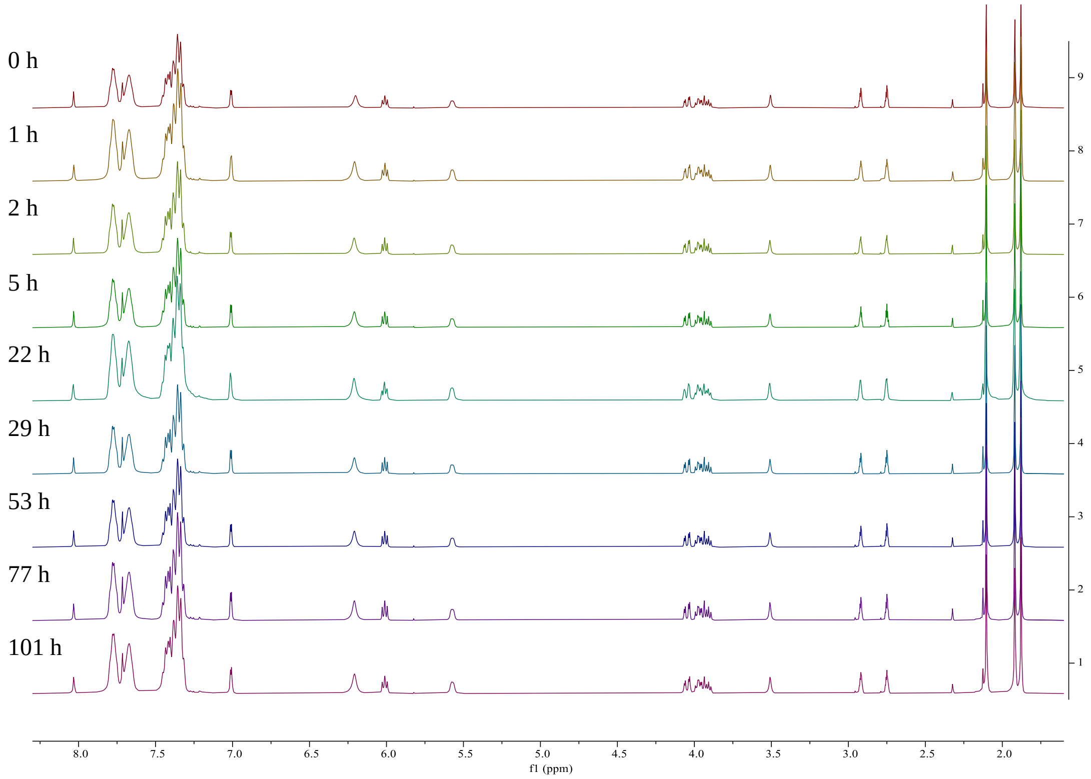

Figure S53. ¹H NMR spectrum of complex **1** in deuterated DMF after heating at 60 °C for different time periods (0 h, 1 h, 2 h, 5 h, 22 h, 29 h, 53 h, 77 h and 101 h).

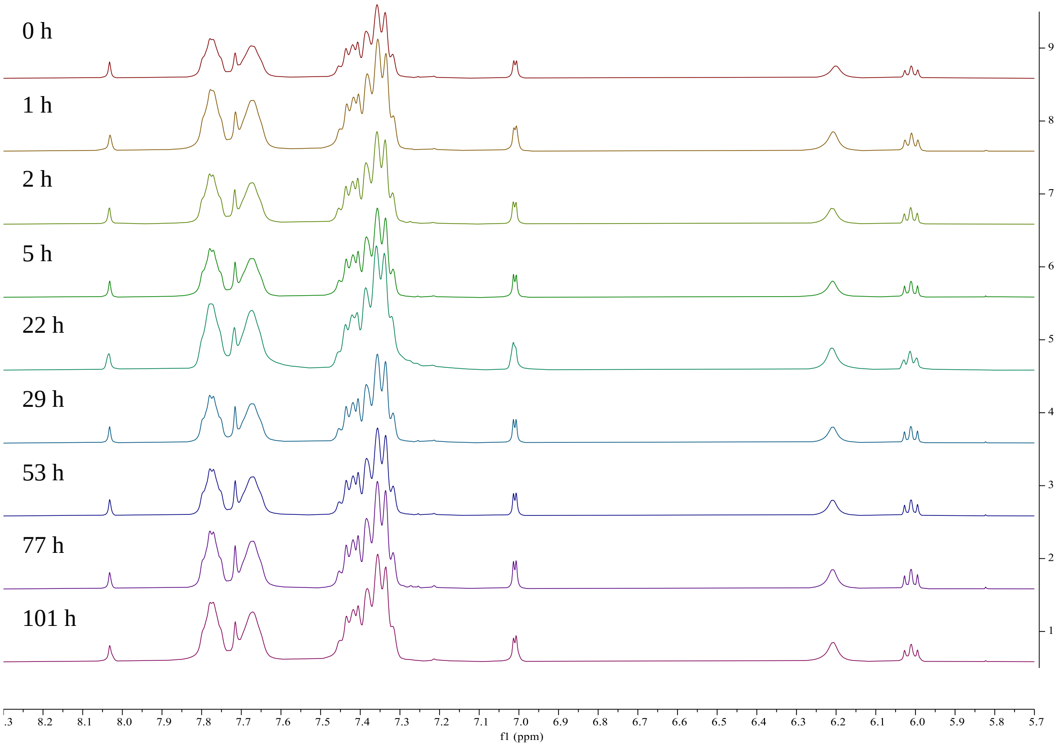

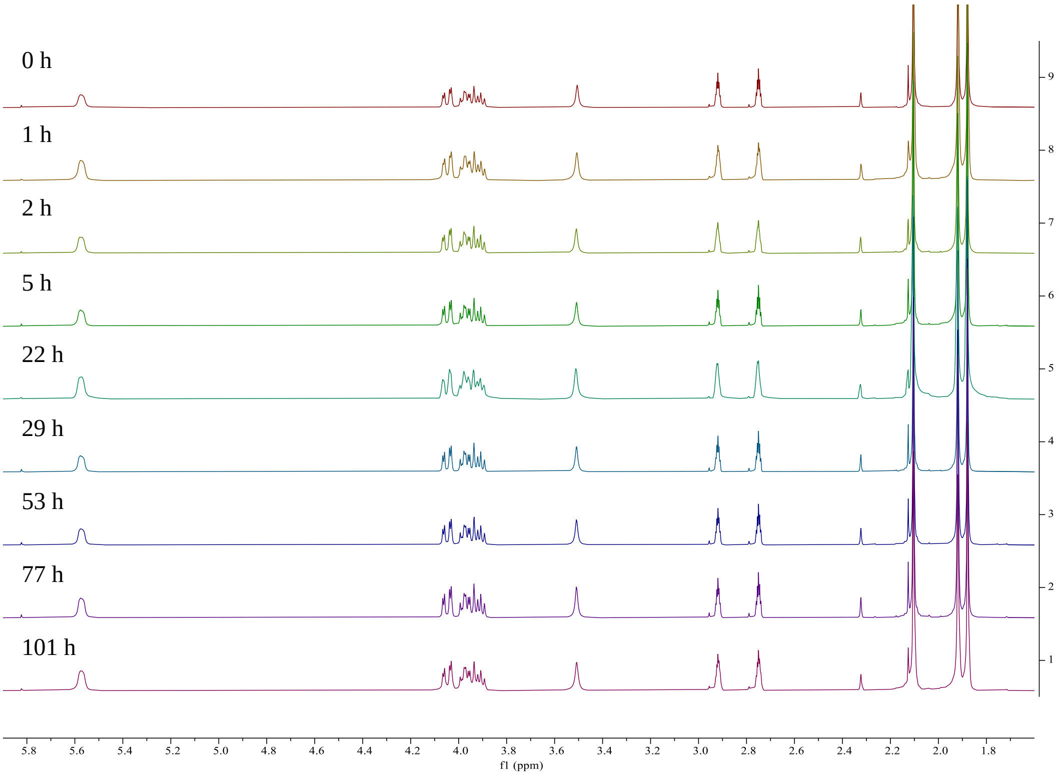

Figure S54. Selected areas (8.3 to 5.7 and 5.9 to 1.6 ppm) of the ^{1}H NMR spectrum of complex **1** in deuterated DMF, showing with greater detail all signals, after heating at 60 °C for different time periods (0 h, 1 h, 2 h, 5 h, 22 h, 29 h, 53 h, 77 h and 101 h).

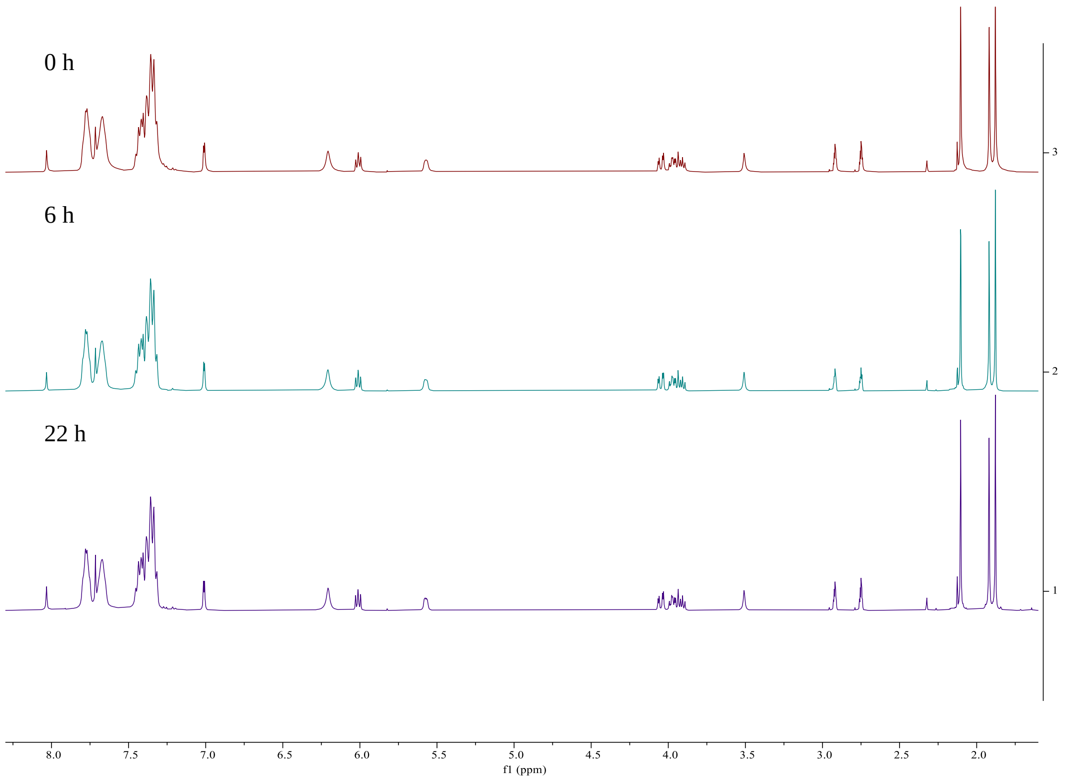

Figure S55. ^{1}H NMR spectrum of complex **1** in deuterated DMF after heating at 100 °C for different time periods (0 h, 6 h and 22 h). This sample was previously heated at 60 °C for 101h. Since no signs of degradation were detected, the temperature was then increased to 100 °C.

6. Measurement of the stability of 5 in DMSO-d_6 by ^{1}H NMR

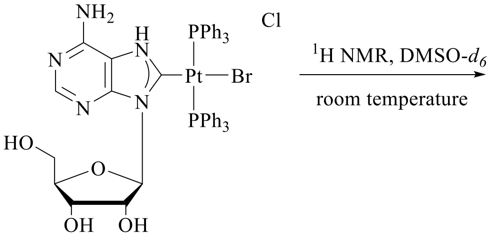

5

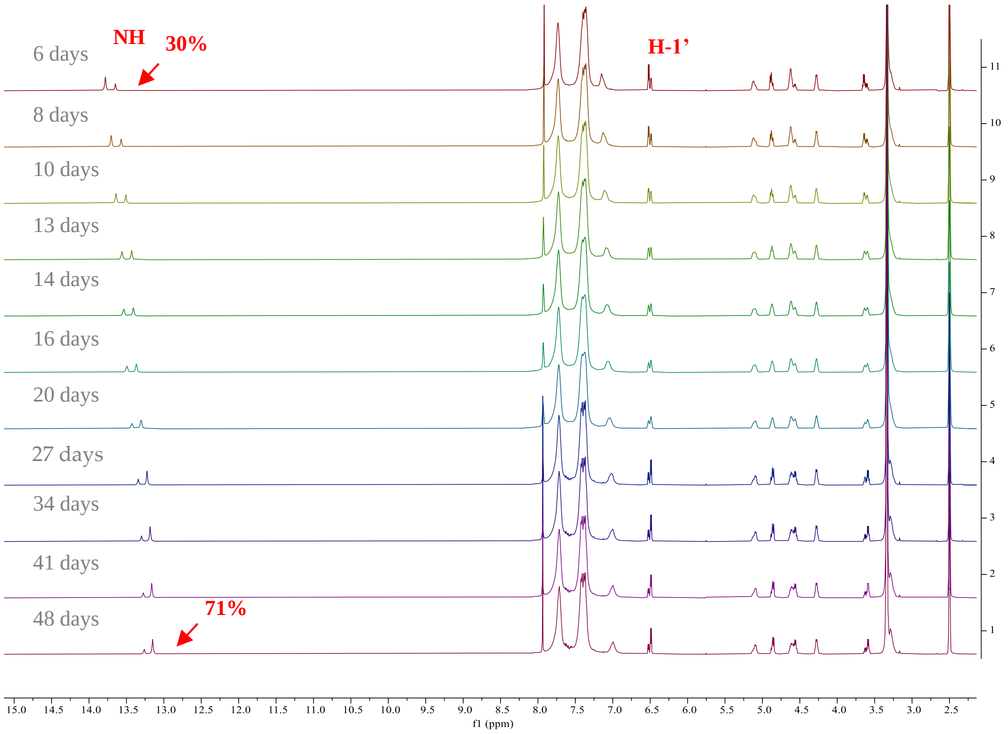

Figure S56. ^{1}H NMR spectra of complex **5** in deuterated DMSO at room temperature during different times (6 to 48 days). The mentioned percentages were calculated considering the signals for both NH and H-1′, which were in accordance.

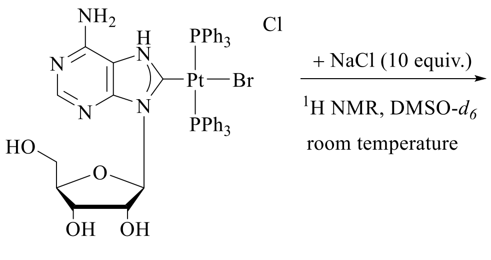

5

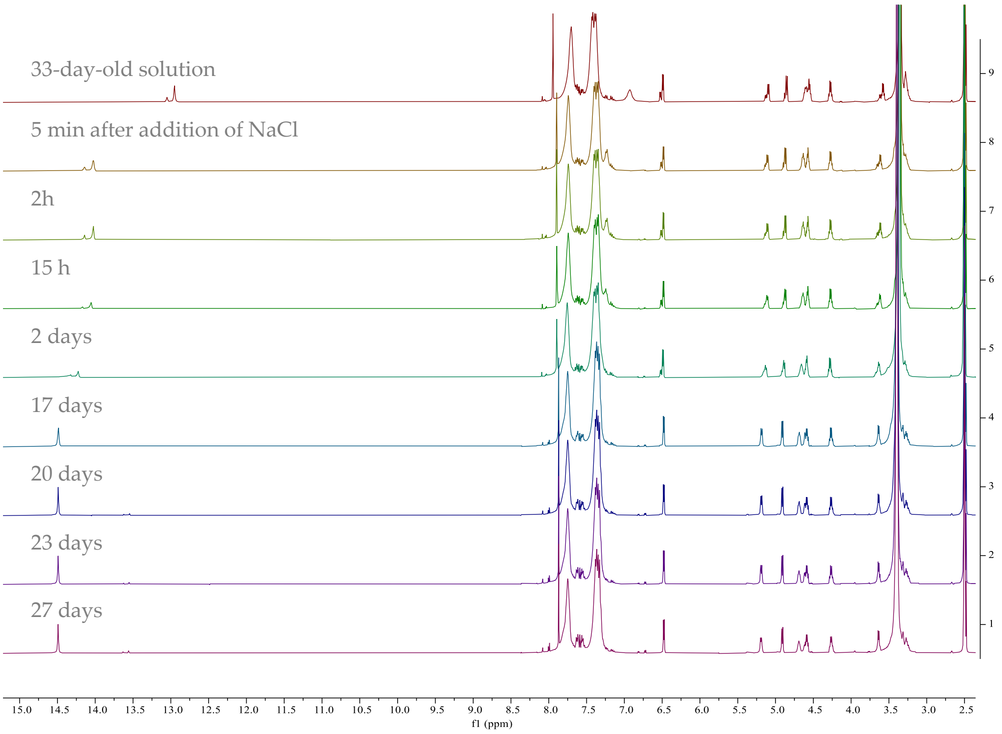

Figure S57. Evolution of the ^{1}H NMR spectrum of complex **5** in deuterated DMSO at room temperature during different times after addition of 10 equivalents of NaCl to a 33-day-old solution.

7. References

1. Ikehara, M.; Kaneko, M. Studies of Nucleosides and Nucleotides-XLI. Purine Cyclonucleosides-8 Selective Sulfonylation of 8-Bromoadenosine Derivatives and an Alternate Synthesis of 8,2′- and 8,3′-S-Cyclonucleosides. *Tetrahedron* **1970**, *26* (18), 4251–4259. https://doi.org/10.1016/S0040-4020(01)93068-6.
2. Ikehara, M.; Yamada, S. Studies of Nucleosides and Nucleotides. XLIX. Synthesis of 8-Fluoroadenosine. *Chem. Pharm. Bull. (Tokyo).* **1971**, *19* (1), 104–109. https://doi.org/10.1248/cpb.19.104.
3. Krause, L.; Herbst-Irmer, R.; Sheldrick, G. M.; Stalke, D. Comparison of Silver and Molybdenum Microfocus X-Ray Sources for Single-Crystal Structure Determination. *J. Appl. Cryst.* **2015**, *48* (1), 3–10. https://doi.org/10.1107/S1600576714022985.
4. Sheldrick, G. M. Crystal Structure Refinement with SHELXL. *Acta Crystallogr. C Struct. Chem.* **2015**, *71* (1), 3–8. https://doi.org/10.1107/S2053229614024218.
5. Hübschle, C. B.; Sheldrick, G. M.; Dittrich, B. ShelXle: A Qt Graphical User Interface for SHELXL. *J. Appl. Crystallogr.* **2011**, *44* (6), 1281–1284. https://doi.org/10.1107/S0021889811043202.
6. Farrugia, L. J. WinGX and ORTEP for Windows: An Update. *J. Appl. Crystallogr.* **2012**, *45* (4), 849–854. https://doi.org/10.1107/S0021889812029111.

7. Spek, A. L. PLATON SQUEEZE: A Tool for the Calculation of the Disordered Solvent Contribution to the Calculated Structure Factors. *Acta Crystallogr. C Struct. Chem.* **2015**, *71* (1), 9–18. https://doi.org/10.1107/S2053229614024929.
8. Macrae, C. F.; Sovago, I.; Cottrell, S. J.; Galek, P. T. A.; McCabe, P.; Pidcock, E.; Platings, M.; Shields, G. P.; Stevens, J. S.; Towler, M.; Wood, P. A. Mercury 4.0: From Visualization to Analysis, Design and Prediction. *J. Appl. Crystallogr.* **2020**, *53* (1), 226–235. https://doi.org/10.1107/S1600576719014092.
